# Use of universal primers for the 18S ribosomal RNA gene and whole soil DNAs to reveal the taxonomic structures of soil nematodes by high-throughput amplicon sequencing

**DOI:** 10.1371/journal.pone.0259842

**Published:** 2021-11-15

**Authors:** Harutaro Kenmotsu, Emi Takabayashi, Akinori Takase, Yuu Hirose, Toshihiko Eki

**Affiliations:** 1 Molecular Genetics Laboratory, Department of Applied Chemistry and Life Science, Toyohashi University of Technology, Toyohashi, Aichi, Japan; 2 Research Center for Agrotechnology and Biotechnology, Toyohashi University of Technology, Toyohashi, Aichi, Japan; University of Limpopo, SOUTH AFRICA

## Abstract

Nematodes are abundant metazoans that play crucial roles in nutrient recycle in the pedosphere. Although high-throughput amplicon sequencing is a powerful tool for the taxonomic profiling of soil nematodes, polymerase chain reaction (PCR) primers for amplification of the 18S ribosomal RNA (SSU) gene and preparation of template DNAs have not been sufficiently evaluated. We investigated nematode community structure in copse soil using four nematode-specific (regions 1–4) and two universal (regions U1 and U2) primer sets for the SSU gene regions with two DNAs prepared from copse-derived mixed nematodes and whole soil. The major nematode-derived sequence variants (SVs) identified in each region was detected in both template DNAs. Order level taxonomy and feeding type of identified nematode-derived SVs were distantly related between the two DNA preparations, and the region U2 was closely related to region 4 in the non-metric multidimensional scaling (NMDS) based on Bray-Curtis dissimilarity. Thus, the universal primers for region U2 could be used to analyze soil nematode communities. We further applied this method to analyze the nematodes living in two sampling sites of a sweet potato-cultivated field, where the plants were differently growing. The structure of nematode-derived SVs from the two sites was distantly related in the principal coordinate analysis (PCoA) with weighted unifrac distances, suggesting their distinct soil environments. The resultant ecophysiological status of the nematode communities in the copse and field on the basis of feeding behavior and maturity indices was fairly consistent with those of the copse- and the cultivated house garden-derived nematodes in prior studies. These findings will be useful for the DNA metabarcoding of soil eukaryotes, including nematodes, using soil DNAs.

## Introduction

Soil ecosystem is composed of microbiota and soil [1]. Soil biota is influenced by soil environments because soils are heterologous with different chemical and physical properties, including pH, nutrients, and water contents. Therefore, the taxonomic composition of soil organisms reflects the environmental conditions of soils. Soil biota, especially microbiome, is influenced by plants (crops) [2, 3]. Thus, quantitative information on the taxa of soil organisms is useful for assessing soil environments, including agricultural soils. Nematodes are abundant animals with various species and feeding habitats and are widely distributed in freshwater, marine, and terrestrial environments [4–7]. They play a crucial role in soil nutrient recycling [8, 9] and occupy the ecological position in the pedosphere comparable to that of planktons in the hydrosphere. Nematode taxonomic compositions are influenced by ecosystem type and various factors, such as food availability and abundance, physical, and chemical parameters (e.g., pH, temperature) [8, 9], soil properties [9, 10], latitude [11, 12], and agricultural conditions (e.g., tillage, cultivated plants, fertilizers) [13–22]. Thus, the taxonomic composition and abundance of nematodes have been used as an indicator of biological, environmental, and toxicological conditions in soils [4, 23, 24].

Traditional morphology-based methods of nematode taxonomic identification are laborious, require high skills and experience, and produce poor analysis throughput. Thus, several methods have been developed [25, 26]. DNA barcoding can accurately identify species on the basis of the nucleotide sequences of DNA barcodes without the need for special experience on nematode morphologies [27]. Prior research by our laboratory demonstrated the presence of distinct nematode taxonomic compositions in soybean-cultivated agricultural and unmanaged follower bed soils using DNA barcoding by Sanger sequencing of the 18S small subunit ribosomal RNA (SSU) gene and the cytochrome *c* oxidase I (COI) gene from individual nematodes [28]. In this study, the nucleotide sequences of polymerase chain reaction (PCR)-amplified barcode DNAs from individual nematodes were determined using Sanger-based DNA sequencing; the nematodes were then classified by their sequences into taxonomic groups sharing identical DNA barcode sequences, known as operational taxonomic units (OTUs). The number of OTUs indicates the number of taxonomic groups (i.e., nematode species diversity), and the number of nematodes in each OTU shows the proportion of each taxonomic group in the nematode community. Despite successful profiling of soil nematode taxa, DNA barcoding based on one-by-one sequencing is laborious and time consuming, and the resultant quantitative data of nematode-derived OTUs are insufficient and hardly cover the taxa of nematodes comprehensively because of limited sample processing capacity. Recent advancement in massive DNA sequencing technology allows developing the taxonomic profiling of organisms using the high-throughput amplicon sequencing of DNA barcodes. This method has been further applied to the taxonomic analysis of soil nematodes. To assess suitable regions for this method among four SSU gene regions (regions 1–4, 337–388 base pair (bp) in length), Kenmotsu et al. (2020) clarified the taxa of 68 out of 96 individual nematodes isolated from copse soil using high-throughput amplicon sequencing via the Illumina MiSeq platform [29]. In this study, region 4, located at the 3ʹ-region of the SSU gene, was suggested as the most suitable barcode among the four regions because of the identification of the large number of nematode-derived sequence variants (SVs) and sufficient reference sequence coverage in the DNA barcoding of individual nematodes. Recently, Kenmotsu et al. have applied high-throughput amplicon sequencing using the genomic DNAs of complex nematodes isolated from three sites (i.e., copse, uncultivated field, and zucchini-cultivated house garden) and successfully clarified distinct proportions of nematode taxa and the ecophysiological status of nematode families in each site [30]. This study has also suggested that region 4 is the most suitable for the DNA barcoding of complex mixed nematodes among the four regions.

Currently, DNA barcoding of soil nematodes is performed using the high-throughput sequencing of SSU gene-derived amplicons. Nematode-specific SSU primers are usually used for amplifications from genomic DNAs purified from complex soil nematodes [11, 20, 22, 30, 31]. Moreover, universal SSU primers have been recently developed for the DNA barcoding of eukaryotes through high-throughput amplicon sequencing [32, 33]. The amplicons by the universal SSU primers contain more heterologous species than those by the taxa-specific primers, making them suitable for DNA metabarcoding. Therefore, taxonomic analyses of soil nematode communities have been conducted through this method using universal primers and complex nematode genomic DNAs [12, 21, 34]. In several studies, whole soil DNAs has been used for amplifications in place of nematode genomic DNAs [35–38]. However, studies have yet to determine whether or not these universal primers amplify nematode-derived DNAs as observed in the amplicons using nematode-specific primers or if consistent results are obtained from the analyses using soil and genomic DNAs of nematodes isolated from the soil. In the present work, approximately 340–450 bp fragments of the SSU gene were prepared by PCR using two universal primer sets for regions U1 and U2 in addition to four previously evaluated nematode-specific primer sets for regions 1–4. These fragments were subjected to Illumina sequencing to investigate whether or not the universal primers can be used in place of nematode-specific primers for the taxonomic profiling of soil nematodes. Soil DNAs can be directly prepared from soil samples and likely contain more nematode species because of purification without the bias-prone process of nematode isolation. Therefore, we simultaneously tested whole soil DNAs as template DNAs for amplifying the six regions-derived fragments and genomic DNAs from nematodes isolated from copse soils. Furthermore, the taxa of nematode communities in the agricultural field were investigated by massive amplicon sequencing using the universal primers and soil DNAs derived from two sites in the field, where the plants were differently growing. This study could guide researchers working on the DNA metabarcoding of soil eukaryotes, including nematodes, using soil DNAs.

## Materials and methods

### Experimental design

**Fig 1:**
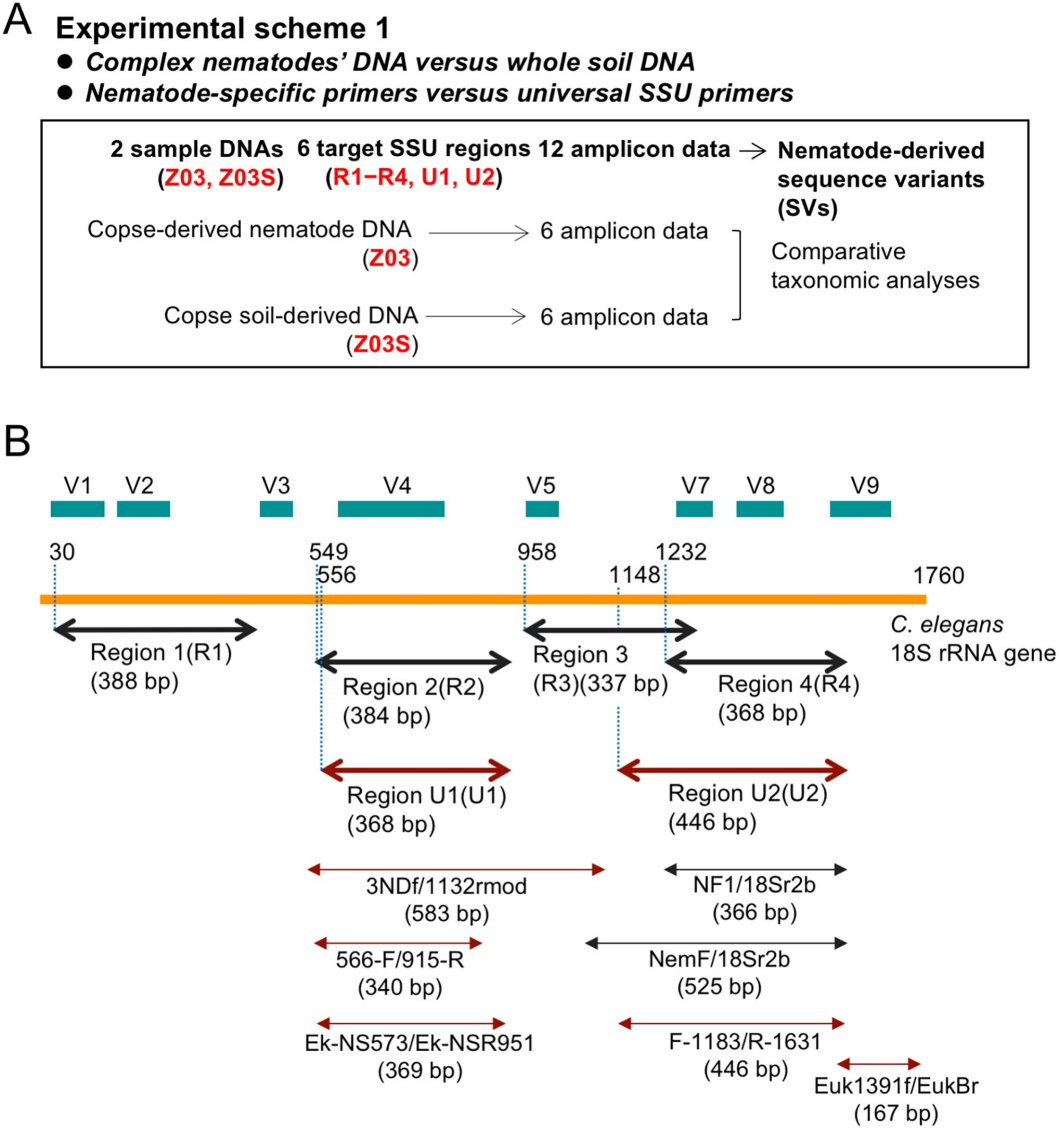
Experimental scheme 1 for the copse-derived nematodes and PCR target regions of the 18S ribosomal RNA gene. The experimental scheme of the high-throughput sequencing of 12 amplicons prepared from six regions of the 18S ribosomal RNA (SSU) gene using the copse-derived nematode genomic DNA and whole soil DNA is shown (A). Red colored codes in parentheses for sample DNA (e.g., Z03) represent the nematode genomic DNA with the soil sample code (Z: copse) and the experimental ID (03), and the code Z03S indicates the soil DNA purified from the copse soil in the same experiment (S: soil DNA). R1–R4, U1 and U2 in red are the abbreviations of PCR target regions in this study (A). Six PCR target regions with their abbreviations and sizes of amplicons are shown by bold double-headed arrows in gray for nematode-specific primers and in dark red for universal primers (B). The numbers indicating the nucleotide positions of the 5ʹ-end of forward primers are shown on the entire SSU gene prepared from the nucleotide sequence of *C*. *elegans* ribosomal RNA gene cluster (X03680). Teal colored boxes correspond to the hypervariable regions of eukaryotic SSU genes reported by Hugerth et al. [33] and Hadziavdic et al. [32]. The regions amplified by two published nematode-specific primer sets (NF1/18Sr2b and NemF/18Sr2b) [35, 60] and four eukaryotic universal primer sets (3NDf/1132rmod [34], 566-F/915-R [37], Ek-NS573/Ek-NSR951[58, 64], F-1183/R-1631 [58, 65], Euk1391f/EukBr [36, 66, 67]) are also indicated by double-headed arrows in gray and dark red with sizes of amplicons, respectively. Fig 1B has been modified and prepared from the Fig 1B appearing in our previous paper [29].

**Fig 2:**
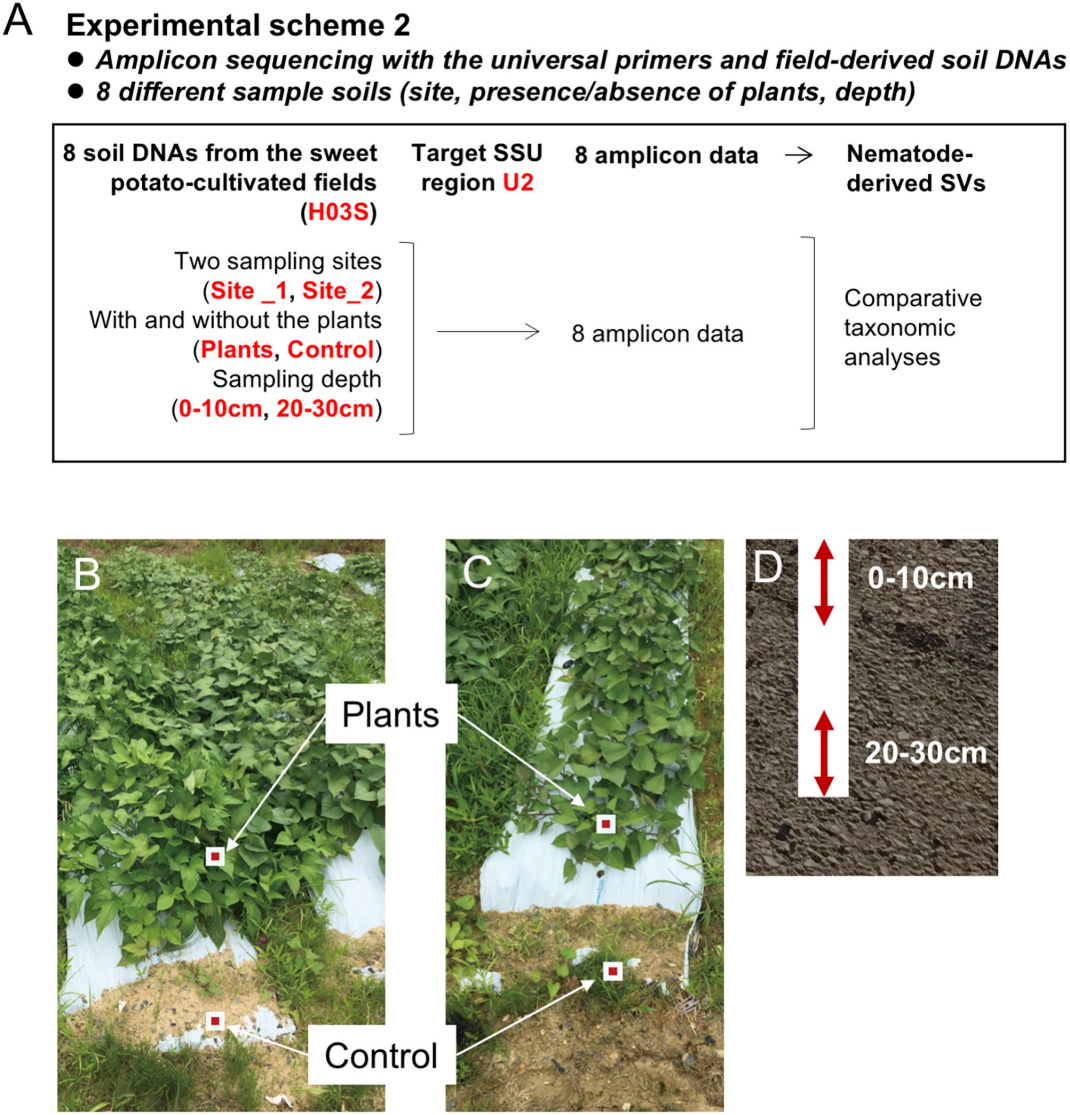
Experimental scheme 2 for the field-derived nematodes by amplicon sequencing using the universal primers and soil DNA. The experimental scheme of the high-throughput sequencing of amplicons prepared using the agricultural field-derived soil DNA (H03S) and the universal SSU primers for region U2 is shown (A). Soil samples were isolated from the surface (0–10 cm in depth) and deep (20–30 cm) layers (D) of the distal (control: without plants) and proximal (plants: with plants) points to the growing sweet potato (B, C) as indicated by red/white squares, at two different sites in the field (B: site 1 and C: site 2). Growth of the plants at site 1 (B) was apparently dominated compared with that at site 2 (C). Regarding the code H03S for template DNA, see the legend for Fig 1.

Two experiments were performed in this study (Figs 1 and 2): 1) testing of the suitable universal primers of the 18S ribosomal RNA (SSU) gene and soil DNA used as template DNA for high-throughput amplicon sequencing to analyze the soil nematode communities and 2) application of the developed method for taxonomic profiling of nematodes in the agricultural soils with different depth and plantation. In the experiment 1, we tested six primer sets amplifying each SSU region by PCR: four nematode-specific primer sets for regions 1–4 [29, 30] and two universal eukaryotic primer sets for regions U1 and U2 (Fig 1B). In parallel, we compared the taxonomic profiles of the nematodes living in the copse soils using the DNA prepared from whole soils and complex nematodes isolated by flotation sieving method [28]. Nematode taxa derived from 12 amplicons generated by six primer sets and two template DNAs were investigated (Fig 1A). In the experiment 2, we conducted the taxonomic profiling of nematodes through high-throughput amplicon sequencing using the universal primer set and whole soil DNA in the sweet potato-cultivated field (Fig 2A). We analyzed eight soil samples used for DNA purification were isolated from the surface and deep layers (Fig 2D) at the sampling points with (plants) and without (control) sweet potato at two sites, where the plants were growing differently (Fig 2B and 2C) in the agricultural field. These experiments were performed once.

### Soil sampling

Copse soil samples for the experiment 1 were collected from the copse on the campus of Toyohashi University of Technology [29, 30] in July 2017 under clear climatic conditions (temperature 32°C and humidity 77%) in Toyohashi, Japan (137° 24’E, 34° 42’N). The copse soil was sampled to a depth of 20–30 cm using a soil sampling auger (2.5 cm in diameter, Fujiwara Scientific Co., Tokyo, Japan). Field soil samples for the experiment 2 were collected in July 2018 under cloudy conditions (temperature 32ºC and humidity 55%) from two sampling sites (i.e., sites 1 and 2) in the sweet potato-cultivated field managed by the Research Center for Agrotechnology and Biotechnology of the Toyohashi University of Technology [30]. Growth of plants at site 1 (Fig 2B) was apparently dominated compared with that at site 2 (Fig 2C). Four soil samples in each site were collected from the surface (0–10 cm in depth) and deep (20–30 cm) layers at two sampling points (Fig 2B–2D) without (approximately 40 cm apart from the plants; designated as “control”) and with (within 10 cm from the plants; designated as “plants”) plants. For both experiment 1 and 2, over-sized contaminants (e.g., stones and plant roots) were removed by filtering the samples through a 0.7 mm sieve; the resulting samples were used for nematode isolation or purification of whole soil DNAs within a day of collection.

### Preparations of nematode and whole soil DNAs

Nematodes were isolated from 10 g copse soils using the improved flotation sieving method with colloidal silica in accordance with the method described by Morise et al. [28], and this procedure was repeated four times. Whole nematodes from approximately 40 g soil samples were trapped on sieves and eluted into water in a watch glass. The nematodes were then picked up using a micropipette (P-20, Gilson, Middleton, WI, USA) with a cut tip under an SZX16 stereomicroscope (Olympus) and collected into a DNA LoBind tube (Eppendorf, Hamburg, Germany). Genomic DNAs from whole nematodes isolated from copse soils were purified using a DNeasy PowerSoil Kit (QIAGEN, Venlo, Netherlands) in accordance with the manufacturer’s instructions. Whole soil DNAs were prepared from 10 g fresh copse and field soils using a DNeasy PowerMax Soil Kit (QIAGEN) in accordance with the manufacturer’s instructions. Purified DNAs were stored at −20ºC until use in the following PCR experiment.

### PCR and DNA sequencing

Six sets of PCR primers with tail sequences for Illumina MiSeq sequencing were used in this study (detailed in Table 1). The 18S ribosomal RNA gene fragments in the corresponding regions were amplified using nematode-specific (regions 1–4) and universal primers (regions U1 and U2), respectively (Fig 1B). The PCR reaction mixture (20 μL) contained 10 μL of 2 × Buffer for KOD FX Neo, 4 μL of 2 mM dNTPs, 0.4 units of KOD FX Neo DNA polymerase (Toyobo, Tokyo, Japan), 2 μL of template DNA, and 0.3 μM each of the forward and reverse primers. Amplification was initiated with denaturation at 94°C for 2 min followed by 30 cycles of denaturation at 94°C for 10 s, annealing at 55°C for 30 s, and extension at 68°C for 60 s. The amplified PCR products were purified with 0.8 volumes of AMPure XP beads in accordance with the manufacturer’s instructions and eluted with 10 mM Tris-HCl (pH 8.5). Index PCR was performed in a thermocycler for eight cycles using a Nextera XT Index Kit v2 (Illumina, San Diego, CA, USA) in accordance with the manufacturer’s instructions. The amplified libraries were purified with 1.12 volumes of AMPure XP beads and eluted with 10 mM Tris-HCl (pH 8.5). Equal amounts of the libraries were pooled and quantified using a Qubit dsDNA HS Assay Kit (Thermo Fisher Scientific, Waltham, MA, USA). Each 300 bp end of the pooled library was sequenced using a MiSeq Reagent Kit v3 (600 cycles; Illumina) on a MiSeq instrument (Illumina). The sequences were deposited in the DDBJ Sequence Read Archive database under accession number DRA012120 with BioProject ID PRJDB11694 and BioSample IDs SAMD00325389 to SAMD00325398. Details of the registered data are shown in S1 Table.

**Table 1:** Polymerase chain reaction (PCR) primers used for amplifying the small subunit ribosomal RNA (SSU) gene regions.

SSU gene region^a^	Primer	Nucleotide sequence (5′-to-3′)^b^	Amplicon size (bp)^c^
**Region 1**	SSU18A-4F3_MiseqF	tcgtcggcagcgtcagatgtgtataagagacagGCTTRTCTCAAAGATTAAGCCATGCATG	388
	SSU_R22_MiseqR	gtctcgtgggctcggagatgtgtataagagacagGCCTGCTGCCTTCCTTGGA	
**Region 2**	SSUconsF1_MiseqF	tcgtcggcagcgtcagatgtgtataagagacagAGCAGCCGCGGTAATTCCAGCTC	384
	SSU26Rplus4_MiseqR	gtctcgtgggctcggagatgtgtataagagacagAAGACATTCTTGGCAAATGCTTTCG	
**Region 3**	Nem_18SR_ExtF_MiseqF	tcgtcggcagcgtcagatgtgtataagagacagGTTCGAAGGCGATYAGATACCGCC	337
	SSU_R23plus7_MiseqR	gtctcgtgggctcggagatgtgtataagagacagTCGYTCGTTATCGGAATWAACCAGAC	
**Region 4**	NF1_MiseqF	tcgtcggcagcgtcagatgtgtataagagacagGGTGGTGCATGGCCGTTCTTAGTT	368
	18Sr2b_ExtR_MiseqR	gtctcgtgggctcggagatgtgtataagagacagGGTGTGTACAAAKSGCAGGGACGTA	
**Region U1**	F574-18S_V4_MiseqF	tcgtcggcagcgtcagatgtgtataagagacagGCGGTAATTCCAGCTCCAA	368
	R952-18S_V4_MiseqR	gtctcgtgggctcggagatgtgtataagagacagTTGGCAAATGCTTTCGC	
**Region U2**	F1183-18S_V7-V8_MiseqF	tcgtcggcagcgtcagatgtgtataagagacagAATTTGACTCAACACGGG	446
	R1631a-18S_V7-V8_MiseqR	gtctcgtgggctcggagatgtgtataagagacagTACAAAGGGCAGGGACG	

^a^ These regions are detailed by Kenmotsu et al. [29, 30] and in Fig 1B.

^b^ Nucleotide sequences in lowercase letters indicate the tail sequence required for the attachment of P5 and P7 adaptors by the index PCR for Illumina sequencing. Details of the nematode-specific primers for four SSU regions (regions 1–4) were previously described by Kenmotsu et al. [29, 30]. Two universal eukaryotic primers for SSU regions U1 and U2 were modified from F-574 and R-952, and F-1183 and R-1631a, respectively, which appeared in the paper by Hadziavdic et al. [32].

^c^ The predicted length of amplicon without tail sequences generated from *C*. *elegans* genomic DNA is indicated in Fig 1B.

### Identification of regional nematode sequence variants

For the experiment 1, the sequence reads from 12 amplicons were obtained with primer sets for six SSU regions and DNAs from nematodes and whole copse soils. For the experiment 2, the sequence reads from eight amplicons were obtained with the universal primers for region U2 and eight soil DNA samples. All sequence data were independently imported into QIIME2 version 2020.2 (https://qiime2.org) [39] (S1 Table). The primer sequences were removed by Cutadapt plugin (version 3.1) with the default parameters [40]. The forward and reverse reads were joined, denoized, and chimera-checked using the dada2 plugin [41]. We utilized the trimming parameters (—p-trunc-len-f and—p-trunc-len-r) of 220, 220 (region 1); 225, 200 (region 2); 220, 220 (region 3); 220, 220 (region 4); 230, 230 (region U1); and 229, 220 (region U2), respectively, and utilized the default parameters for the other options. To perform the reference-based detection and removal of the chimeric sequences, we further processed the resultant SVs from dada2 with vsearch uchime ref command with a minimum score option of—minh 0.5 using VSEARCH (version 2.13.3) [42] and the 18S rRNA reference from the SILVA database, version 132, with a 99% clustering threshold (https://www.arb-silva.de/download/archive/) [43]. The taxonomy of the SVs was assigned using a feature-classifier plugin that was trained with the 99% clustered 18S rRNA references in the SILVA database.

The taxonomic ranks of the nematode-derived SVs were curated by manual inspection. A few questionable SVs found by BLASTN search were removed from the nematode-derived SVs identified by the SILVA database, and the resultant SVs were used as nematode-derived SVs for further taxonomic analyses. The resultant nematode-derived SVs in each region (i.e., regional nematode SVs) were named according to their region and SV number; for instance, SV 1 in regions 1 and U2 were named as “R1_SV_1” and “U2_SV_1,” respectively. The SV numbers (e.g., 75 of R1_SV_75) were counted along with the abundance of sequence reads of SV, where a lower number represents the SV containing a larger number of reads.

The frequency of the SVs in each sample were converted based on their relative abundance of reads and visualized using phyloseq package in R (version 4.0.2) [44]. The phylum-level compositions of the SV-derived reads (frequency >1% of the average) were shown as histograms for each sample. The relative abundance of each regional nematode SV was determined as the percentage of the reads of the SV in the total sequence reads of all regional nematode SVs.

### Taxonomic and phylogenetic analyses of regional nematode SVs

The taxonomic analysis of regional nematode SVs was performed in two steps as previously described [30]. First, the taxonomic ranks of the regional nematode SVs were determined basing from the SILVA database [43]. Next, regional nematode SVs were assigned to an order, family, and genus in accordance with their closest species match via the BLASTN search against the non-redundant nucleotide sequence database of the National Center for Biotechnology Information website (https://www.ncbi.nlm.nih.gov) in January 2021. The resulting entries with the smallest e-values, which represent hit significance in Expect value, were only used to identify the closest species to the queried nematode SVs (i.e., matches without genus data, such as environmental samples, were omitted even if they had the smallest e-values). Six phylogenetic trees of the regional nematode SVs were constructed using the corresponding regional sequences of *Halobiotus crispae* (phylum Tardigrada) as an outgroup as described previously [29, 30]. In brief, the nucleotide sequences were aligned, and phylogenetic trees were constructed using the BOOTSTRAP N-J TREE algorithm (bootstrap: 1000 replicates) with the ClustalX (version 2.1) package (http://www.clustal.org/clustal2/) in the Genetyx-MAC software (version 19, Genetyx Co.) [45]. The resultant tree files were used to draw the cladograms using the Genetyx-Tree software (version 2.2.6, Genetyx Co.). In addition, the ATGC software (version 6, Genetyx Co., Tokyo, Japan) was used to identify the copse-derived regional nematode SVs from region U2 with high sequence similarities with a 99% match and minimum matching number of 100 bp, followed by manual inspection.

### Diversity and ecophysiological analyses

Beta diversity analyses were performed using phyloseq package [44]. For the analysis of 12 samples from the copse-derived nematode and soil DNAs, the nematode-derived reads (over five reads at least in one region) in each order and feeding type were used. Then, non-metric multidimensional scaling (NMDS) was conducted with the Bray–Curtis distance matrices using the ordinate and plot ordination functions in R phyloseq to evaluate the differences in order and feeding type among six regions and two copse-derived template DNAs. For beta diversity analysis of the field-derived samples, the read abundances of nematode-derived SVs were used for principal coordinate analysis (PCoA) based on weighted unifrac distances. Feeding types of the regional nematode SVs were assigned according to their closest genus as identified by BLASTN searches based on the reference by Yeates et al. [46] and the Nematode Ecophysiological Parameter Search at the Nemaplex homepage of UC Davis, USA (http://nemaplex.ucdavis.edu/Ecology/EcophysiologyParms/EcoParameterMenu.html) in January 2021 [47]. Animal parasites were referred to previous publications [48–53]. The colonizer–persister (cp) values of nematode families were classified as previously described by Bongers [54, 55] and the Nematode Ecophysiological Parameter Search [47]. Maturity indices were calculated as the weighted mean of the individual cp values as previously described by Bonger [55]:

$$Maturity\;Index=\sum_{i=1}^{n}v\left(i\right)\times f\left(i\right),$$

where *v*(*i*) is the cp-value of family *i* and *f*(*i*) is the frequency of reads from the family compared to those from all families without reads derived from unassigned families (NA) in a sample.

## Results

### Sequence variants identified by high-throughput amplicon sequencing using six SSU primer sets and nematode and whole soil DNAs

In the experiment 1, we performed high-throughput amplicon sequencing of copse soil using six primer sets for amplifying the SSU gene: the nematode-specific primers for regions 1–4 and the universal eukaryotic primers for regions U1 and U2, respectively (Fig 1B). In addition, template DNAs were prepared from nematodes isolates from 40 g of copse soil (nematode DNA) and 10 g copse soil (soil DNA). Hundreds of SVs were identified by Illumina MiSeq-assisted amplicon sequencing (Table 2): the largest (883) and smallest (338) numbers of SVs from the nematode and soil DNAs were found in regions 1 and 3, respectively. Approximately twofold larger numbers of SVs (250–739 SVs) were obtained from the soil DNA than those from the nematode DNA (128–283 SVs). Results showed that 26–79 (12.0%–27.9% in total SVs) and 25–41 (3.9%–16.4%) SVs derived from the phylum Nematoda were identified in the nematode and soil DNAs, respectively. The smallest fractions of nematode-derived SVs were found in region 1 in both template DNAs (12.0% in nematode DNA and 3.9% in soil DNA), and the highest content of nematode-derived SVs was 27.9% in region U2 in nematode DNA and 16.4% in region 3 in soil DNA, respectively.

**Table 2:** Numbers of total and nematode-derived SVs from the copse-derived nematode and soil DNAs in each SSU region.

**Nematode and soil DNAs** ^a^						
SSU region	Region 1	Region 2	Region 3	Region 4	Region U1	Region U2
Total SVs	883	547	338	721	494	678
Nematode-derived SVs	43^b^ (4.9%^c^)	60 (11.0%)	50 (14.8%)	57 (7.9%)	52 (10.5%)	90 (13.3%)
**Nematode DNA** ^a^						
SSU region	Region 1	Region 2	Region 3	Region 4	Region U1	Region U2
Total SVs	266	212	128	263	193	283
Nematode-derived SVs	32^b^ (12.0%^c^)	47 (22.2%)	26 (20.3%)	44 (16.7%)	39 (20.2%)	79 (27.9%)
**Soil DNA** ^a^						
SSU region	Region 1	Region 2	Region 3	Region 4	Region U1	Region U2
Total SVs	739	460	250	556	368	472
Nematode-derived SVs	29^b^ (3.9%^c^)	34 (7.4%)	41 (16.4%)	35 (6.3%)	25 (6.8%)	31 (6.6%)

^a^ Sequence variant-derived template DNAs. Nematode genomic DNAs were prepared from complex nematodes isolated from 40 g of the copse soil, and soil DNA was purified from 10 g aliquot of the soil.

^b^ Numbers of nematode-derived SVs in each region (i.e., regional nematode SVs) derived from each template DNA are shown. The regional nematode SVs are determined based on the taxonomic ranks in the SILVA database and the BLASTN search. Note: Numbers of SVs in the nematode and soil DNAs are not identical to the added numbers of those in the nematode and soil DNAs because of the SVs commonly identified in both DNAs.

^c^ Percentage of the number of nematode-derived SVs in total number of SVs derived from each template DNA.

Numbers of regional nematode SVs identified from the nematode and/or soil DNAs were slightly varied among the SSU regions. Comparable numbers (43–60) of regional nematode SVs were detected in regions 1–4 and U1; however, a significantly large number (90) of nematode SVs was found in region U2 (Table 2). The comparable or slightly larger numbers of regional nematode SVs were identified in the nematode DNA by comparing with those in the soil DNA in all regions, except for region 3. The resultant regional nematode SVs were assigned to their derived template DNAs (Table 3). Less than 50% (22.2%–41.8%) of nematode SVs were commonly detected in both template DNAs. Larger numbers of nematode SVs were detected in the nematode DNAs alone than those in the soil DNA alone in the five regions above. However, half of the nematode SVs in region 3 were only identified in the soil DNA. Moreover, >50% of the nematode SVs in regions U1 and U2 were derived from nematode genomic DNA (Table 3). We further investigated the homologies of nucleotide sequences of 90 regional nematode SVs in region U2 and identified 11 clusters of SVs with high sequence similarities (S2 Table). Most of these SVs in each cluster were derived from the nematode DNA but not from the soil DNA and contained differing one nucleotide in their sequences, suggesting polymorphic alleles of the SSU gene as found in previous studies [28, 29].

**Table 3:** Numbers of regional nematode SVs derived from nematode genomic and whole soil DNAs.

SSU region	Region 1	Region 2	Region 3	Region 4	Region U1	Region U2
Total	43^a^	60	50	57	52	90
Common	18(41.8%)^b^	21(35.0%)	17(34.0%)	22(38.6%)	12(23.1%)	20(22.2%)
Nematode alone	14(32.6%)	26(43.3%)	9(18.0%)	22(38.6%)	27(51.9%)	59(65.6%)
Soil alone	11(25.6%)	13(21.7%)	24(48.0%)	13(22.8%)	13(25.0%)	11(12.2%)

^a^Total numbers of regional nematode SVs are shown in the “total” column for each region.

^b^Numbers of regional nematode SVs exclusively detected in nematode genomic DNA, soil DNA, and both DNAs are indicated in the “nematode alone,” “soil alone,” and “common” columns with their percentages in total regional nematode SVs in parentheses, respectively.

### Taxonomic analyses of total and regional nematode SVs from copse-derived amplicons

**Fig 3:**
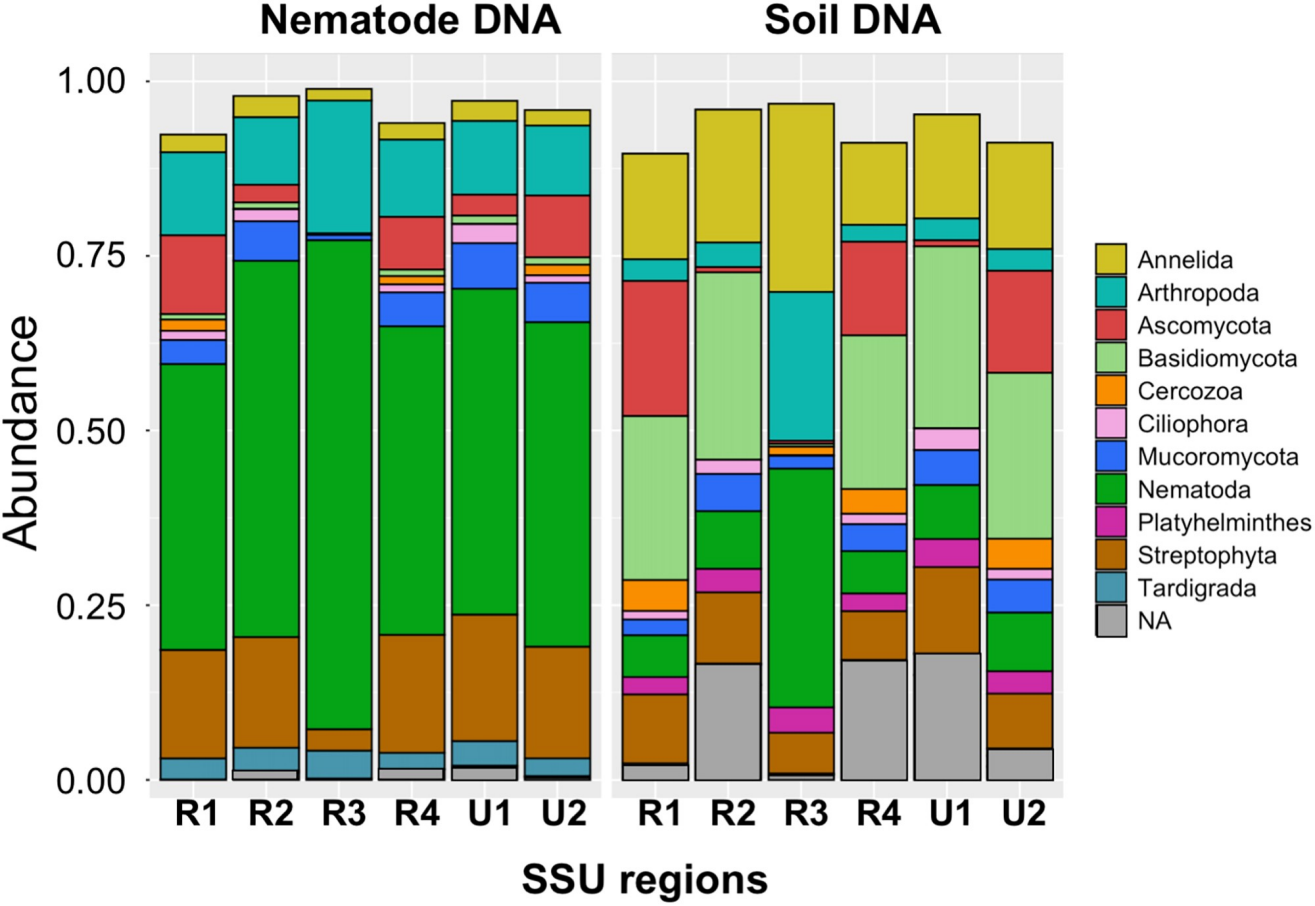
Relative read abundances and phylum of sequence variants (SVs) in each region identified by high-throughput sequencing of the copse-derived amplicons. Relative read abundance and phylum of the SVs obtained from the SSU gene regions 1 (R1), 2 (R2), 3 (R3), and 4 (R4) amplified by the nematode-specific primers, and regions U1 and U2 amplified by the universal primers from the copse-derived nematode DNA (left panel) and soil DNA (right panel) as templates. Boxes in the histogram indicate the read abundances; phyla are indicated by color. Taxonomic classification of SVs was based on the SILVA database, and phyla with less than 1% of relative abundance are omitted. NA: Not assigned.

The relative abundances of phylum with >1% abundance of nematode-derived sequence reads were indicated in each region (Fig 3). In the amplicons from nematode genomic DNA, the largest fractions (i.e., approximately 50%) of total reads were derived from the phylum Nematoda, followed by the Arthropoda (insects)-, Streptophyta (plants)-, and Ascomycota (fungi)-derived reads as major fractions in the six SSU regions (left panel in Fig 3). Conversely, the nematode-derived fractions occupied less than 10% in soil DNA-derived amplicons, except for 34% in region 3 (right panel in Fig 3). In addition, the fractions of reads derived from the phyla Annelida (ringed or segmented worms), Ascomycota, Basidiomycota (filamentous fungi), and Streptophyta were dominantly found. Similar compositions of the relative abundance of phylum-derived reads were found in two regional groups: regions 1, 4, and U2 and regions 2 and U1. The significant fractions of the phylum Ascomycota (red boxes) were detected in both DNAs in the former group but not in the latter two regions and region 3. The proportion of phylum-derived reads in region 3 was distinct from those in the five other regions: significantly large and small fractions of nematode- and fungi-derived reads as well as abundant Arthropoda-derived reads were detected in this region. Thus, the proportion of phylum-derived reads in region U2 was comparable to that in region 4, which was suggested a suitable region for amplicon sequencing with nematode DNAs [29, 30].

**Fig 4:**
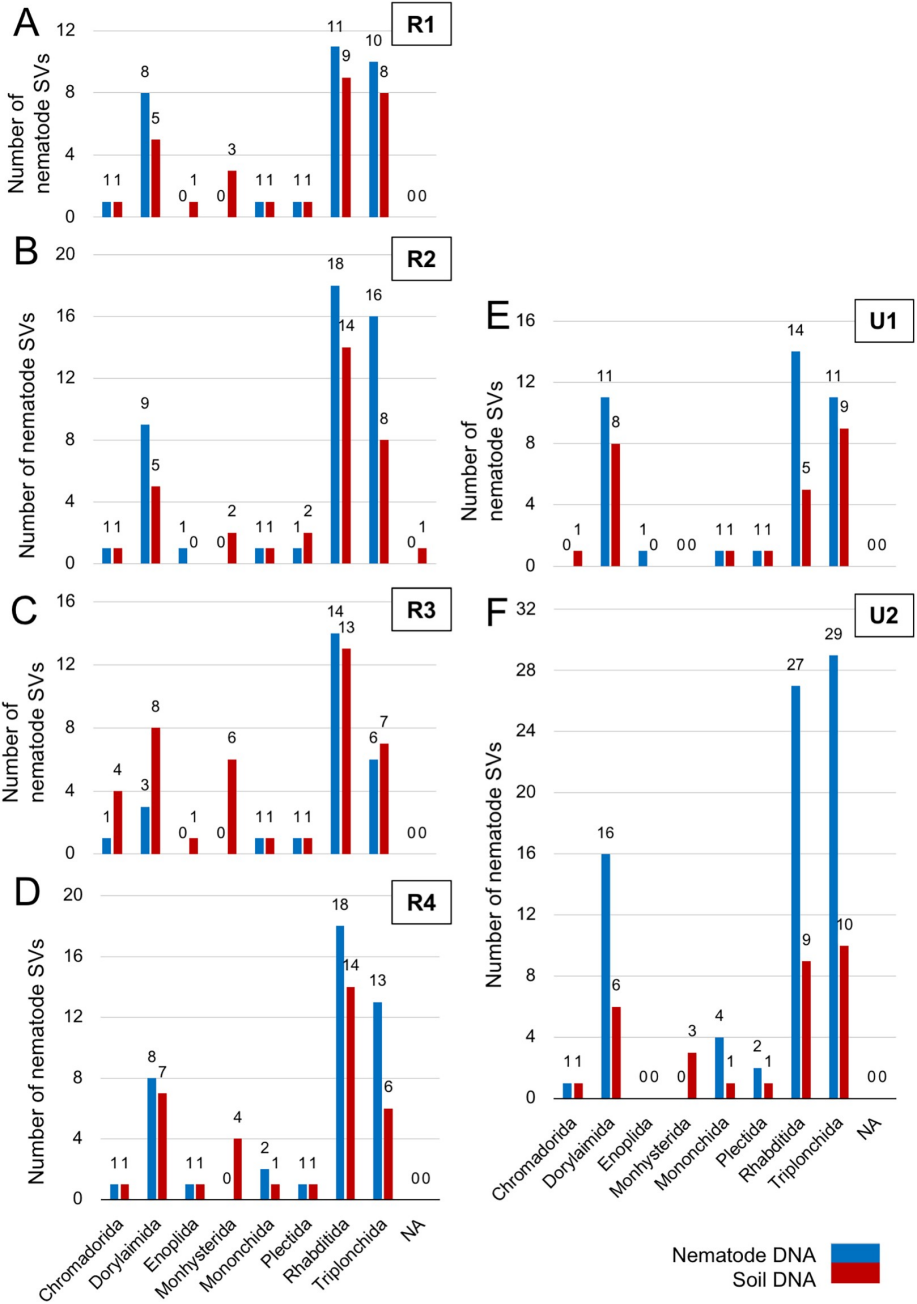
Numbers of regional nematode SVs from six SSU regions in each order. The number of regional nematode SVs identified from the nematode genomic DNA (blue bars) and soil DNA (red bars) from the copse soils is indicated in each order by bar chart for regions 1–4, U1, and U2 (A–F), respectively. NA: not assigned to a single order (i.e., assigned to multiple orders). Target SSU regions are indicated at the top right side of the bar charts.

**Fig 5:**
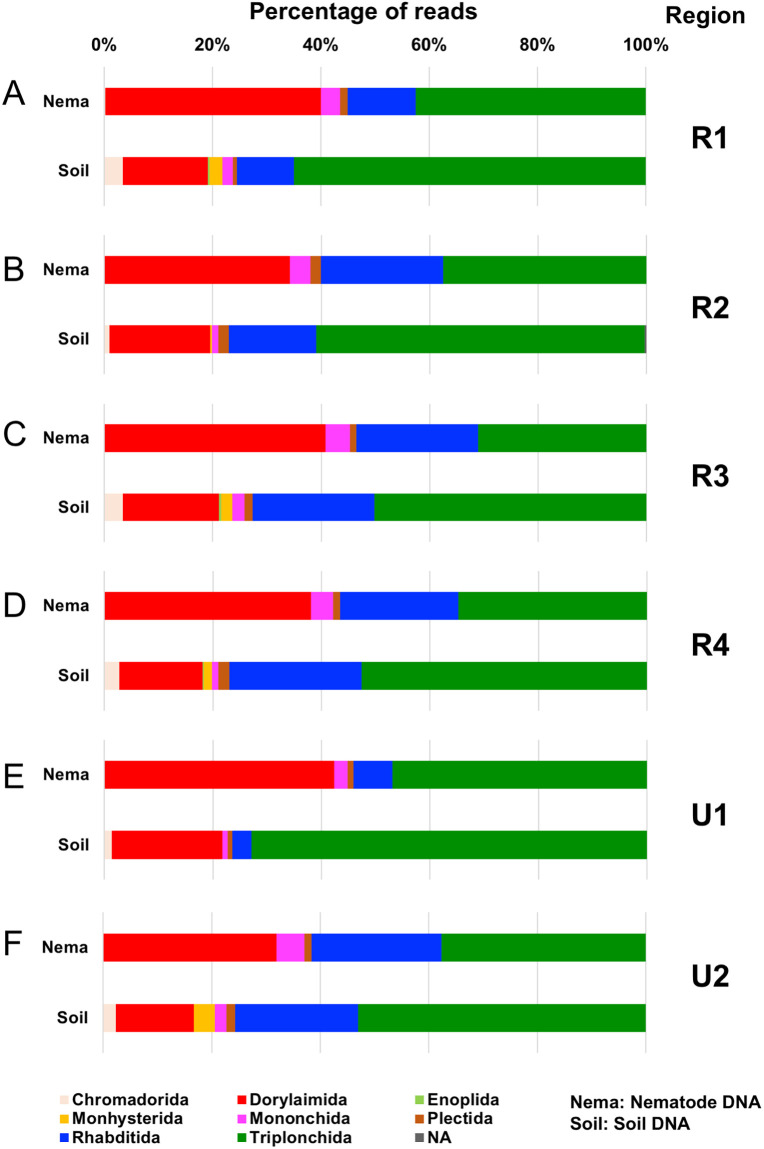
Relative abundance of nematode-derived sequence reads at the order level in each region. The percentage of the abundance of sequence reads in each nematode order obtained from regions 1 (A), 2 (B), 3 (C), 4 (D), U1 (E), and U2 (F) is indicated by colored horizontal histograms in each template DNA: copse-derived nematode genomic DNA (Nema) and soil DNA (Soil). The colors corresponding to orders are indicated at the bottom of (F). Target SSU regions are indicated at the right side of the corresponding horizontal bar charts.

**Fig 6:**
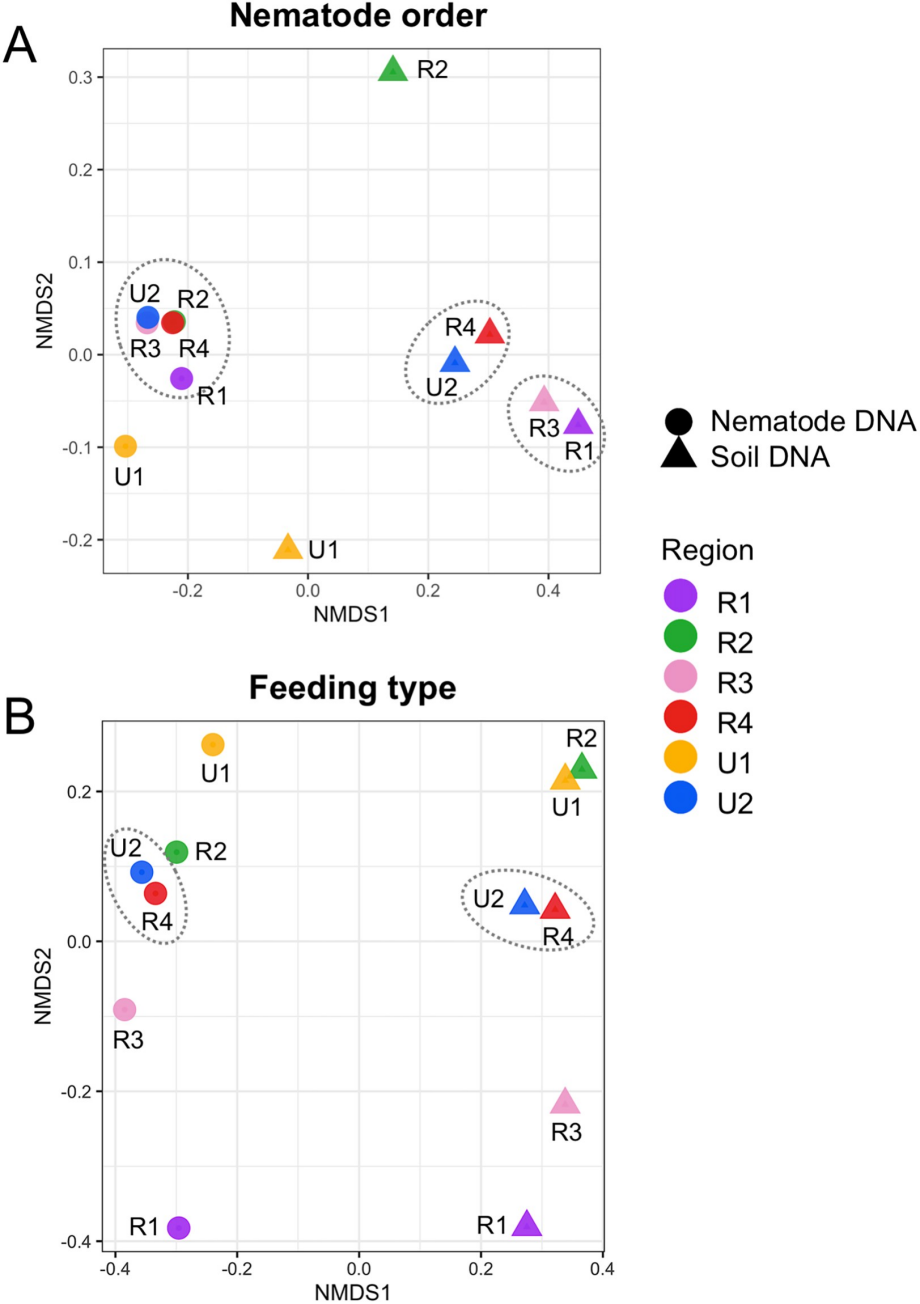
Beta diversity plots of 12 samples derived from two template DNAs and six SSU regions based on the relative abundance of nematode-derived reads in each order and feeding type. The beta diversity of the sample was calculated based on the relative abundances of nematode-derived reads in each order (A) and feeding type (B) using non-metric multidimensional scaling (NMDS) with the Bray–Curtis distance matrices in the corresponding SSU region and template DNA indicated in the right of figures. The nematode-derived reads in region 1 (R1, purple), 2 (R2, green), 3 (R3, pink), 4 (R4, red), U1 (orange), and U2 (blue) are derived from the nematode genomic DNA (circles) and soil DNA (triangles). The target SSU region of each sample is indicated by the corresponding symbol. The samples with close relations described in the text are surrounded by broken circles.

The taxa of regional nematode SVs identified in six regions were assigned by both SILVA-derived taxonomic data and the BLASTN search (S3–S8 Tables). The numbers of regional nematode SVs in orders are shown by histograms in each region (Fig 4). The resultant nematode SVs were classified into eight orders (i.e., Chromadorida, Dorylaimida, Enoplida, Monhysterida, Mononchida, Plectida, Rhabditida, and Triplonchida) and unclassified SVs (NA), and the majority of nematode SVs were derived from orders Rhabditida, Triplonchida, and Dorylaimida. The histograms of nematode SVs in regions 1 (Fig 4A), 2 (Fig 4B), and 4 (Fig 4D) were similar except for region 3 (Fig 4C). The numbers of SVs identified in regions U1 and U2 were comparable to those in these three regions (Fig 4E and 4F), whereas large numbers of nematode SVs were identified from the nematode DNA in region U2. In addition, the Enoplida- and Monhysterida-derived SVs were undetected in regions U2 and U1, respectively. The even or larger numbers of nematode SVs in seven orders were detected in the nematode DNA (blue bars in Fig 4) by comparing with those in the soil DNA (red bars), but the Monhysterida-derived SVs were only found in the soil DNA. In addition, the larger numbers of Chromadorida-, Dorylaimida- and Triplonchida-derived SVs from the soil DNA were identified only in region 3 than those from the nematode DNA (Fig 4C). The relative abundance of sequence reads from each region and template DNA was shown in each nematode order (Fig 5). The major fractions of reads were derived from orders Triplonchida, Dorylamida, and Rhabditida, and the Triplonchida- and Dorylamida-derived reads occupied >70% of the total reads from the nematode and soil DNAs (red and green boxes in Fig 5). Distinct compositions of order-derived reads were markedly found between the two template DNAs (“Nema” and “Soil” columns in Fig 5). The relative abundances of Dorylamida-derived reads significantly increased and those of Triplonchida-derived reads decreased in the nematode genomic DNA across the regions and *vice versa* in soil DNAs. The compositions of order-derived reads in the two template DNAs were similar in all regions, except for regions 1 and U1 (Fig 5B–5D and 5F), whose fractions of Rhabditida-derived reads were apparently small (Fig 5A and 5E). The differences in compositions of the order-derived reads from the six regions and two DNAs were further investigated by beta diversity analysis (Fig 6A), and the resultant NMDS plot of the Bray–Curtis dissimilarity indicated close relations of the nematode DNA-derived reads from all regions, except for region U1 (circles in Fig 6A). The region 4- and U2-derived reads (red and blue triangles in Fig 6A) and region 1- and 3-derived reads (purple and pink triangles) were also closely related. However, the soil DNA-derived reads from regions 2 (green triangle) and U1 (yellow triangle) were clearly separated from those from the four other regions.

### Phylogenetic and ecophysiological analyses of copse-derived regional nematode SVs

We further subjected the regional nematode SVs to phylogenetic and ecophysiological analyses. First, a phylogenetic tree of the regional nematode SVs was prepared in each region integrating with their orders, relative abundances of reads from the template DNAs, and predicted feeding types (S1–S6 Figs). The phylogenetic trees contained three major clusters of SVs derived from orders Triplonchida, Dorylaimida, and Rhabditida as described in the former section. Major nematode SVs with abundant reads were commonly found in amplicons from both nematode (“Nema” columns in S1–S6 Figs) and soil DNAs (“Soil” columns). However, significant numbers of regional nematode SVs were found only in either nematode DNA- or soil DNA-derived amplicons (Table 3). For instance, the Monhysterida-derived SVs were only found in the soil DNA-derived amplicons in all regions, except for region U1 (S1–S4 and S6 Figs), and all of the animal parasitic Rhabditida-derived SVs, except for R4_SV_12, were detected only in the amplicons from the nematode DNA. In addition, the phylogenetic tree with the largest number of region U2-derived SVs contained two split clusters of the Rhabditida- and Triplonchida-derived SVs, which were mostly derived from the nematode genomic DNA (S6 Fig). One of the Rhabditida-derived clusters contains animal parasite-derived SVs, and the other is composed of plant and bacteria feeder-derived SVs. The Triplonchida-derived SVs were separated into clusters of bacteria feeders and of plant and fungus feeders, respectively.

Second, the feeding types of the regional nematode SVs were predicted, and many of these SVs were assigned to bacteria, fungus, and plant feeders, but only one or a few SVs were derived from omnivores, predators, and eukaryotic feeders (S7 Fig). The numbers of SVs derived from the six feeding types were largely comparable among the regions, except for the animal parasite-derived SVs and the region U2-derived SVs. Except for R4_SV_12 in region 4, the animal parasite-derived SVs were exclusively derived from the nematode DNA (S7D Fig), and approximately twofold larger numbers of plant feeder- and animal parasite-derived SVs (32 and 13 SVs) were identified from the nematode DNA in region U2 than those from the soil DNA (S7F Fig). The relative abundances of reads in each feeding type were also determined in the six SSU regions and template DNAs (S8 Fig). Despite the similar horizontal bar charts among the regions, the proportions of read abundances from the two template DNAs in the feeding type were distinct. In specific, the eukaryote feeder-derived reads and approximately twofold increased bacteria feeder-derived reads were observed most exclusively in the soil DNA, but the animal parasite-derived reads were only detected in the nematode DNA (S8 Fig). We further conducted beta diversity analysis using the read abundance in feeding type. The close relation of region 4- and U2-derived reads in both template DNAs was indicated by the resultant NMDS plot (Fig 6B). The feeding type’s compositions in regions 1 and 3 were distantly related in the two DNAs, unlike the beta diversity analysis with their order’s compositions (Fig 6A and 6B).

Third, the regional nematode SVs were assigned to 27 nematode families, but this number is likely underestimated because of several SVs assigned to multiple families, which are classified into “not assigned (NA)” (S9 Table). The comparable numbers (12–18) of nematode families were detected in the regions. Twenty-four and nineteen families were detected from the soil and nematode DNAs, respectively, and eight (i.e., Alaimidae, Anguinidae, Aporcelaimidae, Ecphyadophoridae, Monhysteridae, Meloidogynidae, Nygolaimidae, and Qudsianematidae) and three (Thelastomatidae, Travassosinematidae, and Trischistomatidae) families were undetected in the nematode DNA- and soil DNA-derived SVs, respectively (S9 Table). The relative read abundances in each family are shown along the corresponding cp values in the six regions (S9A–S9F Fig). The cp values (1–5) have been used as indicators for the life strategy characteristics of nematodes [54, 55]: nematodes with low cp values have a short generation time and produce many small eggs, resulting in an explosive population growth under food-rich conditions, whereas those with high cp values have a long life span and low production rate, and are thus highly sensitive to environmental disturbances. The profiles of relative abundances in families were similar across the regions, and the sequence reads derived from families Prismatolaimidae (cp-3), Trichodoridae (cp-4), and Belondiridae (cp-5) were markedly abundant. In addition, the relative abundances of reads from nematode families with high cp values increased in the nematode DNA-derived amplicons (blue bars in S9 Fig) but not in the amplicons from soil DNA (orange bars).

Finally, we determined the maturity indices for each region-derived families based on their cp values and frequencies in the population (Table 4). The maturity indices for the nematode families identified from the copse-derived nematode DNA and whole soil DNA were 3.71–3.94 and 3.11–3.50 in the six regions, respectively.

**Table 4:** Maturity indices calculated by the families identified from the copse-derived nematode DNA and soil DNA in six SSU regions.

SSU regions	Region 1	Region 2	Region 3	Region 4	Region U1	Region U2
Nematode DNA	3.90	3.71	3.89	3.76	3.94	3.71
Soil DNA	3.32	3.28	3.20	3.11	3.50	3.13

Maturity indices were calculated as described in the Materials and methods section based on the method described by Bongers [55].

### Taxonomic and ecophysiological analyses of soil nematodes in agricultural field by amplicon sequencing using the universal primers and soil DNAs

Comparable results were obtained from the analyses using universal primers and soil DNA and those using nematode-specific primers and nematode DNA as described in the previous section. Therefore, in the experiment 2, we applied this method to the taxonomic analyses of soil nematode community in a sweet potato-cultivated agricultural field using the universal primers for region U2 and whole soil DNAs (Fig 2A). In this experiment, we isolated eight soil samples from two sites in the field, where the plants at site 1 were dominantly growing than those at site 2, and four samples at each site were from the surface (0–10 cm in depth) and deep (20–30 cm) layers with (plants) and without (control) plants (Fig 2B–2D). Larger numbers of total and nematode-derived SVs were identified from the amplicons derived from site 1 than those from site 2 (Table 5). Greater numbers (61 and 53) of nematode-derived SVs were detected from the amplicons derived from the plant-associated soils at site 1 than those (34 and 37 SVs) from the control soils without plants, but a different phenomenon was observed at site 2.

**Table 5:** Numbers of total and nematode-derived SVs from eight soil samples at sites 1 and 2.

**Site 1**				
	Control^a^		Plants^a^	
	0–10 cm^b^	20–30 cm^b^	0–10 cm	20–30 cm
Total SVs	943	1069	983	1276
Nematode-derived SVs	34^c^ (3.6%^d^)	37 (3.5%)	61 (6.2%)	53 (4.2%)
**Site 2**				
	Control		Plants	
	0–10 cm	20–30 cm	0–10 cm	20–30 cm
Total SVs	716	884	622	920
Nematode-derived SVs	19 (2.7%)	34 (3.8%)	22 (3.5%)	20 (2.2%)

^a^ Soil samples isolated from the sampling point without (control) and with (plants) a growing sweet potato at each site in the agricultural field.

^b^ Soil samples isolated from the layer of 0–10 cm and 20–30 cm in depth.

^c^ Numbers of nematode-derived SVs are based on the taxonomic ranks in the SILVA database and the BLASN search.

^d^ Percentage of nematode-derived SVs in the total number of SVs.

**Fig 7:**
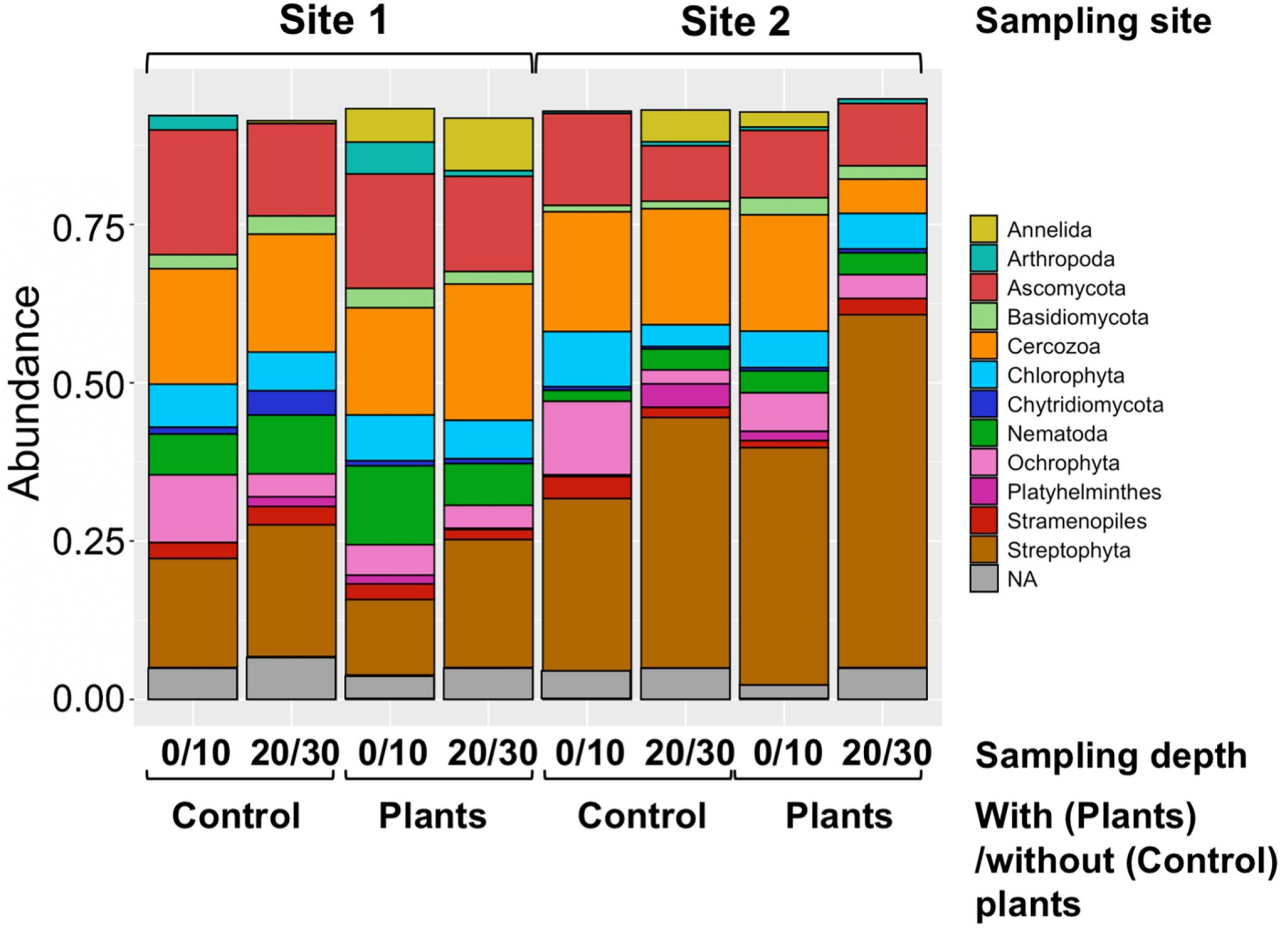
Relative read abundances and phylum of SVs obtained from high-throughput amplicon sequencing using the agricultural field-derived soil DNAs. Relative read abundance and phylum of SVs obtained from the amplicon sequencing of region U2 of the SSU gene using soil DNAs derived from the surface (0/10) and deep (20/30) layers at the sampling points without (control) and with (plants) plants at sites 1 and 2, respectively, as shown at the top and bottom of the histograms. Boxes in the histogram indicate the read abundances; phylum is indicated by color as shown in the right legend box. Taxonomic classification of SVs was based on the SILVA database. Phyla with less than 1% of relative abundance were omitted. NA: Not assigned.

The relative abundance of sequence reads derived from each soil sample is indicated at the phylum-level (Fig 7). The sequence reads derived from the phyla Streptophyta (plants), Ascomycota (fungi), and Cercozoa (single-celled eukaryotes) were abundantly found, and the nematode-derived reads occupied as minor fractions: 5%–12% and <4% of the total reads derived from sites 1 and 2, respectively.

**Fig 8:**
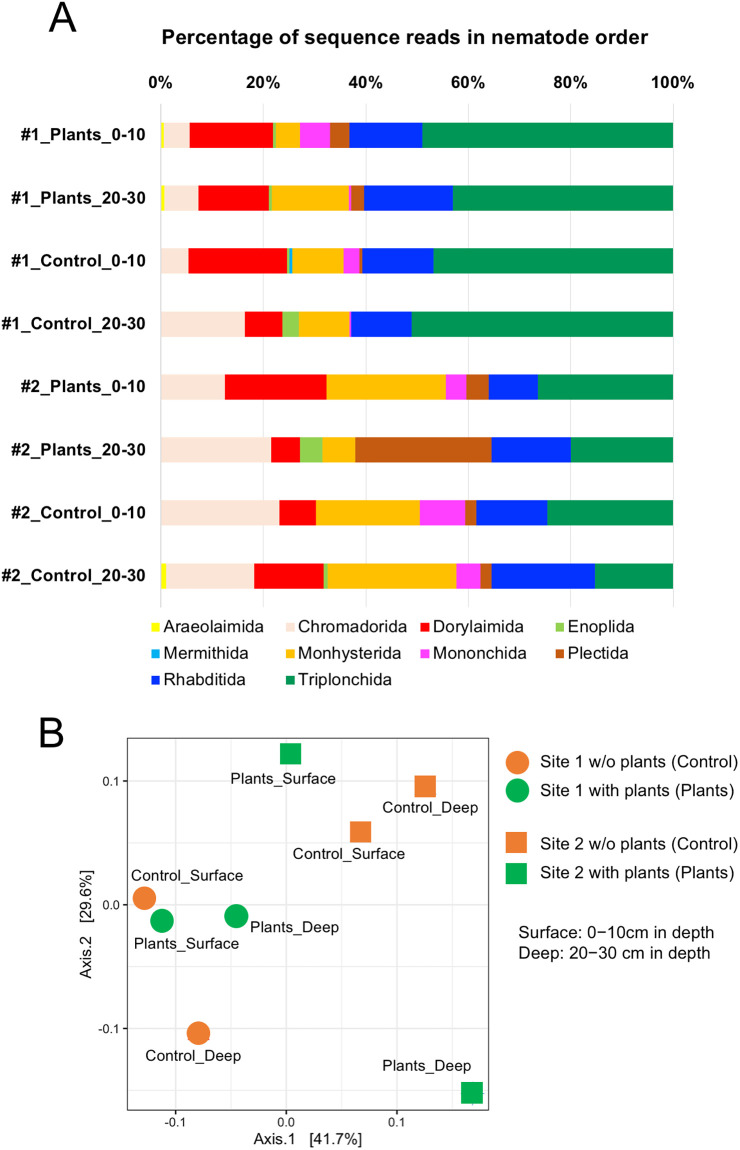
Relative abundances of nematode-derived reads from the field-derived samples in each order and their beta diversity plots. The percentage of the abundance of sequence reads derived from the soil samples in each order is shown by colored horizontal bar charts (A). Soil samples were isolated from the surface (0–10) and deep (20–30) layers of the sampling points without (control) and with (plants) plants at sites 1 (#1) and 2 (#2), respectively, as shown in the left of the corresponding histogram. Colors corresponding to orders are indicated at the bottom of figure. The principal coordinate analysis (PCoA) plot with the weighted unifrac distances of eight samples derived from the surface and deep layer at the point without (orange-colored circles and squares) and with (green-colored circles and squares) plants from site 1 (circles) and 2 (squares) in the agricultural field (B).

The taxa of 101 nematode-derived SVs identified from eight soil samples were determined (S10 Table), and the numbers of SVs identified from sites 1 and 2 are shown in each order by horizontal bar charts (S10 Fig). These nematode-derived SVs were assigned to 10 orders; the majorities of these SVs were derived from five orders (Rhabditida, Monhysterida, Dorylamida, Triplonchida, and Chromadorida), and the Araeolaimida- and Mermithida-derived SVs were very rare. The relative abundances of the Triplonchida-, Chromadoria- and Monhysterida-derived reads in total reads were apparently distinguishable between the samples from sites 1 and 2 (Fig 8A). The fractions of the Triplonchida-derived reads occupied approximately 40%–50% of the total reads from the site 1 samples, whereas those of the Chromadorida- and Monhysterida-derived reads increased in the site 2 samples. The Plectida-derived reads, mainly from U2_SV_101, were significantly abundant only in the plant-associated soils from the deep layer at site 2 (#2_Plants_20–30 in Fig 8A), suggesting close relationship between the Plectida species and plants growth and/or plants-associated bacteria because many of the Plectida-derived nematodes are bacterivores. However, this phenomenon was difficult to describe because the inconsistent observation was found at the comparable sample at site 1 (#1_Plants_20–30 in Fig 8A). In addition, the increased fractions of the order Mononchida-derived reads were commonly detected in the samples prepared from the surface layers by comparing with those in the deep layer-derived samples (Fig 8A). The beta diversity analysis was performed to assess the differences in compositions of nematode SVs in the eight soil samples, and the resultant PCoA plot with weighted unifrac distances indicated the distantly related samples derived from site 1 (symbols in red) and site 2 (blue symbols), except for one sample (#2_Plants_20–30) (Fig 8B). Regarding to the sampling depth, close relation between the samples derived from the surface soils with and without plants at site 1 was found, but this was not consistent with the samples at site 2.

The numbers of nematode-derived SVs and their relative sequence reads in the feeding type are shown in S11 and S12 Figs, respectively. First, these data notably indicated that the bacteria feeder-derived SVs were the majority of the nematode population in the agricultural field because over 60% of reads were assigned to bacteria feeder in all samples (S12 Fig). Second, the fractions of reads from eukaryote feeder-derived SVs (e.g., U2_SV_40, 80, and 179), which are closely related to the genus *Achromadora* (S10 Table), clearly increased in the samples from site 2. In addition, the numbers of nematode-derived SVs and their relative read abundances in each family with cp-value are summarized in S13 and S14 Figs, respectively. Increased numbers of SVs identified from the samples were derived from the families Monhysteridae (cp-2) and Cyatholaimidae (cp-3) followed by Prismatolaimidae (cp-3) and Cephalobidae (cp-2) (S13 Fig). The read abundances from these families were also the majority of nematode-derived reads. In specific, the reads from the SVs (mainly U2_SV_11) were significantly abundant in the site 1-derived samples (S14A Fig). U2_SV_11 was assigned to the genus *Prismatolaimus*, which is a bacteria feeder often found in moist soils [56, 57]. Finally, we determined the maturity indices of 2.51–2.96 for the nematode families identified from the eight samples (Table 6).

**Table 6:** Maturity indices for nematode families from the field-derived soil samples.

	Control		Plants	
	0–10 cm	20–30 cm	0–10 cm	20–30 cm
Site 1	2.96	2.88	2.96	2.79
Site 2	2.75	2.68	2.73	2.51

Maturity indices for the corresponding soil samples from the surface (0–10 cm in depth) and deep layer (20–30 cm) at the sampling point with (plants) and without (control) growing sweet potato at sites 1 and 2 in the field, respectively.

## Discussion

### Testing the universal SSU primers and soil DNAs for DNA amplifications for taxonomic analyses of soil nematodes

In prior taxonomic analyses of nematodes by high-throughput amplicon sequencing, genomic DNAs of complex nematodes and nematode-specific primers have been often used as template DNAs and PCR primers for amplification of the 18S ribosomal RNA (SSU) gene fragments [11, 20, 22, 30, 31]. Recently, whole soil DNAs and/or the universal SSU primers have been applied to DNA metabarcoding for terrestrial nematodes in several studies [12, 21, 34–38, 58]. Considering that PCR primers and template DNAs for the taxonomic analysis of soil nematodes have been poorly evaluated, we have tested four nematode-specific and two universal primer sets as well as nematode and soil DNAs derived from copse soils for the DNA barcoding of nematodes. Although the nematode genomic DNA contains concentrated nematodes separated from other soil eukaryotes, the nematode population in the DNA is largely influenced by nematode isolation methods, such as Baermann funnel method [59], which can preferentially isolate the mobile nematodes in water. Conversely, bulk DNAs from soil samples contain whole eukaryote-derived DNA including nematodes. However, the amount of soils for DNA preparation is limited by DNA purification kits. Upon soil DNA purification, the larger amount of soil samples is ideal for the taxonomic analysis because of the increased content of more complex eukaryotes. In this study, soil DNAs were purified using a DNeasy PowerMax Soil Kit (QIAGEN) because of its highest capacity of up to 10 g soils among commercially available soil DNA purification kits. We also used the eukaryotic universal primers that amplify the 368 and 446 bp fragments spanning the variable regions V4 (region U1 in this study) and V7–V8 (region U2) of the SSU gene [32, 33] (Fig 1B). The V4 [12, 21, 34, 37, 58] and V7–V8 [11, 20, 22, 31, 35, 38, 58] regions were preferentially used for prior DNA metabarcoding of terrestrial nematodes.

We obtained the following observations on two template DNAs used. First, despite the minor difference among the regions, except for regions 3 and U2, slightly larger numbers of nematode-derived SVs were detected in nematode DNAs than in soil DNAs (Table 2). Second, the largest fractions of reads from nematode genomic DNA were derived from the phylum Nematode, but the fractions of the nematode-derived reads from soil DNA were less than 10% of total reads (Figs 3 and 7). Third, <40% of the nematode SVs, including major SVs, were commonly detected in both template DNAs, and the remaining nematode SVs were uniquely found in either nematode DNA or soil DNA (Table 3). Fourth, whereas the numbers of regional nematode SVs (except for the region U2-derived SVs from nematode DNA) in each order were comparable in both template DNAs (Fig 4), the compositions of relative read abundances in order and feeding type were distinguishable by the DNAs (Fig 5 and S8 Fig). This result was also confirmed by the beta diversity analyses based on the read abundances in order and feeding type (Fig 6). Fifth, despite the quarter amount of the soils used for nematode isolation, larger numbers of nematode families were identified from the soil DNA than from the nematode genomic DNAs across the regions (S9 Table). The difference in the amount of soils used may influence the results of this experiment, considering that the nematode genomic and soil DNAs were derived from different amounts of soils (i.e., 40 and 10 g, respectively). The influence of soil amount in amplicon-based taxonomic profiling remains an issue to be investigated in the future. Thus, currently, it is difficult to determine which method using nematode DNA or soil DNA is suitable for taxonomic analysis of soil nematodes because of potential issues such as soil amounts. However, the DNA barcoding detected different nematode-derived SVs (Table 3), and taxonomic variations in the cladograms (S1–S6 Figs) were found in each template DNA, suggesting that both methods with nematode DNA and soil DNA could be used complementary for nematode community analysis each other.

Regarding the PCR primers tested, the following four findings were obtained. First, the relative abundances of the nematode-derived reads were largely comparable at the phylum-level among all regions, except for region 3 (Fig 3). The primers for region 3 preferentially amplified nematode-derived SSU fragments from both template DNAs, and this result is consistent with previous observations in prior studies using complex nematode DNA [30] or individual nematode DNA [29]. In addition, the SSU fragments derived from Ascomycota were differently amplified by the primers for regions 1, 4, and U2 and regions 2, 3, and U1 (Fig 3). Second, the numbers of regional nematode SVs from the nematode and soil DNAs were similar among the six regions, including region U2 with the large numbers of SVs (Table 3), because many of the region U2-derived SVs were likely derived from allelic sequences in complex nematode DNAs (S2 Table). The universal primers for region U2 may tend to amplify polymorphic SSU fragments efficiently when complex nematode DNAs are used. Third, despite the presence of minor differences, the compositions of relative read abundances in order and feeding type were comparable among the regions (Fig 5 and S8 Fig). The majority of the copse-derived nematode SVs was assigned to the orders Triplonchida, Dorylaimida, and Rhabditida (Figs 4 and 5) and were derived from plant and bacteria feeders (S7 and S8 Figs). The relative read abundances in nematode families were also similar among the regions (S9 Fig). Fourth, the beta diversity analyses of relative read abundances in order and feeding type clearly indicated the close relation of region U2 and region 4 (Fig 6), which was suggested as the most suitable nematode-specific target among the four regions by the taxonomic profiling of individual and mixed nematodes [29, 30]. Finally, both primers for regions 4 and U2 identified comparable total numbers of nematode SVs in two template DNAs if the allele-derived SVs are removed (Table 3 and S2 Table), indicating that these primer sets are equally suitable for DNA metabarcoding of soil nematodes.

These observations on template DNAs and the SSU primers suggest that the universal SSU primers for region U2, as the nematode-specific primers for region 4, could be used with soil DNAs for the taxonomic analyses of soil nematodes by high-throughput amplicon sequencing. Prior studies have preferentially used nematode-specific primers and nematode community-derived DNAs for amplicon preparations [11, 20, 22, 30, 31]. However, the universal primers spanning the V4 region (3NDf and 1132rmod [12, 21, 34]; 566-F/915-R [37]; Ek-NS573/Ek-NSR951 [58]) and the V7–V8 regions (F-1183/R-1631 [58]) of the SSU gene have been used to amplify the SSU DNAs from nematode DNAs for Illumina MiSeq-assisted amplicon sequencing (Fig 1B). The universal SSU primer sets of Ek-NS573/Ek-NSR951 and F-1183/R-1631 that have been used by Müller et al. [58] are almost identical to the primer sets for regions U1 and U2, respectively. Müller et al. also detected a small proportion (4.8%) of nematode-derived OTUs in total eukaryotic OTUs derived from the Atlantic Forest soils as we observed in this study. On the other hand, a few taxonomic studies focusing on soil nematodes by amplicon sequencing with whole soil DNAs have been performed. For instance, Batista et al. investigated the global distributions of soil invertebrates, including nematodes, by the MiSeq-assisted sequencing of short amplicons spanning the V9 region with the universal primers (Euk1391f/EukBr) [36] (Fig 1B). Sapkota and Nicolaisen developed nematode-specific primers (NemF/18Sr2b) by modifying the published primer set NF1/18Sr2b [60] and clarified the taxonomic profiles of nematodes through high-throughput amplicon sequencing using soil DNAs purified from 22 agricultural soils via the Roche 454 platform [35] (Fig 1B). Recently, Sikder et al. have analyzed nematode taxa in the soils isolated from maize-roots by Illumina MiSeq-assisted amplicon sequencing using soil DNAs and the same primers used by Sapkota and Nicolaisen [38]. Thus, nematode-specific primers and soil DNAs seem useful for the convenient analyses of soil nematode communities without the laborious and bias-prone process of nematode isolation, if the specific and unbiased quantitative amplifications of nematode species are ensured. However, loss of taxonomic information of other eukaryotes in the soils is a fundamental issue of the nematode-focused method. By contrast, the amplicon sequencing using the universal primers and soil DNAs can identify major nematode-derived SVs as well as other eukaryote-derived SVs, providing taxonomic information covering the entire eukaryotes in parallel. However, the high depth of sequence reads may be required for this type of analysis to classify nematodes with high taxonomic resolution. In addition, some nematode species may be lost in the analyses using soil DNAs because most of the SVs with a feeding type of animal parasite were undetected in the soil DNA among the regions (S1–S6 Figs). In addition, although soil DNAs for amplifications were prepared from <0.25 g soils in prior studies using commercially available soil DNA purification kits [35, 37, 38], whether or not these amounts of soils contain sufficient numbers of soil animals, such as nematodes or earthworm, has yet to be determined.

### Nematode community structures in the copse and the cultivated agricultural field

As described in the former section, comparable results were obtained from the sequence analyses of the amplicons from the copse-derived soil DNA using the universal primers for region U2 and using the nematode-specific primers for region 4 that is suitable for the taxonomic profiling of nematodes [29, 30]. Thus, amplicon sequencing with the universal primers and soil DNA was conducted to analyze the taxonomy of the nematode community in the sweet potato-cultivated agricultural field. Four soil samples from different depths and distances from the plants were isolated at two sites of the field. The plants showed dominant and poor growth at sites 1 and 2, respectively (Fig 2B and 2C). The fractions of the nematode-derived reads were <10% of the total eukaryote-derived reads in the samples from both sites (Fig 7), as observed in the analyses using the copse-derived soil DNAs (U2 column in the right of Fig 3). The sequence reads from the site 1-derived samples were largely occupied by the order Triplonchida-derived reads. However, the contents of this order’s reads apparently decreased and the fractions of the Chromadorida- and Monhysterida-derived reads increased in the samples from site 2 (Fig 8A), suggesting distinct nematode communities in the two sampling sites. The distinct compositions of the nematode SV-derived reads in the two sample groups of sites 1 and 2 were also confirmed by beta diversity analysis (Fig 8B). Thus, the nematode communities living in the two sites were distinct, and close relations were not found in the other sample groups (i.e., distances from the plants and sampling depths) in this study. The taxonomic difference in the two sampling sites could be ascribed to the different local conditions of the soils, such as nutrient or fertility, at these sites.

Considering that feeding behavior and maturity index are useful for the ecological classification of nematodes [54], we further investigated the ecophysiological status of the nematodes living in the copse and field soils on the basis of the feeding habitat of the region U2-derived nematode SVs and the maturity index. In the analyses of the copse soil DNA-derived SVs, their major feeding types and orders were assigned to bacteria and plant feeders, and Triplonchida and Rhabditida (S8F Fig and Fig 5F). In a prior study using copse-derived individual nematodes, approximately 70% of total nematodes were derived from fungivores and plant feeders [29]. The Dorylaimida- and Rhabditida-derived plant feeders were the most abundant feeding types in a prior study that used copse-derived complex nematodes [30]. Abundant plant feeders were consistently found in current and prior studies using copse soils [29, 30]. However, the abundances of bacteria feeders were discrepant between the analyses using the soil and nematode DNAs. The fractions of bacteria feeder-derived reads commonly increased and decreased across the regions in the analyses using soil and nematode DNAs, respectively (S8 Fig), indicating that the relative abundances in feeding type and taxon (order) are influenced by template DNAs used as described in the former section. In the analyses using the field soils, the fractions of bacteria feeder-derived reads in the field samples were significantly high and occupied >60% of the total reads (S12 Fig). Bacteria feeders have been often observed as a major nematode group in agricultural field soils by amplicon sequencing [21, 22] and morphology-based studies [9, 15, 61, 62]. For instance, Rhabditida-derived bacteria feeders have been found as a major nematode group in house garden soils cultivating zucchini [30]. These previous findings agree with the observations from the sweet potato-cultivated field soils.

Nematodes can be classified by life-history strategy and by cp groups ranked from one to five [54]. Colonizers with low cp values have short generation times, exploit transient niches, lay eggs at high rates, and have high mobility and metabolic activity, and most bacteria feeders belong to this group. Persisters with high cp values lay fewer eggs than colonizers and are more susceptible to environmental disturbances, such as agricultural operations. Thus, the relative abundance of cp groups in the nematode population has been used to assess soil environmental conditions [23, 55, 63]. In this study, distinct nematode communities in the copse and field soils were shown by the relative abundances of reads derived from the families in each cp group. The majority of the copse soil DNA-derived reads was assigned to the families with high cp values, such as Prismatolaimidae (cp-3), Belondiridae (cp-5), and Trichodoridae (cp-4) (S9 Fig). By contrast, most of the field soil DNA-derived reads were derived from the families of low cp groups, including Cephalobidae, Monhysteridae (cp-2), Cyatholaimidae, and Prismatolaimidae (cp-3) (S14 Fig). These findings are consistent with our previous observations obtained from the study using the copse- and the cultivated house garden-derived nematodes [30], suggesting undisturbed conditions of the copse soil and unstable conditions of the field soil disturbed by the cultivation of sweet potato. Distinct nematode communities at the copse and field were also identified using the maturity indices. The maturity indices for the copse- and field-derived soil DNAs in region U2 were 3.13 and 2.51–2.96, respectively (Tables 4 and 6). These values are comparable to those obtained from the analyses using nematode DNAs derived from the copse (3.86–4.23) and house garden (1.58–2.73) soils in a prior study [30], although the data of the prior and current studies may be difficult to compare because of different template DNAs (i.e., soil DNA in this study and nematode-derived DNA in the previous study) [30]. Moreover, the maturity indices were likely influenced by the template DNAs used for amplification because the values of the index calculated using the nematode DNA-derived SVs were larger than those calculated using the soil DNA-derived SVs (Table 4).

## Conclusion

This study investigated nematode-derived SVs identified from complex nematode genomic DNAs and whole soil DNAs prepared from copse soils by using high-throughput amplicon sequencing with four nematode-specific and two universal primer sets for the 18S ribosomal RNA (SSU) gene. Although the primer sets for regions 1, 4 and U2 and regions 2 and U1 showed slightly different amplification profiles at the phylum-level, the numbers and the relative read abundances of nematode-derived SVs in order and feeding type were largely comparable among the six SSU regions (if the allele-derived SVs are removed). In addition, the compositions of nematode orders and feeding types derived from the nematode genomic and soil DNAs were distinct from each other. Although some different nematode SVs were found in each template DNA, the major nematode SVs were commonly detected in both template DNAs. Basing from these findings, we performed the taxonomic analyses of the nematode community in the agricultural field using amplicon sequencing with the tailed eukaryotic universal SSU primers (F1183-18S_V7-V8_MiseqF/R1631a-18S_V7-V8_MiseqR) for region U2 and whole soil DNAs. The resultant compositions of the nematode-derived SVs in family, order and feeding type, and the maturity indices virtually agree with previous findings on nematodes living in agricultural fields. Despite the undetectable minor nematode-derived SVs including allelic sequences, the resultant observations in this study suggest that the high-throughput sequencing of the amplicons using the universal primers for region 2 and soil DNA is a convenient tool for assessing the community structures of soil eukaryotes, including nematodes. In the future, the improved version of this method could be widely applied to assess the diversities and abundances of soil eukaryotes based on massive taxonomic data.

## Supporting information

S1 Table Information of the DRA-registered sequence data. (PDF)

S2 Table Copse-derived regional nematode SVs from region U2 shared with high sequence similarities. (PDF)

S3 Table Nematode-derived sequence variants (SVs) from region 1 and their taxa and feeding types based on the BLASTN search and the SILVA database. (PDF)

S4 Table Nematode-derived SVs from region 2 and their taxa and feeding types based on the BLASTN search and the SILVA database. (PDF)

S5 Table Nematode-derived SVs from region 3 and their taxa and feeding types based on the BLASTN search and the SILVA database. (PDF)

S6 Table Nematode-derived SVs from region 4 and their taxa and feeding types based on the BLASTN search and the SILVA database. (PDF)

S7 Table Nematode-derived SVs from region U1 and their taxa and feeding types based on the BLASTN search and the SILVA database. (PDF)

S8 Table Nematode-derived SVs from region U2 and their taxa and feeding types based on the BLASTN search and the SILVA database. (PDF)

S9 Table Numbers of regional nematode SVs by nematode family and colonizer–persister (cp) value. (PDF)

S10 Table Nematode-derived SVs from the agricultural soils (region U2) and their taxa and feeding types based on the BLASTN search and the SILVA database. (PDF)

S1 Fig Cladogram of nematode-derived SVs in region 1 with the corresponding orders, feeding types, and relative read abundances in each template DNA. A cladogram was prepared using nematode-derived SVs from region 1 and the corresponding SSU gene sequence of *H*. *crispae* as the outgroup as described in the Materials and methods section. Bootstrap numbers of over 500 per 1000 are indicated at the nodes of the cladogram. The name of the nematode-derived SV in region 1 was indicated as R1_SV_number at the branch end of the cladogram. Orders and feeding types of the SVs are indicated in the corresponding columns at the right of the cladogram by colored boxes and abbreviations, as shown in the legend boxes. Relative read abundances of nematode-derived SVs in the nematode genomic DNA (Nema) and soil DNA (Soil) from the copse soils are indicated by density boxes. A colored box for order with a slashed line (R1_SV_289) indicates probable nematode order due to cross-hits to the species in other phyla by BLASTN search (see S3 Table legend).
(TIFF)

S2 Fig Cladogram of nematode-derived SVs in region 2 with the corresponding orders, feeding types, and relative read abundances. A cladogram was prepared using nematode-derived SVs of region 2 as described in the Materials and methods section. Orders, feeding types, and relative read abundances of the nematode-derived SVs are indicated in the corresponding columns at the right of the cladogram by colored boxes, colored abbreviations, and density boxes, respectively, as detailed in the caption of S1 Fig.
(TIFF)

S3 Fig Cladogram of nematode-derived SVs in region 3 with the corresponding orders, feeding types, and relative read abundances. A cladogram was prepared using nematode-derived SVs of region 3 as described in the Materials and methods section. Orders, feeding types, and relative read abundances of the nematode-derived SVs are indicated in the corresponding columns at the right of the cladogram by colored boxes, colored abbreviations, and density boxes, respectively, as detailed in the caption of S1 Fig.
(TIFF)

S4 Fig Cladogram of nematode-derived SVs in region 4 with the corresponding orders, feeding types, and relative read abundances. A cladogram was prepared using nematode-derived SVs of region 4 as described in the Materials and methods section. Orders, feeding types, and relative read abundances of the nematode-derived SVs are indicated in the corresponding columns at the right of the cladogram by colored boxes, colored abbreviations, and density boxes, respectively, as detailed in the caption of S1 Fig.
(TIFF)

S5 Fig Cladogram of nematode-derived SVs in region U1 with the corresponding orders, feeding types, and relative read abundances. A cladogram was prepared using nematode-derived SVs of region U1 as described in the Materials and methods section. Orders, feeding types, and relative read abundances of the nematode-derived SVs are indicated in the corresponding columns at the right of the cladogram by colored boxes, colored abbreviations, and density boxes, respectively, as detailed in the caption of S1 Fig.
(TIFF)

S6 Fig Cladogram of nematode-derived SVs in region U2 with the corresponding orders, feeding types, and relative read abundances. A cladogram was prepared using nematode-derived SVs of region U2 as described in the Materials and methods section. Orders, feeding types, and relative read abundances of the nematode-derived SVs are indicated in the corresponding columns at the right of the cladogram by colored boxes, colored abbreviations, and density boxes, respectively, as detailed in the caption of S1 Fig.
(TIFF)

S7 Fig Numbers of regional nematode SVs from nematode and soil DNAs from the copse soils in each feeding type. Regional nematode SVs derived from the copse-derived nematode genomic DNA (blue bars) and soil DNA (red bars) were assigned to one of seven feeding types (bacteria feeder, fungus feeder, plant feeder, omnivore, predator, eukaryote feeder, and animal parasite) as described in the Materials and methods section. Numbers of regional nematode SVs in feeding type are shown by colored bars in regions 1 (A), 2 (B), 3 (C), 4 (D), U1 (E), and U2 (F), respectively. SVs with multiple feeding types are classified as “not assigned (NA).” Target SSU regions are indicated at the top right side of the corresponding bar charts.
(TIFF)

S8 Fig Relative abundances of sequence reads from six SSU regions in feeding type. Feeding types of the regional nematode SVs were assigned as described in the legend for S7 Fig. The percentages of the sequence reads of nematode SVs identified from the copse-derived nematode DNA (Nema) and soil DNA (Soil) in feeding type are shown by colored fractions of horizontal bars in regions 1 (A), 2 (B), 3 (C), 4 (D), U1 (E), and U2 (F), respectively. Each feeding type is indicated by color as shown at the bottom of (F). The fractions of SVs with multiple feeding types are classified as “not assigned” and indicated in light gray. Target SSU regions are indicated at the right side of the corresponding horizontal bar charts.
(TIFF)

S9 Fig Relative abundance of sequence reads of regional nematode SVs in each family and cp group. Percentages of sequence reads of regional nematode SVs identified from the copse-derived nematode genomic DNA (blue bars) and soil DNA (orange bars) are indicated in each family by histograms for regions 1–4, U1 and U2 (A–F), respectively. The cp-values, shown at the bottom of figure, indicate the nematode’s life strategy characteristics as described in the Materials and methods section. Nematode families are aligned by their cp values (1–5); undefined cp values are indicated by a hyphen (-). NA: not assigned to a single family. Target SSU regions are indicated at the top right side of the corresponding histograms.
(TIFF)

S10 Fig Numbers of nematode-derived SVs in order identified from four samples from sites 1 and 2 of the agricultural field. Nematode-derived SVs were obtained from four soil samples isolated from the surface (0–10) and deep (20–30) layers at the sampling points without (control) and with (plants) growing sweet potato at sites 1 (A) and 2 (B), respectively. The number of nematode-derived SVs in each sample is indicated by colored horizontal bars; the samples and colors are indicated in the legend box.
(TIFF)

S11 Fig Numbers of nematode-derived SVs in feeding type obtained from four samples from sites 1 and 2 of the agricultural field. Nematode-derived SVs identified from four soil samples at sites 1 (A) and 2 (B) of the agricultural field were assigned to one of six feeding types as described in the Materials and methods section. Four soil samples were isolated from the surface (0–10) and deep (20–30) layers at the sampling point with (plants) and without (control) growing sweet potato at each site, as shown by colors in the legend box at the bottom of the figure. Number of nematode-derived SVs in feeding type is shown in each sample by the corresponding color bar. The SVs with multiple feeding types are classified as “not assigned (NA)”.
(TIFF)

S12 Fig Relative abundances of sequence reads in feeding type in the agricultural field-derived eight soil samples. Feeding types of the nematode-derived SVs identified from eight soil samples were assigned as described in the legend for S11 Fig. The percentages of the sequence reads of nematode-derived SVs in feeding type are shown by colored fractions of horizontal bars in eight soil samples isolated from the surface (0–10) and deep (20–30) layers at the sampling point with (plants) and without (control) the growing sweet potato at site 1 (#1) and 2 (#2), respectively, as shown in the left of the corresponding histogram. Each feeding type is indicated by color as shown at the bottom of figure. The fractions of SVs with multiple feeding types are classified as “not assigned (NA)” and indicated in light gray.
(TIFF)

S13 Fig Numbers of nematode-derived SVs obtained from the agricultural field soils in family and cp-value. Nematode-derived SVs identified from soil DNAs derived from sites 1 (A) and 2 (B) of the agricultural field were assigned to nematode families based on the closest species identified by the BLASTN search. Nematode-derived SVs identified from four soil samples: The surface (0–10) and deep (20–30) layers at the sampling point with (plants) and without (control) the growing sweet potato, respectively, as shown by colors in the legend box. Number of nematode-derived SVs in family is shown by the corresponding color bar in each sample. Families are aligned by their cp values (1–5); undefined cp values are indicated by a hyphen (-). NA: not assigned to a single family.
(TIFF)

S14 Fig Relative read abundance of nematode-derived SVs obtained from the agricultural field soils in family and cp group. The relative abundance (%) of sequence reads of SVs obtained from four soil samples at sites 1 (A) and 2 (B) in each family are shown by colored horizontal bars. Soil samples were isolated from the surface (0–10) and deep (20–30) layers at the proximal (plants) and distal (control) to the sweet potato at each site, as shown by colors in the legend box. Families are aligned by their cp values (1–5); undefined cp values are indicated by a hyphen (-). NA: not assigned to a single family.
(TIFF)
