# Supplementary material for: Use of universal primers for the 18S ribosomal RNA gene and whole soil DNAs to reveal the taxonomic structures of soil nematodes by high-throughput amplicon sequencing
Source: PLoS One. 2021 Nov 15;16(11):e0259842. doi: 10.1371/journal.pone.0259842 (PMC8592498; doi:10.1371/journal.pone.0259842)
Supplement: S1 Table — (PDF) [file pone.0259842.s001.pdf]

S1 Table. Information of the DRA-registered sequence data.

ID	BioSample	Sample_name	Type	Template DNA	Sampling site(depth)	Year	Platform	Read_length	Target_gene	Region	Primer	Filename_R1	Filename_R2
TE17	SAMD00325389	copse_Z03	copse	nematode genomic DNA	copse (20-30cm)	2021	MiSeq	300bpx2	18S rDNA	region1	primerSSU18A-4F3_MiseqF/SSU_R22_MiseqR	TE17_R1.fastq	TE17_R2.fastq
TE18	SAMD00325389	copse_Z03	copse	nematode genomic DNA	copse (20-30cm)	2021	MiSeq	300bpx2	18S rDNA	region2	primerSSU_consF_MiseqF/SSU26Rplus4_MiseqR	TE18_R1.fastq	TE18_R2.fastq
TE19	SAMD00325389	copse_Z03	copse	nematode genomic DNA	copse (20-30cm)	2021	MiSeq	300bpx2	18S rDNA	region3	primerNem_18SR_ExtF_MiseqF/SSU_R23plus7_MiseqR	TE19_R1.fastq	TE19_R2.fastq
TE20	SAMD00325389	copse_Z03	copse	nematode genomic DNA	copse (20-30cm)	2021	MiSeq	300bpx2	18S rDNA	region4	primerNF1_MiseqF/18Sr2b_ExtR_MiseqR	TE20_R1.fastq	TE20_R2.fastq
TE21	SAMD00325389	copse_Z03	copse	nematode genomic DNA	copse (20-30cm)	2021	MiSeq	300bpx2	18S rDNA	regionU1	primerF574-18S_V4_MiseqF/R952-18S_V4_MiseqR	TE21_R1.fastq	TE21_R2.fastq
TE22	SAMD00325389	copse_Z03	copse	nematode genomic DNA	copse (20-30cm)	2021	MiSeq	300bpx2	18S rDNA	regionU2	primerF1183-18S_V7-V8_MiseqF/R1631a-18S_V7-V8_MiseqR	TE22_R1.fastq	TE22_R2.fastq
TE23	SAMD00325390	copse_Z03S	copse	soil DNA	copse (20-30cm)	2021	MiSeq	300bpx2	18S rDNA	region1	primerSSU18A-4F3_MiseqF/SSU_R22_MiseqR	TE23_R1.fastq	TE23_R2.fastq
TE24	SAMD00325390	copse_Z03S	copse	soil DNA	copse (20-30cm)	2021	MiSeq	300bpx2	18S rDNA	region2	primerSSU_consF_MiseqF/SSU26Rplus4_MiseqR	TE24_R1.fastq	TE24_R2.fastq
TE25	SAMD00325390	copse_Z03S	copse	soil DNA	copse (20-30cm)	2021	MiSeq	300bpx2	18S rDNA	region3	primerNem_18SR_ExtF_MiseqF/SSU_R23plus7_MiseqR	TE25_R1.fastq	TE25_R2.fastq
TE26	SAMD00325390	copse_Z03S	copse	soil DNA	copse (20-30cm)	2021	MiSeq	300bpx2	18S rDNA	region4	primerNF1_MiseqF/18Sr2b_ExtR_MiseqR	TE26_R1.fastq	TE26_R2.fastq
TE27	SAMD00325390	copse_Z03S	copse	soil DNA	copse (20-30cm)	2021	MiSeq	300bpx2	18S rDNA	regionU1	primerF574-18S_V4_MiseqF/R952-18S_V4_MiseqR	TE27_R1.fastq	TE27_R2.fastq
TE28	SAMD00325390	copse_Z03S	copse	soil DNA	copse (20-30cm)	2021	MiSeq	300bpx2	18S rDNA	regionU2	primerF1183-18S_V7-V8_MiseqF/R1631a-18S_V7-V8_MiseqR	TE28_R1.fastq	TE28_R2.fastq
TE29	SAMD00325391	field_H03S_1C10	field	soil DNA	field_site 1/control(0-10cm)	2021	MiSeq	300bpx2	18S rDNA	regionU2	primerF1183-18S_V7-V8_MiseqF/R1631a-18S_V7-V8_MiseqR	TE29_R1.fastq	TE29_R2.fastq
TE30	SAMD00325392	field_H03S_1C30	field	soil DNA	field_site 1/control(20-30cm)	2021	MiSeq	300bpx2	18S rDNA	regionU2	primerF1183-18S_V7-V8_MiseqF/R1631a-18S_V7-V8_MiseqR	TE30_R1.fastq	TE30_R2.fastq
TE31	SAMD00325393	field_H03S_1P10	field	soil DNA	field_site 1/plants(0-10cm)	2021	MiSeq	300bpx2	18S rDNA	regionU2	primerF1183-18S_V7-V8_MiseqF/R1631a-18S_V7-V8_MiseqR	TE31_R1.fastq	TE31_R2.fastq
TE32	SAMD00325394	field_H03S_1P30	field	soil DNA	field_site 1/plants(20-30cm)	2021	MiSeq	300bpx2	18S rDNA	regionU2	primerF1183-18S_V7-V8_MiseqF/R1631a-18S_V7-V8_MiseqR	TE32_R1.fastq	TE32_R2.fastq
TE33	SAMD00325395	field_H03S_2C10	field	soil DNA	field_site 2/control(0-10cm)	2021	MiSeq	300bpx2	18S rDNA	regionU2	primerF1183-18S_V7-V8_MiseqF/R1631a-18S_V7-V8_MiseqR	TE33_R1.fastq	TE33_R2.fastq
TE34	SAMD00325396	field_H03S_2C30	field	soil DNA	field_site 2/control(20-30cm)	2021	MiSeq	300bpx2	18S rDNA	regionU2	primerF1183-18S_V7-V8_MiseqF/R1631a-18S_V7-V8_MiseqR	TE34_R1.fastq	TE34_R2.fastq
TE35	SAMD00325397	field_H03S_2P10	field	soil DNA	field_site 2/plants(0-10cm)	2021	MiSeq	300bpx2	18S rDNA	regionU2	primerF1183-18S_V7-V8_MiseqF/R1631a-18S_V7-V8_MiseqR	TE35_R1.fastq	TE35_R2.fastq
TE36	SAMD00325398	field_H03S_2P30	field	soil DNA	field_site 2/plants(20-30cm)	2021	MiSeq	300bpx2	18S rDNA	regionU2	primerF1183-18S_V7-V8_MiseqF/R1631a-18S_V7-V8_MiseqR	TE36_R1.fastq	TE36_R2.fastq
