# Supplementary material for: Use of universal primers for the 18S ribosomal RNA gene and whole soil DNAs to reveal the taxonomic structures of soil nematodes by high-throughput amplicon sequencing
Source: PLoS One. 2021 Nov 15;16(11):e0259842. doi: 10.1371/journal.pone.0259842 (PMC8592498; doi:10.1371/journal.pone.0259842)
Supplement: S2 Table — (PDF) [file pone.0259842.s002.pdf]

S2 Table. Copse-derived regional nematode SVs from region U2 shared with high sequence similarities.

Cluster no.	Sequence length (No. of SVs)	SV name	Differing nt[a]	Nema[b]	Soil[b]	Order	Feeding type
1	413 bp (13)	U2_SV_10		X		Rhabditida	Animal parasite
		U2_SV_37	A/C	X		Rhabditida?	Animal parasite
		U2_SV_113	A/C	X		Rhabditida?	Animal parasite
		U2_SV_134	A/C	X		Rhabditida	Animal parasite
		U2_SV_157	A/C	X		Rhabditida	Animal parasite
		U2_SV_191	A/G	X		Rhabditida	Animal parasite
		U2_SV_209	A/C	X		Rhabditida	Animal parasite
		U2_SV_227	G/T	X		Rhabditida	Animal parasite
		U2_SV_276	A/T	X		Rhabditida	Animal parasite
		U2_SV_280	A/T	X		Rhabditida	Animal parasite
		U2_SV_287	A/C	X		Rhabditida	Animal parasite
		U2_SV_379	G/C	X		Rhabditida	Animal parasite
		U2_SV_420	(26 nt-addition)	X		Rhabditida	Animal parasite
2	413 bp (11)	U2_SV_4		X	X	Triplonchida	Plant feeder
		U2_SV_49	A/C	X	X	Triplonchida	Plant feeder
		U2_SV_63	A/C	X		Triplonchida	Plant feeder
		U2_SV_101	A/C	X		Triplonchida	Plant feeder
		U2_SV_114	G/T	X		Triplonchida	Plant feeder
		U2_SV_124	A/G	X		Triplonchida	Plant feeder
		U2_SV_160	G/T	X		Triplonchida	Plant feeder
		U2_SV_198	A/T	X		Triplonchida	Plant feeder
		U2_SV_214	A/C	X		Triplonchida	Plant feeder
		U2_SV_253	A/T	X		Triplonchida	Plant feeder
		U2_SV_286	T/G	X		Triplonchida	Plant feeder
3	413 bp (10)	U2_SV_1		X	X	Dorylaimida	Plant feeder
		U2_SV_38	A/C	X	X	Dorylaimida	Plant feeder
		U2_SV_72	G/T	X		Dorylaimida	Plant feeder
		U2_SV_105	A/C	X		Dorylaimida	Plant feeder
		U2_SV_133	G/T	X		Dorylaimida	Plant feeder
		U2_SV_155	A/T	X		Dorylaimida	Plant feeder
		U2_SV_189	A/C	X		Dorylaimida	Plant feeder
		U2_SV_192	C/A	X		Dorylaimida	Plant feeder
		U2_SV_233	C/A	X		Dorylaimida	Plant feeder
		U2_SV_260	T/G	X		Dorylaimida	Plant feeder
4	413 bp (7)	U2_SV_5		X	X	Triplonchida	Bacteria feeder
		U2_SV_56	A/C	X	X	Triplonchida	Bacteria feeder
		U2_SV_85	A/C	X		Triplonchida	Bacteria feeder
		U2_SV_90	A/C	X	X	Triplonchida	Bacteria feeder
		U2_SV_194	G/T	X		Triplonchida	Bacteria feeder
		U2_SV_216	A/T	X		Triplonchida	Bacteria feeder
		U2_SV_277	A/C	X		Triplonchida	Bacteria feeder
5	414 bp (6)	U2_SV_15		X	X	Triplonchida	Fungus feeder
		U2_SV_103	A/C	X		Triplonchida	Fungus feeder
		U2_SV_172	A/C	X		Triplonchida	Fungus feeder
		U2_SV_234	A/T	X		Triplonchida	Fungus feeder
		U2_SV_246	A/C	X		Triplonchida	Fungus feeder
		U2_SV_323	A/C	X		Triplonchida	Fungus feeder
6	412 bp (6)	U2_SV_9		X	X	Rhabditida	Plant feeder
		U2_SV_88	A/C	X		Rhabditida	Plant feeder
		U2_SV_96	A/C	X		Rhabditida	Plant feeder
		U2_SV_136	A/C	X		Rhabditida	Plant feeder
		U2_SV_285	G/T	X		Rhabditida	Plant feeder

		U2_SV_301	A/T	X		Rhabditida	Plant feeder
7	414 bp (4)	U2_SV_18		X	X	Mononchida	Predator
		U2_SV_110	A/C	X		Mononchida	Predator
		U2_SV_135	A/C	X		Mononchida	Predator
		U2_SV_336	A/T	X		Mononchida	Predator
8	413 bp (4)	U2_SV_24		X	X	Triplonchida	Bacteria feeder
		U2_SV_298	A/C		X	Triplonchida	Bacteria feeder
		U2_SV_332	A/C		X	Triplonchida	Bacteria feeder
		U2_SV_449	T/C, A/G	X		Triplonchida	Bacteria feeder
9	413 bp (3)	U2_SV_21		X	X	Dorylaimida?	Omnivore
		U2_SV_51	G/A, T/C	X		Dorylaimida?	Omnivore
		U2_SV_361	G/T	X		Dorylaimida	Omnivore
10	413 bp (3)	U2_SV_27		X	X	Rhabditida	Bacteria feeder
		U2_SV_182	A/C	X		Rhabditida	Bacteria feeder
		U2_SV_279	A/C	X		Rhabditida	Bacteria feeder
11	413 bp (2)	U2_SV_45		X	X	Plectida	Bacteria feeder
		U2_SV_334	A/C	X		Plectida	Bacteria feeder

Regional nematode SVs highly similar to other SVs were screened using ATGC software, as described in the Materials and methods section. The copse-derived nematode SVs containing less than two different sites were identified by comparing with the SV having the largest reads and shown along the abundance of their sequence reads in each cluster. The cluster number, nucleotide sequence length with number of SVs in the cluster in parentheses, SV name, differing nucleotides, SV contents in two template DNAs, order, and feeding type of SV are shown.

[a]Differing nucleotide sequences are indicated. U2_SV_37, U2_SV_10, and U2_SV_37 share one different site in their nucleotide sequences. SV_37 contains A, while the corresponding sites of the SV_10 sequence contain C.

[b]The presence of the corresponding nematode SVs in nematode DNA (Nema) and soil DNA (Soil) are indicated by an "X."
