# Supplementary material for: Use of universal primers for the 18S ribosomal RNA gene and whole soil DNAs to reveal the taxonomic structures of soil nematodes by high-throughput amplicon sequencing
Source: PLoS One. 2021 Nov 15;16(11):e0259842. doi: 10.1371/journal.pone.0259842 (PMC8592498; doi:10.1371/journal.pone.0259842)
Supplement: S3 Table — (PDF) [file pone.0259842.s003.pdf]

S3 Table. Nematode-derived sequence variants (SVs) from region 1 and their taxa and feeding types based on the BLASTN search and the SILVA database.

R1_SV[a]	BLASTN data[b]								Feeding type[c]	cp group[c]	SILVA taxonomic data[d]			
	Order	Family	Genus	Top hit	E-value	% identity	Total score	Accession no.			D7	D8	D9	D10
R1_SV_3	Dorylaimida	Belondiridae	Dorylaimellus	Dorylaimellus virginianus*	9e-174	97.78	621/665	AY552969	Plant feeder	5	Enoplea	Dorylaimia	Dorylaimida	NA
R1_SV_5	Triplonchida	Trichodoridae	Paratrichodorus	Paratrichodorus porosus	0	100	660/660	MG938558 etc	Plant feeder	4	Enoplea	Enoplia	Triplonchida	Paratrichodorus allius
R1_SV_6	Triplonchida	Prismatolaimidae	Prismatolaimus	Prismatolaimus cf. dolichurus JH-2004*	0	99.44	656/664	AY284727	Bacteria feeder	3	Enoplea	Enoplia	Triplonchida	Ambiguous_taxa
R1_SV_12	*Triplonchida*[e]	Diphterophoridae	Diphterophora	Diphterophora communis*, Diphterophora obesa*	3e-133	91.32	486/660	KY119490 etc	NA	(−)	Enoplea	Enoplia	Triplonchida	Diphtherophora obesus
R1_SV_19	Dorylaimida	Aporcelaimidae, Mydonomidae	Aporcelaimellus, Dorylaimoides	Aporcelaimellus sp.* Dorylaimoides micoletzkyi*	0	99.44	654/660	JN968252 etc	Omnivore /Fungus feeder	5/4	Enoplea	Dorylaimia	Dorylaimida	NA
R1_SV_21	Rhabditida	Thelastomatidae	Severianoia	Severianoia sp. 1 SVM-2019	4e-177	98.6	632/632	MN181511	Parasite	(−)	Chromadorea	NA	Rhabditida	Ambiguous_taxa
R1_SV_24	Rhabditida	Tylenchidae	Basiria	Basiria duplexa*	1e-171	97.24	614/669	KJ869382	Plant feeder	2	Chromadorea	NA	Rhabditida	NA
R1_SV_26	Mononchida	Mylonchulidae	Mylonchulus	Mylonchulus sp.	0	99.72	665/665	AB361447 etc	Predator	4	Enoplea	Dorylaimia	Mononchida	Ambiguous_taxa
R1_SV_35	Rhabditida	Cephalobidae	Acrobeloides	Acrobeloides sp.	0	100	662/662	MK636581 etc	Bacteria feeder	2	Chromadorea	NA	Rhabditida	NA
R1_SV_36	Dorylaimida	Aporcelaimidae, Qudsianematidae, Mydonomidae	Aporcelaimellus, Ecumenicus, Dorylaimoides	Aporcelaimellus sp.*, Ecumenicus monohystera*, Dorylaimoides sp.*	0	99.44	654/665	KY119879 etc	Omnivore /Omnivore /Fungus feeder	5/4/4	Enoplea	Dorylaimia	Dorylaimida	Ambiguous_taxa
R1_SV_39	Triplonchida	Prismatolaimidae	Prismatolaimus	Prismatolaimus cf. intermedius	0	99.72	658/664	KJ636367 etc	Bacteria feeder	3	Enoplea	Enoplia	Triplonchida	Phaseoleae environmental sample
R1_SV_44	Triplonchida	Prismatolaimidae	Prismatolaimus	Prismatolaimus cf. intermedius*	0	99.72	658/664	KJ636367 etc	Bacteria feeder	3	Enoplea	Enoplia	Triplonchida	Phaseoleae environmental sample
R1_SV_47	Dorylaimida	Tylencholaimidae, Belondiridae	Tylencholaimus, Dorylaimellus	Tylencholaimus mirabilis*, Dorylaimellus virginianus*	4e-167	96.67	599/604	EF207253 etc	Fungus feeder/Plant feeder	4/5	Enoplea	Dorylaimia	Dorylaimida	NA
R1_SV_49	Triplonchida	Odontolaimidae	Odontolaimus	Odontolaimus sp. OdLaSp1*	1e-146	93.69	531/654	FJ969131	Bacteria feeder	3	Enoplea	Enoplia	Triplonchida	NA
R1_SV_60	Plectida	Plectidae	Wilsonema	Wilsonema otophorum*	1e-176	98.34	630/664	AY593927	Bacteria feeder	2	Chromadorea	NA	Araeolaimida	NA
R1_SV_75	Dorylaimida	Belondiridae	Dorylaimellus	Dorylaimellus virginianus*	4e-172	97.5	616/660	AY552969	Plant feeder	5	Enoplea	Dorylaimia	Dorylaimida	Dorylaimellus virginianus
R1_SV_81	Rhabditida	Thelastomatidae	Severianoia	Severianoia sp. 1 SVM-2019	2e-175	98.32	627/627	MN181511	Parasite	(−)	Chromadorea	NA	NA	NA
R1_SV_97	Dorylaimida	Qudsianematidae, Actinolaimidae, Tylencholaimidae, Belondiridae	Ecumenicus, Paractinolaimus, Tylencholaimus, Dorylaimellus	Ecumenicus sp.*, Paractinolaimus macrolaimus*, Tylencholaimus mirabilis*, Dorylaimellus virginianus*	9e-169	96.94	604/610	MK292127 etc	Omnivore /Omnivore /Fungus feeder/Plant feeder	4/5/4/5	Enoplea	Dorylaimia	Dorylaimida	Ambiguous_taxa
R1_SV_98	Chromadorida	Cyatholaimidae	Achromadora	Achromadora ruricola*	4e-172	98.03	616/640	AY593941	Eucaryote feeder	3	Chromadorea	NA	Chromadorida	Achromadora ruricola
R1_SV_108	*Triplonchida*	Diphterophoridae	Diphterophora	Diphterophora communis*, Diphterophora obesa*	3e-163	96.36	586/660	KY119490 etc	NA	(−)	Enoplea	Enoplia	Triplonchida	NA
R1_SV_125	Dorylaimida	Mydonomidae	Dorylaimoides	Dorylaimoides limnophilus	0	99.44	654/654	AY593950 etc	Fungus feeder	4	Enoplea	Dorylaimia	Dorylaimida	NA
R1_SV_143	Triplonchida	Prismatolaimidae	Prismatolaimus	Prismatolaimus cf. dolichurus JH-2004	0	99.16	651/658	AY284727	Bacteria feeder	3	Enoplea	Enoplia	Triplonchida	Ambiguous_taxa
R1_SV_163	*Triplonchida*	Diphterophoridae	Diphterophora	Diphterophora communis*	2e-126	90.22	464/599	KY119490	NA	(−)	Enoplea	Enoplia	Triplonchida	Diphtherophora obesus
R1_SV_172	Rhabditida	Tylenchidae	Basiria	Basiria sp.*	9e-149	94.08	538/547	MK639391 etc	Plant feeder	2	Chromadorea	NA	Rhabditida	NA
R1_SV_176	Rhabditida	Tylenchidae	Boleodorus	Boleodorus volutus	2e-179	98.36	640/640	FJ969117	Plant feeder	2	Chromadorea	NA	Rhabditida	NA
R1_SV_210	Rhabditida	Tylenchidae	Basiria	Basiria sp. 1 JH-2014	1e-176	98.07	630/630	KJ869355	Plant feeder	2	Chromadorea	NA	Rhabditida	Neopsilenchus magnidens
R1_SV_220	Dorylaimida	Qudsianematidae, Nordiidae	Microdorylaimus, Longidorella	Microdorylaimus sp.*, Longidorella sp.*	3e-149	93.48	540/680	AJ966492 etc	Omnivore /Plant feeder	4/4	Enoplea	Dorylaimia	Dorylaimida	NA
R1_SV_224	Rhabditida	Cephalobidae	Acrobeloides	Acrobeloides sp.	0	100	662/662	AF430537 etc	Bacteria feeder	2	Chromadorea	NA	Rhabditida	NA
R1_SV_236	Triplonchida	Odontolaimidae	Odontolaimus	Odontolaimus sp. OdLaSp1*`	7e-145	93.31	525/649	FJ969131	Bacteria feeder	3	Enoplea	Enoplia	Triplonchida	NA
R1_SV_281	Rhabditida	Tylenchulidae	Paratylenchus	Paratylenchus lepidus	0	99.72	647/647	MK886695	Plant feeder	2	Chromadorea	NA	Rhabditida	Paratylenchus straeleni

R1_SV_289	Monhysterida?[f]	Monhysteridae	Eumonhystera	Eumonhystera filiformis*	3e-178	99.15	636/647	AY593937 etc	Bacteria feeder	2	Chromadorea	NA	Monhysterida	Paralamyctes environmental sample
R1_SV_292	Monhysterida	Monhysteridae	Eumonhystera	Eumonhystera sp.*	1e-166	97.43	597/619	KJ636251 etc	Bacteria feeder	2	Chromadorea	NA	Monhysterida	NA
R1_SV_313	Monhysterida	Monhysteridae	Eumonhystera	Eumonhystera cf. vulgaris 1 JH-2014	0	100	647/647	KJ636250	Bacteria feeder	2	Chromadorea	NA	Monhysterida	uncultured eukaryote
R1_SV_329	Dorylaimida	Tylencholaimidae	Tylencholaimus	Tylencholaimus sp. PDL-2005	1e-162	95.84	584/584	AJ966510	Fungus feeder	4	Enoplea	Dorylaimia	Dorylaimida	NA
R1_SV_340	Rhabditida	Tylenchidae	Coslenchus	Coslenchus sp.	0	100	658/658	KJ869314 etc	Plant feeder	2	Chromadorea	NA	Rhabditida	Aglenchus agricola
R1_SV_374	Triplonchida	Odontolaimidae	Odontolaimus	Odontolaimus sp. OdLaSp1*	1e-86	91.43	331/444	FJ969131	Bacteria feeder	3	Enoplea	Enoplia	Triplonchida	NA
R1_SV_384	Dorylaimida	Qudsianematidae, Dorylaimidae	Ecumenicus, Opisthodorylaimus	Ecumenicus sp. 85G11, Opisthodorylaimus sylphoides	9e-169	96.94	604/604	MK292127 etc	Omnivore /Omnivore	4/5	Enoplea	Dorylaimia	Dorylaimida	Ambiguous_taxa
R1_SV_475	Enoplida	Alaimidae	Alaimus	Alaimus sp. PDL-2005	0	100	662/662	AJ966514	Bacteria feeder	4	Enoplea	Enoplia	Enoplida	Alaimus sp. PDL-2005
R1_SV_490	Rhabditida	Tylenchidae	Filenchus	Filenchus discrepans	3e-178	99.15	636/636	KJ869311 etc	Fungus feeder	2	Chromadorea	NA	Rhabditida	Filenchus discrepans
R1_SV_598	Rhabditida	Tylenchidae	Filenchus	Filenchus longiurus	3e-89	84.93	340/340	KJ869337 etc	Fungus feeder	2	Chromadorea	NA	Rhabditida	Filenchus sp. 1 WB-2009
R1_SV_620	Triplonchida	Trichodoridae	Paratrichodorus	Paratrichodorus porosus	2e-180	99.16	643/643	MG938558 etc	Plant feeder	4	Enoplea	Enoplia	Triplonchida	Paratrichodorus allius
R1_SV_716	Rhabditida	Rhabditidae	Rhabditis, Pellioditis	Rhabditis sp., Pellioditis sp.	1e-162	100	584/584	LC275857 etc	Bacteria feeder/Bacteria feeder	1/1	Chromadorea	NA	Rhabditida	Rhabditis sp. DF5059
R1_SV_797	Rhabditida	Tylenchidae	Miculenchus	Miculenchus salvus*	2e-155	94.96	560/632	KY119705	Plant feeder	2	Chromadorea	NA	Rhabditida	NA

[a]The regional nematode SVs in region 1 were identified from two data sets (i.e., amplicons generated from the nematodes and soil DNAs from the copse).

[b]The top hit of species, genus, family, and order and e-value, percent identity, and total score (top hit score/highest score) from BLASTN search are indicated. The top hit sequences without taxonomic data, such as environmental samples, were omitted. The second-closest species to the query SV sequences are shown with asterisks to use the taxonomic assignment of the SVs.

[c]Feeding types and cp groups of the SVs were predicted based on those of the closest genus, based on the functional guildes at the Nemaplex home page (http://nemaplex.ucdavis.edu/Uppermnus/topmnu.htm). Feeding types and cp groups of the SVs with multile closest genus were separately indicated the corresponding types and values by slash. Parasist was predicted by previous publications as described Materials and methods section. (−): unknown cp group. NA: not assigned to the defined feeding type.

[d]The taxonomic ranks of D7–D10 corresponding to the SVs are indicated. NA: not assigned.

[e]*Diphterophora communis* was assigned to Triolonchida, as described by Kenmotsu et al. [29].

[f]Monhysterida?: probable Monhysterida due to cross-hits to the species in other phyla, such as Arthropoda.
