# Supplementary material for: Use of universal primers for the 18S ribosomal RNA gene and whole soil DNAs to reveal the taxonomic structures of soil nematodes by high-throughput amplicon sequencing
Source: PLoS One. 2021 Nov 15;16(11):e0259842. doi: 10.1371/journal.pone.0259842 (PMC8592498; doi:10.1371/journal.pone.0259842)
Supplement: S4 Table — (PDF) [file pone.0259842.s004.pdf]

S4 Table. Nematode-derived SVs from region 2 and their taxa and feeding types based on the BLASTN search and the SILVA database.

R2_SV	BLASTN data								Feeding type	cp group	SILVA taxonomic data			
	Order	Family	Genus	Top hit	E-value	% identity	Total score	Accession no.			D7	D8	D9	D10
R2_SV_3	Dorylaimida	Belondiridae	Dorylaimellus	Dorylaimellus virginianus*	7e-175	98.86	625/647	AY552969	Plant feeder	5	Enoplea	Dorylaimia	Dorylaimida	Tylencholaimus mirabilis
R2_SV_4	Triplonchida	Trichodoridae	Paratrichodorus	Paratrichodorus porosus	6e-180	100	641/641	MG938558 etc	Plant feeder	4	Enoplea	Enoplia	Triplonchida	Paratrichodorus porosus
R2_SV_6	Triplonchida	Prismatolaimidae	Prismatolaimus	Prismatolaimus cf. dolichurus JH-2004*	4e-177	99.71	632/636	AY284727	Bacteria feeder	3	Enoplea	Enoplia	Triplonchida	Ambiguous_taxa
R2_SV_9	Rhabditida	Tylenchidae	Basiria	Basiria sp.*	8e-169	97.99	604/643	KJ869382 etc	Plant feeder	2	Chromadorea	NA	Rhabditida	NA
R2_SV_12	Triplonchida	Diphtherophoridae	Diphtherophora	Diphtherophora obesus*	5e-121	89.91	446/641	KY119878	Fungus feeder	3	Enoplea	Enoplia	Triplonchida	NA
R2_SV_15	Rhabditida	Travassosinematidae	Travassosinema	Travassosinema sp. Ishigaki	1e-171	98.29	614/614	LC214832	Parasite	(−)	Chromadorea	NA	NA	NA
R2_SV_19	Mononchida	Mylonchulidae	Mylonchulus	Mylonchulus sp.	2e-180	100	643/643	KJ778171 etc	Predator	4	Enoplea	Dorylaimia	Mononchida	Ambiguous_taxa
R2_SV_20	Dorylaimida	Mydonomidae	Dorylaimoides	Dorylaimoides sp. WJW-2016	7e-180	99.71	641/641	KU662325	Fungus feeder	4	Enoplea	Dorylaimia	Dorylaimida	Nematoda environmental sample
R2_SV_26	Triplonchida	Trichodoridae	Paratrichodorus	Paratrichodorus porosus	3e-178	99.71	636/636	MG938558 etc	Plant feeder	4	Enoplea	Enoplia	Triplonchida	Paratrichodorus porosus
R2_SV_30	Triplonchida	Prismatolaimidae	Prismatolaimus	Prismatolaimus intermedius	6e-180	100	641/641	EU040133	Bacteria feeder	3	Enoplea	Enoplia	Triplonchida	Schizomidae environmental sample
R2_SV_34	Plectida	Plectidae	Ceratoplectus	Ceratoplectus sp.	2e-180	100	643/643	MN082268 etc	Bacteria feeder	2	Chromadorea	NA	Araeolaimida	Plectus sp.
R2_SV_35	Rhabditida	Travassosinematidae	Travassosinema	Travassosinema sp. Nago	1e-171	98.29	614/614	LC214829	Parasite	(−)	Chromadorea	NA	Rhabditida	NA
R2_SV_39	Triplonchida	Prismatolaimidae	Prismatolaimus	Prismatolaimus cf. dolichurus JH-2004*	2e-175	99.42	627/630	AY284727	Bacteria feeder	3	Enoplea	Enoplia	Triplonchida	Ambiguous_taxa
R2_SV_40	Dorylaimida	Mydonominae, Dorylaimidae	Dorylaimoides, Mesodorylaimus	Dorylaimoides sp. WJW-2016*, Mesodorylaimus cf. nigritulus AV-2005*	7e-160	96.29	575/592	KU66232 etc	Fungus feeder/Omnivore	4/4	Enoplea	Dorylaimia	Dorylaimida	NA
R2_SV_42	Triplonchida	Prismatolaimidae	Prismatolaimus	Prismatolaimus cf. intermedius	6e-180	100	641/641	KJ636367 etc	Bacteria feeder	3	Enoplea	Enoplia	Triplonchida	Schizomidae environmental sample
R2_SV_43	Rhabditida	Tylenchidae	Basiria	Basiria sp.*	4e-167	97.71	599/638	KJ869382 etc	Plant feeder	2	Chromadorea	NA	Rhabditida	NA
R2_SV_48	Triplonchida	Diphtherophoridae	Diphtherophora	Diphtherophora obesus*	4e-127	91.04	466/640	KY119878	Fungus feeder	3	Enoplea	Enoplia	Triplonchida	NA
R2_SV_53	Dorylaimida	Mydonomidae	Dorylaimoides	Dorylaimoides sp. WJW-2016*	3e-178	99.43	636/647	KU662325	Fungus feeder	4	Enoplea	Dorylaimia	Dorylaimida	Nematoda environmental sample
R2_SV_57	Dorylaimida	Mydonomidae	Dorylaimoides	Dorylaimoides sp. WJW-2016*	7e-175	98.86	625/647	KU662325	Fungus feeder	4	Enoplea	Dorylaimia	Dorylaimida	Nematoda environmental sample
R2_SV_61	Rhabditida	Tylenchidae	Boleodorus	Boleodorus thylactus	1e-162	96.86	584/584	MK639397 etc	Plant feeder	2	Chromadorea	NA	Rhabditida	Boleodorus thylactus
R2_SV_65	Rhabditida	Travassosinematidae	Travassosinema	Travassosinema sp. Ishigaki	7e-170	98	608/608	LC214832	Parasite	(−)	Chromadorea	NA	NA	NA
R2_SV_66	Dorylaimida	Belondiridae	Dorylaimellus	Dorylaimellus virginianus	3e-173	98.57	619/641	AY552969	Plant feeder	5	Enoplea	Dorylaimia	Dorylaimida	Tylencholaimus mirabilis
R2_SV_68	Triplonchida	Prismatolaimidae	Prismatolaimus	Prismatolaimus cf. dolichurus JH-2004*	5e-166	97.69	595/623	AY284727	Bacteria feeder	3	Enoplea	Enoplia	Triplonchida	Ambiguous_taxa
R2_SV_76	Rhabditida	Travassosinematidae	Travassosinema	Travassosinema sp. Ishigaki	7e-170	98	608/608	LC214832	Parasite	(−)	Chromadorea	NA	NA	NA
R2_SV_79	Rhabditida	Travassosinematidae	Travassosinema	Travassosinema sp. Ishigaki	7e-170	98	608/608	LC214832	Parasite	(−)	Chromadorea	NA	NA	NA
R2_SV_80	Rhabditida	Tylenchidae	Basiria	Basiria sp.	9e-164	97.13	588/588	KJ869382 etc	Plant feeder	2	Chromadorea	NA	Rhabditida	Basiria duplexa
R2_SV_81	Dorylaimida	Mydonomidae, Dorylaimidae	Dorylaimoides, Mesodorylaimus	Dorylaimoides sp. WJW-2016*, Mesodorylaimus cf. nigritulus AV-2005*	1e-161	96.57	580/592	KU662325 etc	Fungus feeder/Omnivore	4/4	Enoplea	Dorylaimia	Dorylaimida	NA
R2_SV_82	Triplonchida	Prismatolaimidae	Prismatolaimus	Prismatolaimus cf. dolichurus JH-2004*	2e-175	99.42	627/630	AY284727	Bacteria feeder	3	Enoplea	Enoplia	Triplonchida	Ambiguous_taxa
R2_SV_92	Rhabditida	Tylenchulidae	Paratylenchus	Paratylenchus lepidus	2e-179	100	640/640	MK886695	Plant feeder	2	Chromadorea	NA	Rhabditida	Paratylenchus cf. neoamblicephalus
R2_SV_95	Rhabditida	Travassosinematidae	Travassosinema	Travassosinema sp. Nago	7e-170	98	608/608	LC214829	Parasite	(−)	Chromadorea	NA	Rhabditida	NA
R2_SV_99	Triplonchida	Diphtherophoridae	Diphtherophora	Diphtherophora obesus*	3e-119	89.63	440/636	KY119878 etc	Fungus feeder	3	Enoplea	Enoplia	Triplonchida	NA

R2_SV_101	Dorylaimida	Mydonomidae	Dorylaimoides	Dorylaimoides sp. WJW-2016	7e-180	99.71	641/641	KU662325	Fungus feeder	4	Enoplea	Dorylaimia	Dorylaimida	Nematoda environmental sample
R2_SV_104	Triplonchida	Diphtherophoridae	Diphtherophora	Diphtherophora obesus*	1e-142	93.66	518/641	AY552968	Fungus feeder	3	Enoplea	Enoplia	Triplonchida	NA
R2_SV_108	Rhabditida	Tylenchidae	Filenchus	Filenchus longiurus	1e-117	89.2	435/435	KJ869337	Fungus feeder	2	Chromadorea	NA	Rhabditida	Filenchus longiurus
R2_SV_111	Rhabditida	Tylenchidae	Basiria	Basiria sp.*	4e-167	97.71	599/638	KJ869382 etc	Plant feeder	2	Chromadorea	NA	Rhabditida	Basiria duplexa
R2_SV_113	Rhabditida	Cephalobidae	Acrobeloides, Cervidellus, Cephalobus	Acrobeloides sp., Cervidellus vexilliger, Cephalobus sp.	2e-179	100	640/640	MK636581 etc	Bacteria feeder/Bacteria feeder/Bacteria feeder	2/2/2	Chromadorea	NA	Rhabditida	Acrobeles ciliatus
R2_SV_127	Dorylaimida	Tylencholaimidae	Tylencholaimus	Tylencholaimus mirabilis*	1e-167	97.44	601/651	AY284835	Fungus feeder	4	Enoplea	Dorylaimia	Dorylaimida	NA
R2_SV_144	Triplonchida	Diphtherophoridae	Diphtherophora	Diphtherophora obesus*	5e-116	89.05	429/603	AY552968	Fungus feeder	3	Enoplea	Enoplia	Triplonchida	NA
R2_SV_148	Chromadorida	Cyatholaimidae	Achromadora	Achromadora sp. JH-2004	2e-169	97.71	606/606	AY284718	Eucaryote feeder	3	Chromadorea	NA	Chromadorida	Achromadora sp. JH-2004
R2_SV_155	Rhabditida	Travassosinematidae	Travassosinema	Travassosinema sp. Ishigaki	7e-170	98	608/608	LC214832	Parasite	(−)	Chromadorea	NA	NA	NA
R2_SV_168	Rhabditida	Tylenchidae	Filenchus	Filenchus discrepans	2e-134	92.31	490/490	KJ869311 etc	Fungus feeder	2	Chromadorea	NA	Rhabditida	Filenchus discrepans
R2_SV_178	Dorylaimida	Leptonchidae	Proleptonchus	Proleptonchus weischeri	1e-166	97.43	597/597	KJ636399	Fungus feeder	4	Enoplea	Dorylaimia	Dorylaimida	Ambiguous_taxa
R2_SV_208	Triplonchida	Prismatolaimidae	Prismatolaimus	Prismatolaimus cf. dolichurus JH-2004*	3e-148	95.07	536/540	AY284727	Bacteria feeder	3	Enoplea	Enoplia	Triplonchida	Dintheria tenuissima
R2_SV_232	Dorylaimida	Tylencholaimidae	Tylencholaimus	Tylencholaimus sp.	0	100	652/652	MG921285 etc	Fungus feeder	4	Enoplea	Dorylaimia	Dorylaimida	Tylencholaimus sp. n. WJW-2016
R2_SV_244	Rhabditida	Tylenchulidae, Criconematidae	Gracilacus, Xenocriconemella, Paratylenchus	Gracilacus sp., Xenocriconemella macrodora, Paratylenchus straeleni	2e-85	85.19	327/327	MF095023 etc	Plant feeder/Plant feeder/Plant feeder	2/3/2	Chromadorea	NA	Rhabditida	NA
R2_SV_263	Rhabditida	Rhabditidae	Rhabditis	Rhabditis sp.	6e-180	100	641/641	MH608268 etc	Bacteria feeder	1	Chromadorea	NA	Rhabditida	Rhabditis sp. DF5059
R2_SV_267	Desmodorida, Chromadorida	Microlaimidae, Cyatholaimidae	Prodesmodora, Achromadora	Prodesmodora circulata*, Achromadora sp. JH-2004*	7e-160	96.29	575/592	AY284721 etc	Bacteria feeder/Eucaryote feeder	3/3	Chromadorea	NA	Chromadorida	Achromadora sp. JH-2004
R2_SV_274	Plectida	Plectidae	Ceratoplectus	Ceratoplectus sp.*	4e-177	99.43	632/643	MN082268 etc	Bacteria feeder	3	Chromadorea	NA	Araeolaimida	Plectus sp.
R2_SV_296	Monhysterida	Monhysteridae	Eumonhystera	Eumonhystera filiformis	0	100	645/645	AY593937	Bacteria feeder	3	Chromadorea	NA	Monhysterida	Eumonhystera filiformis
R2_SV_365	Rhabditida	Tylenchidae	Filenchus	Filenchus longiurus	2e-134	92.02	490/490	KJ869337	Fungus feeder	2	Chromadorea	NA	Rhabditida	NA
R2_SV_371	Rhabditida	Tylenchidae	Miculenchus	Miculenchus salvus*	1e-111	88.54	414/479	KY119705	Plant feeder	2	Chromadorea	NA	Rhabditida	NA
R2_SV_391	Rhabditida	Tylenchidae	Boleodorus	Boleodorus thylactus*	1e-171	98.29	614/649	MK639397 etc	Plant feeder	2	Chromadorea	NA	Rhabditida	Boleodorus thylactus
R2_SV_446	Triplonchida	Diphtherophoridae	Diphtherophora	Diphtherophora obesus*	9e-129	91.19	472/556	KY119878	Fungus feeder	3	Enoplea	Enoplia	Triplonchida	NA
R2_SV_476	Rhabditida	Aphelenchoididae	Aphelenchoides	Aphelenchoides sp. RH-2018	2e-160	98.77	577/577	MF070486	Fungus feeder	2	Chromadorea	NA	Rhabditida	metagenome
R2_SV_478	Monhysterida	Monhysteridae	Eumonhystera	Eumonhystera sp.	7e-145	93.75	525/525	KJ636252 etc	Bacteria feeder	3	Chromadorea	NA	Monhysterida	Mononchus aquaticus
R2_SV_480	Rhabditida	Anguinidae	Ditylenchus	Ditylenchus brevicauda	2e-121	89.89	448/448	AY284635	Fungus feeder	2	Chromadorea	NA	Rhabditida	NA
R2_SV_491	Triplonchida	Odontolaimidae	Odontolaimus	Odontolaimus sp. OdLaSp1	3e-178	99.71	636/636	FJ969131	Bacteria feeder	3	Enoplea	Enoplia	Triplonchida	Odontolaimus sp. OdLaSp1
R2_SV_502	Enoplida	Trischistomatidae	Trischistoma	Trischistoma sp.	0	100	651/651	KR492034 etc	Predator	3	Enoplea	Enoplia	Triplonchida	NA
R2_SV_540	Triplonchida	Trichodoridae	Paratrichodorus	Paratrichodorus porosus	2e-149	100	540/540	DQ345524	Plant feeder	4	Chromadorea	NA	Rhabditida	Cactodera sp. WY-2012
R2_SV_546	Rhabditida	Tylenchidae	Miculenchus	Miculenchus salmae*	3e-88	84.53	337/560	MF599079	Plant feeder	2	Chromadorea	NA	Rhabditida	NA

Note: See notes in S3 Table.
