# Supplementary material for: Use of universal primers for the 18S ribosomal RNA gene and whole soil DNAs to reveal the taxonomic structures of soil nematodes by high-throughput amplicon sequencing
Source: PLoS One. 2021 Nov 15;16(11):e0259842. doi: 10.1371/journal.pone.0259842 (PMC8592498; doi:10.1371/journal.pone.0259842)
Supplement: S5 Table — (PDF) [file pone.0259842.s005.pdf]

S5 Table. Nematode-derived SVs from region 3 and their taxa and feeding types based on the BLASTN search and the SILVA database.

R3_SV	BLASTN data								Feeding type	cp group	SILVA taxonomic data			
	Order	Family	Genus	Top hit	E-value	% identity	Total score	Accession no.			D7	D8	D9	D10
R3_SV_1	Dorylaimida	Belondiridae	Dorylaimellus	Dorylaimellus parvulus	2e-149	100	540/540	AY911968	Plant feeder	5	Enoplea	Dorylaimia	Dorylaimida	NA
R3_SV_3	Triplonchida	Prismatolaimidae	Prismatolaimus	Prismatolaimus cf. dolichurus JH-2004	2e-149	100	540/540	AY284727	Bacteria feeder	3	Enoplea	Enoplia	Triplonchida	Ambiguous_taxa
R3_SV_4	Rhabditida	Tylenchidae	Discopersicus	Discopersicus iranicus	7e-149	100	538/538	KM502981	*Plant feeder* [a]	*2* [a]	Chromadorea	NA	Rhabditida	NA
R3_SV_5	Triplonchida	Trichodoridae	Paratrichodorus	Paratrichodorus porosus	3e-148	100	536/536	DQ345524	Plant feeder	4	Enoplea	Enoplia	Triplonchida	Paratrichodorus porosus
R3_SV_7	Triplonchida	Prismatolaimidae	Prismatolaimus	Prismatolaimus sp.	2e-149	100	540/540	LC186851 etc	Bacteria feeder	3	Enoplea	Enoplia	Triplonchida	Schizomidae environmental sample
R3_SV_8	Rhabditida	Thelastomatidae	Cephalobellus, Thelastoma, Travassosinema	Cephalobellus brevicaudatus, Thelastoma sp., Travassosinema sp.	7e-149	100	538/538	MF668724 etc	Parasite	(−)	Chromadorea	NA	Rhabditida	NA
R3_SV_10	Dorylaimida	Qudsianematidae, Dorylaimidae, Aporcelaimidae	Ecumenicus, Mesodorylaimus, Aporcelaimellus	Ecumenicus sp., Mesodorylaimus sp., Aporcelaimellus sp.	2e-149	100	540/540	MK292127 etc	Omnivore/Omnivore/ Omnivore	4/4/5	Enoplea	Dorylaimia	Dorylaimida	Ambiguous_taxa
R3_SV_12	Mononchida	Mylonchulidae	Mylonchulus	Mylonchulus sp.	2e-149	100	540/540	AB361447 etc	Predator	4	Enoplea	Dorylaimia	Mononchida	NA
R3_SV_15	Rhabditida	Cephalobidae	Acrobeloides, Cephalobus, Pseudacrobeles, Eucephalobus, Chiloplacus	Acrobeloides sp., Cephalobus sp., Pseudacrobeles sp., Eucephalobus sp., Chiloplacus sp.	7e-149	100	538/538	MK636581etc	Bacteria feeder/Bacteria feeder/Bacteria feeder/Bacteria feeder/Bacteria feeder	2/2/2/2/2	Chromadorea	NA	Rhabditida	NA
R3_SV_17	Triplonchida	Trichodoridae	Paratrichodorus	Paratrichodorus allius*	2e-119	95.02	440/532	MG938581 etc	Plant feeder	4	Enoplea	Enoplia	Triplonchida	NA
R3_SV_21	Triplonchida	Odontolaimidae	Odontolaimus	Odontolaimus sp. OdLaSp1*	2e-125	95.49	460/532	FJ969131	Bacteria feeder	3	Enoplea	Enoplia	Triplonchida	NA
R3_SV_25	Dorylaimida	Belondiridae	Dorylaimellus	Dorylaimellus parvulus	9e-148	99.66	534/534	AY911968	Plant feeder	5	Enoplea	Dorylaimia	Dorylaimida	NA
R3_SV_26	Plectida	Plectidae	Plectus, Ceratoplectus, Tylocephalus	Plectus sp., Ceratoplectus sp., Tylocephalus sp.	7e-149	100	538/538	LC382088 etc	Bacteria feeder/Bacteria feeder/NA [b]	2/2/(−)	Chromadorea	NA	Araeolaimida	Ambiguous_taxa
R3_SV_28	Chromadorida	Cyatholaimidae	Achromadora	Achromadora ruricola	7e-149	100	538/538	AY593941	Eucaryote feeder	3	Chromadorea	NA	Chromadorida	NA
R3_SV_32	Dorylaimida	Longidoridae, Dorylaimidae	Longidorus, Paralongidorus, Calcaridorylaimus, Mesodorylaimus, Laimydorus	Longidorus sp., Paralongidorus sp., Calcaridorylaimus sp., Mesodorylaimus sp., Laimydorus sp.	2e-149	100	540/540	MN129758 etc	Plant feeder/Plant feeder/Omnivore/Om nivore/Omnivore	5/5/4/4/4	Enoplea	Dorylaimia	Dorylaimida	Ambiguous_taxa
R3_SV_35	Rhabditida	Tylenchulidae	Paratylenchus	Paratylenchus lepidus	7e-149	100	538/538	MK886695	Plant feeder	2	Chromadorea	NA	Rhabditida	Hemicycliophora conida
R3_SV_36	Rhabditida	Tylenchidae	Filenchus	Filenchus cf. helenae TSH-2005	1e-81	86.51	315/315	AY912032	Fungus feeder	2	Chromadorea	NA	Rhabditida	NA
R3_SV_45	Rhabditida	Tylenchidae, Merliniidae, Telotylenchidae	Atetylenchus, Geocenamus, Psilenchus, Aglenchus, Trophurus	Atetylenchus sp., Geocenamus chengi, Psilenchus hilarulus, Aglenchus geraerti, Trophurus wuhuensis	2e-145	99.31	527/527	MN807627 etc	Plant feeder/Plant feeder/Plant feeder/Plant feeder/Plant feeder	2/3/2/2/3	Chromadorea	NA	Rhabditida	NA
R3_SV_48	Rhabditida	Tylenchidae	Basiria	Basiria cf. similis TSH-2005	2e-149	100	540/540	AY911922	Plant feeder	2	Chromadorea	NA	Rhabditida	NA

R3_SV_58	Dorylaimida	Aporcelaimidae, Tylencholaimidae, Longidoridae, Qudsianematidae, Leptonchidae	Aporcella, Tylencholaimus, Xiphidorus, Lordellonema, Akrotonus, Funaria	Aporcella vitrinus, Tylencholaimus cf. teres, Xiphidorus sp., Lordellonema cf. parvum, Akrotonus vigor, Funaria cacti	9e-148	99.66	534/534	MG921235 etc	Omnivore/Fungus feeder/Plant feeder/Omnivore/NA /Fungus feeder	5/4/5/4/(−)/4	Enoplea	Dorylaimia	Dorylaimida	NA
R3_SV_63	Monhysterida	Monhysteridae	Eumonhystera	Eumonhystera cf. hungarica 1 JH-2014	9e-143	98.96	518/518	KJ636237	Bacteria feeder	3	Chromadorea	NA	Monhysterida	NA
R3_SV_67	Rhabditida	Tylenchidae	Filenchus	Filenchus cf. helenae TSH-2005	7e-149	100	538/538	AY912032	Fungus feeder	2	Chromadorea	NA	Rhabditida	Filenchus discrepans
R3_SV_68	Triplonchida	Diphtherophoridae	Diphtherophora	Diphtherophora sp. 803S-002*	2e-130	97.49	477/532	EU880005	Fungus feeder	3	Enoplea	Enoplia	Triplonchida	Nematoda environmental sample
R3_SV_70	Rhabditida	Tylenchidae	Atetylenchus, Coslenchus, Psilenchus	Atetylenchus sp., Coslenchus sp., Psilenchus sp.	7e-149	100	538/538	MN807629 etc	Plant feeder/Plant feeder/Plant feeder/	2/2/2	Chromadorea	NA	Rhabditida	NA
R3_SV_76	Monhysterida?	Monhysteridae	Eumonhystera	Eumonhystera filiformis*	2e-145	99.31	527/538	AY593937 etc	Bacteria feeder	3	Chromadorea	NA	Monhysterida	NA
R3_SV_80	Enoplida	Alaimidae	Alaimus	Alaimus sp. PDL-2005	1e-141	98.29	514/514	AJ966514	Bacteria feeder	4	Enoplea	Enoplia	Enoplida	Alaimus sp. PDL-2005
R3_SV_85	Rhabditida	Rhabditidae	Rhabditis	Rhabditis sp.	3e-148	100	536/536	HQ13050 etc	Bacteria feeder	1	Chromadorea	NA	Rhabditida	Rhabditis sp. DF5059
R3_SV_90	Monhysterida	Monhysteridae	Eumonhystera	Eumonhystera cf. hungarica 1 JH-2014*	2e-139	98.27	507/512	KJ636237	Bacteria feeder	3	Chromadorea	NA	Monhysterida	Mononchus aquaticus
R3_SV_94	Rhabditida	Thelastomatidae	Cephalobellus, Thelastoma, Travassosinema	Cephalobellus brevicaudatus, Thelastoma sp., Travassosinema sp.	3e-147	99.66	532/532	MF668724 etc	Parasite	(−)	Chromadorea	NA	Rhabditida	NA
R3_SV_96	Rhabditida	Cephalobidae	Cephalobus	Cephalobus cubaensis*	3e-147	99.66	532/538	AF202161	Bacteria feeder	2	Chromadorea	NA	Rhabditida	Cephalobus cubaensis
R3_SV_103	Monhysterida	Monhysteridae	Eumonhystera	Eumonhystera cf. hungarica 1 JH-2014	4e-141	98.62	512/512	KJ636237	Bacteria feeder	3	Chromadorea	NA	Monhysterida	Mononchus aquaticus
R3_SV_108	Chromadorida	Cyatholaimidae	Achromadora	Achromadora cf terricola, Achromadora sp.	7e-149	100	538/538	AY593940 etc	Eukaryote feeder	3	Chromadorea	NA	Chromadorida	NA
R3_SV_109	Dorylaimida	Qudsianematidae	Chrysonema	Chrysonema attenuatum	4e-146	99.32	529/529	EF207245 etc	Omnivore	4	Enoplea	Dorylaimia	Dorylaimida	Ambiguous_taxa
R3_SV_111	Monhysterida?	Monhysteridae	Eumonhystera	Eumonhystera filiformis*	7e-144	98.47	521/538	AY593937etc	Bacteria feeder	3	Chromadorea	NA	Monhysterida	metagenome
R3_SV_114	Dorylaimida	Tylencholaimellidae, Aporcelaimidae, Mydonomidae	Tylencholaimellus, Paraxonchium, Dorylaimoides, Doryllium	Tylencholaimellus sp., Paraxonchium magnidens, Dorylaimoides cf. elegans, Doryllium sp.	2e-149	100	540/540	KP835681etc	Fungus feeder/Omnivore/Fungus feeder/Fungus feeder	4/5/4/4	Enoplea	Dorylaimia	Dorylaimida	Ambiguous_taxa
R3_SV_123	Monhysterida	Monhysteridae	Eumonhystera	Eumonhystera cf. hungarica 1 JH-2014*	4e-146	99.65	529/534	KJ636237	Bacteria feeder	3	Chromadorea	NA	Monhysterida	Mononchus aquaticus
R3_SV_126	Chromadorida	Cyatholaimidae	Achromadora	Achromadora sp.	2e-145	99.31	527/527	AY593941etc	Eucaryote feeder	3	Chromadorea	NA	Chromadorida	NA
R3_SV_134	Triplonchida	Prismatolaimidae	Prismatolaimus	Prismatolaimus sp.	4e-146	99.32	529/529	LC186851etc	Bacteria feeder	3	Enoplea	Enoplia	Triplonchida	Schizomidae environmental sample
R3_SV_148	Dorylaimida	Tylencholaimidae	Tylencholaimus	Tylencholaimus proximus	2e-149	100	540/540	AY146540	Fungus feeder	4	Enoplea	Dorylaimia	Dorylaimida	NA
R3_SV_150	Rhabditida	Tylenchidae	Filenchus, Basiria	Filenchus sp., Basiria clavicaudata	3e-147	99.66	532/532	MH844388 etc	Fungus feeder/Plant feeder	2/2	Chromadorea	NA	Rhabditida	Filenchus discrepans
R3_SV_164	Dorylaimida	Nygolaimidae	Clavicaudoides, Aquatides	Clavicaudoides sp., Aquatides christei	2e-149	100	540/540	AY552967 etc	Predator/Predator	5/5	Enoplea	Dorylaimia	Dorylaimida	Nygolaimus cf. brachyuris JH-2004
R3_SV_165	Rhabditida	Thelastomatidae	Cephalobellus, Thelastoma, Travassosinema	Cephalobellus brevicaudatus, Thelastoma sp., Travassosinema sp.	2e-145	99.31	527/527	MF668724 etc	Parasite	(−)	Chromadorea	NA	Rhabditida	NA
R3_SV_173	Rhabditida	Tylenchidae	Thada	Thada sp.*	2e-145	99.31	527/531	MN557353	Plant feeder	2	Chromadorea	NA	Rhabditida	NA

R3_SV_191	Rhabditida	Tylenchidae, Anguinidae	Discotylenchus, Filenchus, Ditylenchus	Discotylenchus sp., Filenchus misellus, Ditylenchus brevicauda	6e-150	100	542/542	MK301105 etc	Plant feeder/Fungus feeder/Fungus feeder	2/2/2	Chromadorea	NA	Rhabditida	NA
R3_SV_205	Chromadorida	Cyatholaimidae	Achromadora	Achromadora sp.	6e-125	95.19	459/459	AY593941etc	Eucaryote feeder	3	Chromadorea	NA	Chromadorida	NA
R3_SV_214	Rhabditida	Tylenchidae	Filenchus	Filenchus cf. helenae TSH-2005	6e-80	86.16	309/309	AY912032	Fungus feeder	2	Chromadorea	NA	NA	NA
R3_SV_234	Dorylaimida	Longidoridae, Dorylaimidae	Longidorus, Paralongidorus, Calcaridorylaimus, Mesodorylaimus, Laimydorus	Longidorus sp., Paralongidorus sp., Calcaridorylaimus sp., Mesodorylaimus sp., Laimydorus sp.	7e-139	97.95	505/505	MN129758 etc	Plant feeder/Plant feeder/Omnivore/Omnivore/Omnivore	5/5/4/4/4	Enoplea	Dorylaimia	Dorylaimida	NA
R3_SV_252	Rhabditida	Aphelenchoididae	Aphelenchoides	Aphelenchoides sp.*	7e-144	98.97	521/523	KY769062 etc	Fungus feeder	2	Chromadorea	NA	Rhabditida	Aphelenchoides bicaudatus
R3_SV_260	Rhabditida	Cephalobidae	Acrobeloides, Cephalobus, Pseudacrobeles, Eucephalobus, Chiloplacus	Acrobeloides sp., Cephalobus sp., Pseudacrobeles sp., Eucephalobus sp., Chiloplacus sp.	1e-116	94.6	413/431	MK636581etc	Bacteria feeder/Bacteria feeder/Bacteria feeder/Bacteria feeder/Bacteria feeder	2/2/2/2/2	Chromadorea	NA	Rhabditida	NA
R3_SV_272	Rhabditida	Tylenchidae	Discotylenchus, Filenchus, Ditylenchus	Discotylenchus sp., Filenchus misellus, Ditylenchus brevicauda	4e-146	99.32	529/529	MK301105 etc	Plant feeder/Fungus feeder/Fungus feeder	2/2/2	Chromadorea	NA	Rhabditida	NA

Note: See notes in S3 Table.

[a]The feeding type and cp value of the SV in italic were predicted by the closest family based on the functional guildes at the Nemaplex home page (http://nemaplex.ucdavis.edu/Uppermnus/topmnu.htm).
