# Supplementary material for: Use of universal primers for the 18S ribosomal RNA gene and whole soil DNAs to reveal the taxonomic structures of soil nematodes by high-throughput amplicon sequencing
Source: PLoS One. 2021 Nov 15;16(11):e0259842. doi: 10.1371/journal.pone.0259842 (PMC8592498; doi:10.1371/journal.pone.0259842)
Supplement: S6 Table — (PDF) [file pone.0259842.s006.pdf]

S6 Table. Nematode-derived SVs from region 4 and their taxa and feeding types based on the BLASTN search and the SILVA database.

R4_SV	BLASTN data								Feeding type	cp group	SILVA taxonomic data			
	Order	Family	Genus	Top hit	E-value	% identity	Total score	Accession no.			D7	D8	D9	D10
R4_SV_1	Dorylaimida	Belondiridae	Dorylaimellus	Dorylaimellus parvulus	2e-165	100	593/593	AY911968	Plant feeder	5	Enoplea	Dorylaimia	Dorylaimida	Proleptonchus weischeri
R4_SV_4	Triplonchida	Prismatolaimidae	Prismatolaimus	Prismatolaimus sp.	2e-165	100	593/593	LC186686 etc	Bacteria feeder	3	Enoplea	Enoplia	Triplonchida	Ambiguous_taxa
R4_SV_7	Triplonchida	Trichodoridae	Paratrichodorus	Paratrichodorus porosus	2e-165	100	593/593	MG938571 etc	Plant feeder	4	Enoplea	Enoplia	Triplonchida	Paratrichodorus porosus
R4_SV_12	Rhabditida	Cosmocercidae, Thelastomatidae	Cosmocerca, Cephalobellus	Cosmocerca simile*, Cephalobellus brevicaudatus*	1e-161	99.38	580/592	MN839758 etc	Parasite	(−)	Chromadorea	NA	Rhabditida	Ambiguous_taxa
R4_SV_13	Rhabditida	Tylenchidae	Discopersicus, Basiria	Discopersicus iranicus, Basiria duplexa	6e-165	100	592/592	KM502981 etc	NA/Plant feeder	(−)/2	Chromadorea	NA	Rhabditida	Basiria duplexa
R4_SV_19	Triplonchida	Diphtherophoridae	Diphtherophora	Diphtherophora sp. Shahrood*	5e-121	92.38	446/595	KY115102	Fungus feeder	3	NA	NA	NA	NA
R4_SV_21	Dorylaimida	Mydonomidae, Dorylaimidae	Dorylaimoides, Dorylaimus, Thornenema	Dorylaimoides sp., Dorylaimus stagnalis, Thornenema cf. laevicapitatum	8e-164	99.69	588/588	KU662325 etc	Fungus feeder/Omnivore/ Omnivore	4/4/5	Enoplea	Dorylaimia	Dorylaimida	Ambiguous_taxa
R4_SV_22	Mononchida	Mylonchulidae	Mylonchulus	Mylonchulus sigmaturus	5e-166	100	595/595	AB361447 etc	Predator	4	Enoplea	Dorylaimia	Mononchida	Mylonchulus sp. JH-2004
R4_SV_29	Triplonchida	Prismatolaimidae	Prismatolaimus	Prismatolaimus sp.	2e-165	100	593/593	LC186851 etc	Bacteria feeder	3	Enoplea	Enoplia	Triplonchida	Ambiguous_taxa
R4_SV_32	Dorylaimida	Leptonchidae	Leptonchus	Leptonchus microdens	5e-146	96.28	529/529	AY146539	Fungus feeder	4	Enoplea	Dorylaimia	Dorylaimida	NA
R4_SV_41	Rhabditida	Cephalobidae	Acrobeloides, Cephalobus	Acrobeloides sp., Cephalobus sp.	2e-165	100	593/593	MK636581 etc	Bacteria feeder/Bacteria feeder	2/2	Chromadorea	NA	Rhabditida	NA
R4_SV_46	Rhabditida	Cosmocercidae, Thelastomatidae	Cosmocerca, Cephalobellus	Cosmocerca simile*, Cephalobellus brevicaudatus*	6e-160	99.06	575/586	MN839758 etc	Parasite	(−)	Chromadorea	NA	Rhabditida	Ambiguous_taxa
R4_SV_50	Rhabditida	Tylenchidae	Discopersicus, Basiria	Discopersicus iranicus, Basiria duplexa	3e-163	99.69	586/586	KM502981 etc	NA/Plant feeder	(−)/2	Chromadorea	NA	Rhabditida	Basiria duplexa
R4_SV_51	Dorylaimida	Mydonomidae, Dorylaimidae	Dorylaimoides, Dorylaimus, Thornenema	Dorylaimoides sp.*, Dorylaimus stagnalis*, Thornenema cf. laevicapitatum*	8e-164	99.69	588/593	KU662325 etc	Fungus feeder/Omnivore/ Omnivore	4/4/5	Enoplea	Dorylaimia	Dorylaimida	NA
R4_SV_53	Plectida, (Rhabditida)[a]	Plectidae, (Cephalobidae)[a]	Plectus, Ceratoplectus, Wilsonema, (Acrobeloides)[a]	Plectus sp., Ceratoplectus cf. armatus, Wilsonema sp., (Acrobeloides buetschlii)[a]	2e-165	100	593/593	LC186814 etc	Bacteria feeder/Bacteria feeder/Bacteria feeder	2/2/2	Chromadorea	NA	Araeolaimida	Acrobeloides buetschlii
R4_SV_55	Triplonchida	Diphtherophoridae	Diphtherophora	Diphtherophora sp.*	2e-134	94.1	490/595	KY115102 etc	Fungus feeder	3	Enoplea	Enoplia	Triplonchida	Odontolaimus sp. OdLaSp1
R4_SV_56	Dorylaimida	Mydonomidae, Dorylaimidae	Dorylaimoides, Dorylaimus, Thornenema	Dorylaimoides sp.*, Dorylaimus stagnalis*, Thornenema cf. laevicapitatum*	4e-162	99.38	582/593	KU662325 etc	Fungus feeder/Omnivore/ Omnivore	4/4/5	Enoplea	Dorylaimia	Dorylaimida	NA
R4_SV_83	Rhabditida	Tylenchidae	Boleodorus	Boleodorus cf. thylactus TSH-2005	4e-157	97.31	566/566	AY911926	Plant feeder	2	Chromadorea	NA	Rhabditida	Boleodorus thylactus
R4_SV_91	Dorylaimida	Mydonomidae	Dorylaimoides	Dorylaimoides sp.*	8e-159	98.75	571/577	KU662325 etc	Fungus feeder	4	Enoplea	Dorylaimia	Dorylaimida	NA
R4_SV_98	Triplonchida, (Dorylaimida)	Diphtherophoridae, (Diphterophoridae)	Diphtherophora, (Diphterophora)	Diphtherophora sp.*, (Diphterophora obesa*)	4e-142	95.65	516/593	AY552968 etc	Fungus feeder	3	Enoplea	Enoplia	Triplonchida	NA
R4_SV_99	Rhabditida	Tylenchidae	Malenchus	Malenchus sp.	2e-104	88.75	390/390	LC186800 etc	Plant feeder	2	Chromadorea	NA	Rhabditida	NA
R4_SV_100	Rhabditida	Tylenchulidae	Paratylenchus	Paratylenchus lepidus	6e-165	100	592/592	MK886695	Plant feeder	2	Chromadorea	NA	Rhabditida	NA
R4_SV_103	Rhabditida	Tylenchidae	Basiria	Basiria cf. obliquua TSH-2005	6e-155	98.13	558/558	AY911919	Plant feeder	2	Chromadorea	NA	Rhabditida	Neopsilenchus magnidens
R4_SV_107	Chromadorida	Cyatholaimidae	Achromadora	Achromadora sp. JH-2004	5e-166	100	595/595	AY284717	Eucaryote feeder	3	Chromadorea	NA	Chromadorida	Achromadora sp. JH-2004
R4_SV_111	Triplonchida	Trichodoridae	Paratrichodorus	Paratrichodorus porosus	8e-164	99.64	588/588	MG938571 etc	Plant feeder	4	Enoplea	Enoplia	Triplonchida	Paratrichodorus porosus

R4_SV_115	Rhabditida	Cephalobidae	Acrobeloides, Cephalobus	Acrobeloides sp., Cephalobus sp.	8e-164	99.69	588/588	MK636581 etc	Bacteria feeder/Bacteria feeder	2/2	Chromadorea	NA	Rhabditida	NA
R4_SV_135	Rhabditida	Tylenchidae	Coslenchus	Coslenchus sp.	6e-165	100	592/592	MN542199 etc	Plant feeder	2	Chromadorea	NA	Rhabditida	NA
R4_SV_158	Triplonchida	Diphtherophoridae	Diphtherophora	Diphtherophora sp. Shahrood*	1e-127	92.9	468/490	KY115102	Fungus feeder	3	Enoplea	Enoplia	Triplonchida	NA
R4_SV_175	Rhabditida	Cosmocercidae, Thelastomatidae	Cosmocerca, Cephalobellus	Cosmocerca simile*, Cephalobellus brevicaudatus*	6e-160	99.06	575/586	MN839758 etc	Parasite	(−)	Chromadorea	NA	Rhabditida	Ambiguous_taxa
R4_SV_216	Triplonchida	Diphtherophoridae	Diphtherophora	Diphtherophora sp. Shahrood*	2e-119	92.06	440/590	KY115102	Fungus feeder	3	NA	NA	NA	NA
R4_SV_230	Rhabditida	Cephalobidae	Eucephalobus, Cephalobus	Eucephalobus sp., Cephalobus sp.	2e-165	100	593/593	AY912001 etc	Bacteria feeder/Bacteria feeder	2/2	Chromadorea	NA	Rhabditida	NA
R4_SV_254	Triplonchida	Prismatolaimidae	Prismatolaimus	Prismatolaimus sp.	2e-165	100	593/593	LC186858 etc	Bacteria feeder	3	Enoplea	Enoplia	Triplonchida	Ambiguous_taxa
R4_SV_277	Rhabditida	Tylenchidae	Filenchus	Filenchus sp.	6e-165	99.69	592/592	KJ869311 etc	Fungus feeder	2	Chromadorea	NA	Rhabditida	Filenchus discrepans
R4_SV_298	Monhysterida	Monhysteridae	Eumonhystera	Eumonhystera sp.*	3e-158	98.45	569/584	KJ636251 etc	Bacteria feeder	3	Chromadorea	NA	Monhysterida	NA
R4_SV_302	Dorylaimida	Tylencholaimidae	Tylencholaimus	Tylencholaimus sp.*	3e-158	98.45	569/597	LC186596 etc	Fungus feeder	4	Enoplea	Dorylaimia	Dorylaimida	NA
R4_SV_308	Rhabditida	Rhabditidae	Rhabditis	Rhabditis sp.	6e-165	100	592/592	HQ130504 etc	Bacteria feeder	1	Chromadorea	NA	Rhabditida	Rhabditis sp. DF5059
R4_SV_315	Dorylaimida	Belondiridae	Dorylaimellus	Dorylaimellus parvulus	6e-150	100	542/542	AY911968	Plant feeder	5	Chromadorea	NA	Rhabditida	Globodera rostochiensis
R4_SV_369	Rhabditida	Aphelenchoididae	Aphelenchoides	Aphelenchoides sp.*	8e-139	95.28	505/579	KY769062	Fungus feeder	2	Chromadorea	NA	Rhabditida	metagenome
R4_SV_405	Dorylaimida	Tylencholaimidae	Tylencholaimus	Tylencholaimus sp.	5e-166	100	595/595	MG921285 etc	Fungus feeder	4	Enoplea	Dorylaimia	Dorylaimida	Tylencholaimus sp. n. WJW-2016
R4_SV_410	Monhysterida?	Monhysteridae	Eumonhystera	Eumonhystera filiformis*	8e-154	97.83	555/566	AY593937 etc	Bacteria feeder	3	Chromadorea	NA	Monhysterida	NA
R4_SV_415	Dorylaimida	Aporcelaimidae	Aporcella	Aporcella vitrinus*	4e-147	96.57	532/538	MG921235 etc	Omnivore	5	Enoplea	Dorylaimia	Dorylaimida	NA
R4_SV_438	Dorylaimida	Belondiridae	Axonchoides, Oxydirus	Axonchoides smokyensis*, Oxydirus nethus*	1e-146	96.57	531/564	JX885740 etc	Plant feeder/Predator	5/5	Enoplea	Dorylaimia	Dorylaimida	NA
R4_SV_443	Dorylaimida	Belondiridae	Dorylaimellus	Dorylaimellus parvulus	0	100	749/749	AY911968	Plant feeder	5	Enoplea	Dorylaimia	Dorylaimida	Proleptonchus weischeri
R4_SV_489	Triplonchida	Prismatolaimidae	Prismatolaimus	Prismatolaimus cf. dolichurus JH-2004	6e-150	100	542/542	AY284727	Bacteria feeder	3	Chromadorea	NA	Rhabditida	Globodera rostochiensis
R4_SV_497	Monhysterida?	Monhysteridae	Eumonhystera	Eumonhystera filiformis*	4e-162	99.38	577/593	AY593937 etc	Bacteria feeder	3	Chromadorea	NA	Monhysterida	Paralamyctes environmental sample
R4_SV_501	Rhabditida	Aphelenchoididae	Aphelenchoides	Aphelenchoides sp.*	6e-130	93.52	475/523	KY769066 etc	Fungus feeder	2	Chromadorea	NA	Rhabditida	NA
R4_SV_559	Triplonchida	Trichodoridae	Paratrichodorus	Paratrichodorus sp.	0	100	749/749	MG938571 etc	Plant feeder	4	Enoplea	Enoplia	Triplonchida	Paratrichodorus porosus
R4_SV_561	Triplonchida	Prismatolaimidae	Prismatolaimus	Prismatolaimus sp.	0	100	749/749	LC186686 etc	Bacteria feeder	3	Enoplea	Enoplia	Triplonchida	NA
R4_SV_566	Rhabditida	Meloidogynidae	Meloidogyne	Meloidogyne ichinohei*	1e-112	90.34	418/536	KC875385	Plant feeder	3	Chromadorea	NA	Rhabditida	NA
R4_SV_588	Monhysterida?	Monhysteridae	Eumonhystera	Eumonhystera filiformis*	2e-155	98.13	560/580	AY593937 etc	Bacteria feeder	3	Chromadorea	NA	Monhysterida	metagenome
R4_SV_593	Enoplida	Alaimidae	Alaimus	Alaimus sp.	8e-164	99.69	588/588	LC186877 etc	Bacteria feeder	4	Enoplea	Enoplia	Enoplida	Alaimus sp. PDL-2005
R4_SV_610	Rhabditida	Thelastomatidae, Travassosinematidae	Cephalobellus, Thelastoma, Travassosinema	Cephalobellus brevicaudatus, Thelastoma sp., Travassosinema sp.	2e-149	100	540/540	MF668724 etc	Parasite	(−)	Chromadorea	NA	Rhabditida	Globodera rostochiensis
R4_SV_634	Rhabditida	Cosmocercidae, Thelastomatidae	Cosmocerca, Cephalobellus	Cosmocerca simile*, Cephalobellus brevicaudatus*	0	99.5	736/747	MN839758 etc	Parasite	(−)	Chromadorea	NA	NA	NA
R4_SV_639	Enoplida	Trischistomatidae	Trischistoma	Trischistoma pellucidum	6e-165	100	592/592	KR492034	Predator	3	Enoplea	Enoplia	Triplonchida	Trischistoma pellucidum
R4_SV_687	Rhabditida	Rhabditidae	Distolabrellus	Distolabrellus veechi	1e-161	99.68	580/580	AF082999	Bacteria feeder	1	Chromadorea	NA	Rhabditida	NA
R4_SV_695	Mononchida	Mylonchulidae	Mylonchulus	Mylonchulus sigmaturus	0	100	750/750	AB361447 etc	Predator	4	Enoplea	Dorylaimia	Mononchida	Mylonchulus sp. JH-2004
R4_SV_712	Rhabditida	Tylenchidae	Discopersicus, Basiria	Discopersicus iranicus, Basiria duplexa	0	100	747/747	KM502981 etc	NA/Plant feeder	(−)/2	Chromadorea	NA	Rhabditida	Basiria duplexa

Note: See notes in S3 and S5 Tables.

[a]Sole hit species derived from a different phylum shared the same e-value with multiple hit species and is enclosed in parentheses.
