# Supplementary material for: Use of universal primers for the 18S ribosomal RNA gene and whole soil DNAs to reveal the taxonomic structures of soil nematodes by high-throughput amplicon sequencing
Source: PLoS One. 2021 Nov 15;16(11):e0259842. doi: 10.1371/journal.pone.0259842 (PMC8592498; doi:10.1371/journal.pone.0259842)
Supplement: S7 Table — (PDF) [file pone.0259842.s007.pdf]

S7 Table. Nematode-derived SVs from region U1 and their taxa and feeding types based on the BLASTN search and the SILVA database.

U1_SV	BLASTN data								Feeding type	cp group	SILVA taxonomic data			
	Order	Family	Genus	Top hit	E-value	% identity	Total score	Accession no.			D7	D8	D9	D10
U1_SV_1	Dorylaimida	Belondiridae	Dorylaimellus	Dorylaimellus virginianus*	1e-172	98.84	617/640	AY552969	Plant feeder	5	Enoplea	Dorylaimia	Dorylaimida	Tylencholaimus mirabilis
U1_SV_5	Triplonchida	Trichodoridae	Paratrichodorus	Paratrichodorus sp.	1e-177	100	634/634	MG938558 etc	Plant feeder	4	Enoplea	Enoplia	Triplonchida	Paratrichodorus porosus
U1_SV_6	Triplonchida	Prismatolaimidae	Prismatolaimus	Prismatolaimus cf. dolichurus JH-2004*	6e-175	99.71	625/628	AY284727	Bacteria feeder	3	Enoplea	Enoplia	Triplonchida	Ambiguous_taxa
U1_SV_10	Triplonchida	Diphtherophoridae	Diphtherophora	Diphtherophora obesus*	9e-119	89.8	438/634	KY119878 etc	Fungus feeder	3	Enoplea	Enoplia	Triplonchida	NA
U1_SV_16	Dorylaimida	Mydonomidae	Dorylaimoides	Dorylaimoides sp. WJW-2016	1e-177	99.71	634/634	KU662325	Fungus feeder	4	Enoplea	Dorylaimia	Dorylaimida	Nematoda environmental sample
U1_SV_31	Mononchida	Mylonchulidae	Mylonchulus	Mylonchulus sp.	3e-178	100	636/636	KJ778171 etc	Predator	4	Enoplea	Dorylaimia	Mononchida	Ambiguous_taxa
U1_SV_32	Rhabditida	Tylenchidae	Basiria	Basiria sp.*	1e-166	97.97	597/636	KJ869382 etc	Plant feeder	2	Chromadorea	NA	Rhabditida	NA
U1_SV_33	Triplonchida	Prismatolaimidae	Prismatolaimus	Prismatolaimus intermedius	1e-177	100	634/634	EU040133	Bacteria feeder	3	Enoplea	Enoplia	Triplonchida	Schizomidae environmental sample
U1_SV_34	Triplonchida	Prismatolaimidae	Prismatolaimus	Prismatolaimus cf. intermedius	1e-177	100	634/634	KJ636367 etc	Bacteria feeder	3	Enoplea	Enoplia	Triplonchida	Schizomidae environmental sample
U1_SV_35	Dorylaimida	Mydonomidae, Dorylaimidae	Dorylaimoides, Mesodorylaimus	Dorylaimoides sp. WJW-2016*, Mesodorylaimus cf. nigritulus AV-2005*	1e-157	96.24	568/584	KU66232 etc	Fungus feeder/Omnivore	4/4	Enoplea	Dorylaimia	Dorylaimida	NA
U1_SV_36	Dorylaimida	Belondiridae	Dorylaimellus	Dorylaimellus virginianus*	5e-171	98.55	612/634	AY552969	Plant feeder	5	Enoplea	Dorylaimia	Dorylaimida	Tylencholaimus mirabilis
U1_SV_39	Triplonchida	Diphtherophoridae	Diphtherophora	Diphtherophora obesus*	7e-125	90.94	459/632	KY119878	Fungus feeder	3	Enoplea	Enoplia	Triplonchida	NA
U1_SV_43	Rhabditida	Travassosinematidae	Travassosinema	Travassosinema sp. Ishigaki	2e-169	98.27	606/606	LC214832	Parasite	(−)	Chromadorea	NA	NA	NA
U1_SV_45	Triplonchida	Trichodoridae	Paratrichodorus	Paratrichodorus sp.	5e-176	99.71	628/628	MG938558 etc	Plant feeder	4	Enoplea	Enoplia	Triplonchida	Paratrichodorus porosus
U1_SV_50	Dorylaimida	Mydonomidae	Dorylaimoides	Dorylaimoides sp. WJW-2016*	5e-176	99.42	628/640	KU662325	Fungus feeder	4	Enoplea	Dorylaimia	Dorylaimida	Nematoda environmental sample
U1_SV_51	Triplonchida	Prismatolaimidae	Prismatolaimus	Prismatolaimus cf. dolichurus JH-2004*	3e-173	99.41	619/623	AY284727	Bacteria feeder	3	Enoplea	Enoplia	Triplonchida	Ambiguous_taxa
U1_SV_59	Plectida	Plectidae	Ceratoplectus	Ceratoplectus sp.	3e-178	100	636/636	MN082268 etc	Bacteria feeder	2	Chromadorea	NA	Araeolaimida	Plectus sp.
U1_SV_61	Dorylaimida	Mydonomidae	Dorylaimoides	Dorylaimoides sp. WJW-2016*	1e-172	98.84	617/640	KU662325	Fungus feeder	4	Enoplea	Dorylaimia	Dorylaimida	Nematoda environmental sample
U1_SV_69	Rhabditida	Travassosinematidae	Travassosinema	Travassosinema sp. Nago	2e-169	98.27	606/606	LC214829	Parasite	(−)	Chromadorea	NA	Rhabditida	NA
U1_SV_75	Triplonchida	Prismatolaimidae	Prismatolaimus	Prismatolaimus cf. dolichurus JH-2004*	8e-164	97.66	588/616	AY284727	Bacteria feeder	3	Enoplea	Enoplia	Triplonchida	Ambiguous_taxa
U1_SV_77	Dorylaimida	Mydonomidae, Dorylaimidae	Dorylaimoides, Mesodorylaimus	Dorylaimoides sp. WJW-2016*, Mesodorylaimus cf. nigritulus AV-2005*	2e-159	96.53	573/584	KU662325 etc	Fungus feeder/Omnivore	4/4	Enoplea	Dorylaimia	Dorylaimida	NA
U1_SV_80	Triplonchida	Diphtherophoridae	Diphtherophora	Diphtherophora obesus*	2e-140	93.59	510/634	AY552968	Fungus feeder	3	Enoplea	Enoplia	Triplonchida	NA
U1_SV_92	Dorylaimida	Belondiridae	Dorylaimellus	Dorylaimellus virginianus*	2e-169	98.27	606/628	AY552969	Plant feeder	5	Enoplea	Dorylaimia	Dorylaimida	Tylencholaimus mirabilis
U1_SV_98	Dorylaimida	Mydonomidae	Dorylaimoides	Dorylaimoides sp. WJW-2016	1e-177	99.71	634/634	KU662325	Fungus feeder	4	Enoplea	Dorylaimia	Dorylaimida	Nematoda environmental sample
U1_SV_117	Rhabditida	Tylenchidae	Basiria	Basiria sp.*	2e-164	97.67	590/628	KJ869382 etc	Plant feeder	2	Chromadorea	NA	Rhabditida	NA
U1_SV_128	Triplonchida	Diphtherophoridae	Diphtherophora	Diphtherophora obesus*	9e-114	88.92	422/595	AY552968	Fungus feeder	3	Enoplea	Enoplia	Triplonchida	NA
U1_SV_130	Rhabditida	Tylenchidae	Basiria	Basiria sp.*	2e-164	97.67	590/628	KJ869382 etc	Plant feeder	2	Chromadorea	NA	Rhabditida	NA
U1_SV_136	Rhabditida	Tylenchidae	Basiria	Basiria sp.*	5e-166	97.97	595/634	KJ869382 etc	Plant feeder	2	Chromadorea	NA	Rhabditida	NA
U1_SV_140	Rhabditida	Travassosinematidae	Travassosinema	Travassosinema sp. Ishigaki	1e-167	97.98	601/601	LC214832	Parasite	(−)	Chromadorea	NA	NA	NA

U1_SV_149	Rhabditida	Tylenchidae	Boleodorus	Boleodorus thylactus	2e-160	96.83	577/577	MK639397 etc	Plant feeder	2	Chromadorea	NA	Rhabditida	Boleodorus thylactus
U1_SV_152	Dorylaimida	Tylencholaimidae	Tylencholaimus	Tylencholaimus mirabilis*	2e-165	97.41	593/643	AY284835	Fungus feeder	4	Enoplea	Dorylaimia	Dorylaimida	NA
U1_SV_155	Chromadorida	Cyatholaimidae	Achromadora	Achromadora sp. JH-2004	4e-167	97.69	599/599	AY284718	Eucaryote feeder	3	Chromadorea	NA	Chromadorida	Achromadora sp. JH-2004
U1_SV_158	Rhabditida	Travassosinematidae	Travassosinema	Travassosinema sp. Nago	1e-167	97.98	601/601	LC214829	Parasite	(−)	Chromadorea	NA	Rhabditida	NA
U1_SV_161	Rhabditida	Travassosinematidae	Travassosinema	Travassosinema sp. Ishigaki	1e-167	97.98	601/601	LC214832	Parasite	(−)	Chromadorea	NA	NA	NA
U1_SV_188	Rhabditida	Tylenchidae	Basiria	Basiria sp.	5e-161	97.09	579/579	KJ869382 etc	Plant feeder	2	Chromadorea	NA	Rhabditida	Basiria duplexa
U1_SV_190	Rhabditida	Travassosinematidae	Travassosinema	Travassosinema sp. Ishigaki	4e-167	97.97	599/599	LC214832	Parasite	(−)	Chromadorea	NA	NA	NA
U1_SV_211	Triplonchida	Prismatolaimidae	Prismatolaimus	Prismatolaimus cf. dolichurus JH-2004*	5e-146	95.01	529/532	AY284727	Bacteria feeder	3	Enoplea	Enoplia	Triplonchida	Dintheria tenuissima
U1_SV_214	Rhabditida	Rhabditidae	Rhabditis	Rhabditis sp.	1e-177	100	634/634	LC275874 etc	Bacteria feeder	1	Chromadorea	NA	Rhabditida	Rhabditis sp. DF5059
U1_SV_235	Rhabditida	Tylenchulidae	Paratylenchus	Paratylenchus lepidus	1e-176	100	630/630	MK886695	Plant feeder	2	Chromadorea	NA	Rhabditida	Paratylenchus cf. neoamblicephalus
U1_SV_238	Dorylaimida	Leptonchidae	Proleptonchus	Proleptonchus weischeri	2e-164	97.4	590/590	KJ636399	Fungus feeder	4	Enoplea	Dorylaimia	Dorylaimida	NA
U1_SV_280	Rhabditida	Tylenchulidae	Paratylenchus	Paratylenchus lepidus	4e-177	100	632/632	MK886695	Plant feeder	2	Chromadorea	NA	Rhabditida	Paratylenchus cf. neoamblicephalus
U1_SV_292	Triplonchida	Prismatolaimidae	Prismatolaimus	Prismatolaimus sp.*	1e-162	97.38	584/612	KJ636367 etc	Bacteria feeder	3	Enoplea	Enoplia	Triplonchida	Phaseoleae environmental sample
U1_SV_343	Dorylaimida	Dorylaimidae, Actinolaimidae	Mesodorylaimus, Prodorylaimus, Paractinolaimus	Mesodorylaimus sp. 2 WJW-2018, Prodorylaimus sp., Paractinolaimus sp.	1e-172	98.84	617/617	MG921255 etc	Omnivore/Omnivore/Omnivore	4/4/5	Enoplea	Dorylaimia	Dorylaimida	NA
U1_SV_348	Dorylaimida	Belondiridae	Dorylaimellus	Dorylaimellus parvulus	3e-157	100	566/566	AY911968	Plant feeder	5	Chromadorea	NA	Rhabditida	Cactodera sp. WY-2012
U1_SV_362	Dorylaimida	Tylencholaimidae	Tylencholaimus	Tylencholaimus sp.	0	100	645/645	MG921285 etc	Fungus feeder	4	Enoplea	Dorylaimia	Dorylaimida	Tylencholaimus sp. n. WJW-2016
U1_SV_385	Rhabditida	Tylenchidae	Filenchus	Filenchus discrepans	4e-132	92.22	483/483	KJ869311 etc	Fungus feeder	2	Chromadorea	NA	Rhabditida	Filenchus discrepans
U1_SV_398	Dorylaimida	Qudsianematidae	Allodorylaimus, Thonus	Allodorylaimus sp., Thonus minutus	1e-177	99.71	634/634	KY942068 etc	Omnivore/Omnivore	4/4	Enoplea	Dorylaimia	Dorylaimida	NA
U1_SV_410	Enoplida	Trischistomatidae	Trischistoma	Trischistoma sp.	2e-180	100	643/643	KR492034 etc	Predator	3	Enoplea	Enoplia	Triplonchida	NA
U1_SV_415	Rhabditida	Tylenchidae	Boleodorus	Boleodorus thylactus	7e-160	96.82	575/575	MK639397 etc	Plant feeder	2	Chromadorea	NA	Rhabditida	Boleodorus thylactus
U1_SV_431	Dorylaimida	Belondiridae	Dorylaimellus	Dorylaimellus virginianus*	7e-180	97.84	641/665	AY552969	Plant feeder	5	Chromadorea	NA	Araeolaimida	Acrobeloides buetschlii
U1_SV_434	Rhabditida	Tylenchulidae	Paratylenchus	Paratylenchus lepidus	9e-84	95.17	530/530	MK886695	Plant feeder	2	Chromadorea	NA	Rhabditida	NA
U1_SV_470	Rhabditida	Aphelenchoididae	Aphelenchoides	Aphelenchoides sp. RH-2018	2e-160	99.07	577/577	MF070486	Fungus feeder	2	Chromadorea	NA	Rhabditida	metagenome

Note: See notes in S3 Table.
