# Supplementary material for: Use of universal primers for the 18S ribosomal RNA gene and whole soil DNAs to reveal the taxonomic structures of soil nematodes by high-throughput amplicon sequencing
Source: PLoS One. 2021 Nov 15;16(11):e0259842. doi: 10.1371/journal.pone.0259842 (PMC8592498; doi:10.1371/journal.pone.0259842)
Supplement: S8 Table — (PDF) [file pone.0259842.s008.pdf]

S8 Table. Nematode-derived SVs from region U2 and their taxa and feeding types based on the BLASTN search and the SILVA database.

U2_SV	BLASTN data								Feeding type	cp group	SILVA taxonomic data			
	Order	Family	Genus	Top hit	E-value	% identity	Total score	Accession no.			D7	D8	D9	D10
U2_SV_1	Dorylaimida	Belondiridae	Dorylaimellus	Dorylaimellus parvulus	0	100	763/763	AY911968	Plant feeder	5	Enoplea	Dorylaimia	Dorylaimida	NA
U2_SV_4	Triplonchida	Trichodoridae	Paratrichodorus	Paratrichodorus porosus	0	100	763/763	MG9385714 etc	Plant feeder	4	Enoplea	Enoplia	Triplonchida	Paratrichodorus porosus
U2_SV_5	Triplonchida	Prismatolaimidae	Prismatolaimus	Prismatolaimus sp.	0	100	763/763	LC186686 etc	Bacteria feeder	3	Enoplea	Enoplia	Triplonchida	Ambiguous_taxa
U2_SV_9	Rhabditida	Tylenchidae	Discopersicus, Basiria	Discopersicus iranicus, Basiria sp.	0	100	761/761	KM502981 etc	NA/Plant feeder	(−)/2	Chromadorea	NA	Rhabditida	Basiria duplexa
U2_SV_10	Rhabditida	Cosmocercidae, Thelastomatidae	Cosmocerca, Cephalobellus	Cosmocerca simile*, Cephalobellus brevicaudatus*	0	99.51	750/761	MN839758 etc	Parasite	(−)	Chromadorea	NA	Rhabditida	Ambiguous_taxa
U2_SV_15	Triplonchida	Diphtherophoridae	Diphtherophora	Diphtherophora sp. Shahrood	3e-169	93.24	606/606	KY115102	Fungus feeder	3	NA	NA	NA	NA
U2_SV_18	Mononchida	Mylonchulidae	Mylonchulus	Mylonchulus sigmaturus	0	100	765/765	AB361447 etc	Predator	4	Enoplea	Dorylaimia	Mononchida	Mylonchulus sp. JH-2004
U2_SV_21	Dorylaimida	Mydonomidae, Dorylaimidae	Dorylaimoides, Thornenema	Dorylaimoides sp., Thornenema cf. laevicapitatum	0	99.76	758/758	KU662325 etc	Fungus feeder/Omnivore	4/5	Enoplea	Dorylaimia	Dorylaimida	Ambiguous_taxa
U2_SV_24	Triplonchida	Prismatolaimidae	Prismatolaimus	Prismatolaimus sp.	0	100	763/763	LC186851 etc	Bacteria feeder	3	Enoplea	Enoplia	Triplonchida	Ambiguous_taxa
U2_SV_27	Rhabditida, (Triplonchida)	Cephalobidae, (Diphtherophoridae)	Acrobeloides, Cephalobus, (Tylolaimophorus)	Acrobeloides sp., Cephalobus sp., (Tylolaimophorus sp.)	0	100	763/763	MK636581 etc	Bacteria feeder/Bacteria feeder	2/2	Chromadorea	NA	Rhabditida	NA
U2_SV_30	Dorylaimida	Leptonchidae	Leptonchus	Leptonchus microdens	0	97.11	699/699	AY146539	Fungus feeder	4	Enoplea	Dorylaimia	Dorylaimida	NA
U2_SV_37	Rhabditida	Cosmocercidae, Thelastomatidae	Cosmocerca, Cephalobellus	Cosmocerca simile*, Cephalobellus brevicaudatus*	0	99.27	745/756	MN839758 etc	Parasite	(−)	Chromadorea	NA	Rhabditida	Ambiguous_taxa
U2_SV_38	Dorylaimida	Belondiridae	Dorylaimellus	Dorylaimellus parvulus	0	99.76	758/758	AY911968	Plant feeder	5	Enoplea	Dorylaimia	Dorylaimida	NA
U2_SV_40	Triplonchida	Diphtherophoridae	Diphtherophora	Diphtherophora sp.*	2e-180	94.69	643/686	KY115102 etc	Fungus feeder	3	Enoplea	Enoplia	Triplonchida	NA
U2_SV_45	Plectida, (Rhabditida)	Plectidae, (Cephalobidae)	Plectus, Wilsonema, (Acrobeloides)	Plectus sp., Wilsonema sp., (Acrobeloides sp.)	0	100	763/763	LC186814 etc	Bacteria feeder/Bacteria feeder	2/2	Chromadorea	NA	Araeolaimida	Acrobeloides buetschlii
U2_SV_49	Triplonchida	Trichodoridae	Paratrichodorus	Paratrichodorus porosus	0	99.76	758/758	MG938571 etc	Plant feeder	4	Enoplea	Enoplia	Triplonchida	Paratrichodorus porosus
U2_SV_51	Dorylaimida	Mydonomidae, Dorylaimidae	Dorylaimoides, Thornenema	Dorylaimoides sp., Thornenema cf. laevicapitatum	0	99.76	758/758	KU662325 etc	Fungus feeder/Omnivore	4/5	Enoplea	Dorylaimia	Dorylaimida	Ambiguous_taxa
U2_SV_56	Triplonchida	Prismatolaimidae	Prismatolaimus	Prismatolaimus sp.	0	99.76	758/758	LC186686 etc	Bacteria feeder	3	Enoplea	Enoplia	Triplonchida	Ambiguous_taxa
U2_SV_61	Dorylaimida	Mydonomidae, Dorylaimidae	Dorylaimoides, Thornenema	Dorylaimoides sp., Thornenema cf. laevicapitatum	0	99.52	752/752	KU662325 etc	Fungus feeder/Omnivore	4/5	Enoplea	Dorylaimia	Dorylaimida	Ambiguous_taxa
U2_SV_63	Triplonchida	Trichodoridae	Paratrichodorus	Paratrichodorus porosus	0	99.76	758/758	MG938571 etc	Plant feeder	4	Enoplea	Enoplia	Triplonchida	Paratrichodorus porosus
U2_SV_72	Dorylaimida	Belondiridae	Dorylaimellus	Dorylaimellus parvulus	0	99.76	758/758	AY911968	Plant feeder	5	Enoplea	Dorylaimia	Dorylaimida	NA
U2_SV_82	Rhabditida	Tylenchidae	Boleodorus	Boleodorus cf. thylactus TSH-2005	0	97.89	734/734	AY911926	Plant feeder	2	Chromadorea	NA	Rhabditida	Boleodorus thylactus
U2_SV_85	Triplonchida	Prismatolaimidae	Prismatolaimus	Prismatolaimus sp.	0	99.76	758/758	LC186686 etc	Bacteria feeder	3	Enoplea	Enoplia	Triplonchida	Ambiguous_taxa
U2_SV_88	Rhabditida	Tylenchidae	Discopersicus	Discopersicus iranicus	0	99.76	756/756	KM502981	*Plant feeder*	*2*	Chromadorea	NA	Rhabditida	Basiria duplexa
U2_SV_89	Rhabditida	Tylenchidae	Basiria	Basiria cf. obliquua TSH-2005	0	98.3	721/721	AY911919	Plant feeder	2	Chromadorea	NA	Rhabditida	Neopsilenchus magnidens
U2_SV_90	Triplonchida	Prismatolaimidae	Prismatolaimus	Prismatolaimus sp.	0	99.76	758/758	LC186686 etc	Bacteria feeder	3	Enoplea	Enoplia	Triplonchida	Ambiguous_taxa
U2_SV_96	Rhabditida	Tylenchidae	Discopersicus	Discopersicus iranicus	0	99.76	756/756	KM502981	*Plant feeder*	*2*	Chromadorea	NA	Rhabditida	Basiria duplexa
U2_SV_99	Rhabditida	Tylenchulidae	Paratylenchus	Paratylenchus lepidus	0	100	761/761	MK886695	Plant feeder	2	Chromadorea	NA	Rhabditida	NA

U2_SV_101	Triplonchida	Trichodoridae	Paratrichodorus	Paratrichodorus sp.	0	100	758/758	MG938571 etc	Plant feeder	4	Enoplea	Enoplia	Triplonchida	Paratrichodorus porosus
U2_SV_103	Triplonchida	Diphtherophoridae	Diphtherophora	Diphtherophora sp. Shahrood	1e-167	93	601/601	KY115102	Fungus feeder	3	Chromadorea	NA	NA	NA
U2_SV_105	Dorylaimida	Belondiridae	Dorylaimellus	Dorylaimellus parvulus	0	99.76	758/758	AY911968	Plant feeder	5	Enoplea	Dorylaimia	Dorylaimida	NA
U2_SV_110	Mononchida	Mylonchulidae	Mylonchulus	Mylonchulus sigmaturus	0	99.76	760/760	AB361447 etc	Predator	4	Enoplea	Dorylaimia	Mononchida	Mylonchulus sp. JH-2004
U2_SV_113	Rhabditida	Cosmocercidae, Thelastomatidae	Cosmocerca, Cephalobellus	Cosmocerca simile*, Cephalobellus brevicaudatus*	0	99.27	745/756	MN839758 etc	Parasite	(−)	Chromadorea	NA	Rhabditida	Ambiguous_taxa
U2_SV_114	Triplonchida	Trichodoridae	Paratrichodorus	Paratrichodorus porosus	0	99.76	758/758	MG938571 etc	Plant feeder	4	Enoplea	Enoplia	Triplonchida	Paratrichodorus porosus
U2_SV_124	Triplonchida	Trichodoridae	Paratrichodorus	Paratrichodorus porosus	0	99.76	758/758	MG938571 etc	Plant feeder	4	Enoplea	Enoplia	Triplonchida	Paratrichodorus porosus
U2_SV_127	Dorylaimida	Mydonomidae	Dorylaimoides	Dorylaimoides sp.*	0	99.03	758/758	KU662325 etc	Fungus feeder	4	Enoplea	Dorylaimia	Dorylaimida	NA
U2_SV_133	Dorylaimida	Belondiridae	Dorylaimellus	Dorylaimellus parvulus	0	99.76	758/758	AY911968	Plant feeder	5	Enoplea	Dorylaimia	Dorylaimida	NA
U2_SV_134	Rhabditida	Cosmocercidae, Thelastomatidae	Cosmocerca, Cephalobellus	Cosmocerca simile*, Cephalobellus brevicaudatus*	0	99.27	745/756	MN839758 etc	Parasite	(−)	Chromadorea	NA	Rhabditida	Ambiguous_taxa
U2_SV_135	Mononchida	Mylonchulidae	Mylonchulus	Mylonchulus sigmaturus	0	99.76	760/760	AB361447 etc	Predator	4	Enoplea	Dorylaimia	Mononchida	Mylonchulus sp. JH-2004
U2_SV_136	Rhabditida	Tylenchidae	Discopersicus	Discopersicus iranicus	0	99.76	756/756	KM502981	*Plant feeder*	*2*	Chromadorea	NA	Rhabditida	Basiria duplexa
U2_SV_138	Rhabditida	Tylenchidae	Coslenchus	Coslenchus sp.	0	100	761/761	MN542199 etc	Plant feeder	2	Chromadorea	NA	Rhabditida	NA
U2_SV_143	Triplonchida	Diphtherophoridae	Diphtherophora	Diphtherophora sp. 803S-002*	0	96.34	673/695	EU880005	Fungus feeder	3	Enoplea	Enoplia	Triplonchida	NA
U2_SV_155	Dorylaimida	Belondiridae	Dorylaimellus	Dorylaimellus parvulus	0	99.76	758/758	AY911968	Plant feeder	5	Enoplea	Dorylaimia	Dorylaimida	NA
U2_SV_157	Rhabditida	Cosmocercidae, Thelastomatidae	Cosmocerca, Cephalobellus	Cosmocerca simile*, Cephalobellus brevicaudatus*	0	99.27	745/756	MN839758 etc	Parasite	(−)	Chromadorea	NA	Rhabditida	Ambiguous_taxa
U2_SV_160	Triplonchida	Trichodoridae	Paratrichodorus	Paratrichodorus porosus	0	99.76	758/758	MG938571 etc	Plant feeder	4	Enoplea	Enoplia	Triplonchida	Paratrichodorus porosus
U2_SV_161	Rhabditida	Tylenchidae	Malenchus	Malenchus sp.	8e-135	88.32	492/492	LC186800 etc	Plant feeder	2	Chromadorea	NA	Rhabditida	NA
U2_SV_162	Chromadorida	Cyatholaimidae	Achromadora	Achromadora sp. JH-2004	0	100	765/765	AY284717	Eucaryote feeder	3	Chromadorea	NA	Chromadorida	Achromadora sp. JH-2004
U2_SV_172	Triplonchida	Diphtherophoridae	Diphtherophora	Diphtherophora sp. Shahrood	1e-167	93	601/601	KY115102	Fungus feeder	3	NA	NA	NA	NA
U2_SV_181	Triplonchida	Diphtherophoridae	Diphtherophora	Diphtherophora sp. Shahrood	2e-171	93.49	614/614	KY115102	Fungus feeder	3	Enoplea	Enoplia	Triplonchida	NA
U2_SV_182	Rhabditida, (Triplonchida)	Cephalobidae, (Diphtherophoridae)	Acrobeloides, Cephalobus, (Tylolaimophorus)	Acrobeloides sp., Cephalobus sp., (Tylolaimophorus sp.)	0	99.76	758/758	MK636581 etc	Bacteria feeder/Bacteria feeder	2/2	Chromadorea	NA	Rhabditida	NA
U2_SV_189	Dorylaimida	Belondiridae	Dorylaimellus	Dorylaimellus parvulus	0	99.76	758/758	AY911968	Plant feeder	5	Enoplea	Dorylaimia	Dorylaimida	NA
U2_SV_191	Rhabditida	Cosmocercidae, Thelastomatidae	Cosmocerca, Cephalobellus	Cosmocerca simile*, Cephalobellus brevicaudatus*	0	99.27	745/756	MN839758 etc	Parasite	(−)	Chromadorea	NA	Rhabditida	Ambiguous_taxa
U2_SV_192	Dorylaimida	Belondiridae	Dorylaimellus	Dorylaimellus parvulus	0	99.76	758/758	AY911968	Plant feeder	5	Enoplea	Dorylaimia	Dorylaimida	NA
U2_SV_193	Monhysterida	Monhysteridae	Eumonhystera	Eumonhystera sp.*	0	98.8	739/750	KJ636251 etc	Bacteria feeder	3	Chromadorea	NA	Monhysterida	NA
U2_SV_194	Triplonchida	Prismatolaimidae	Prismatolaimus	Prismatolaimus sp.	0	99.76	758/758	LC186686 etc	Bacteria feeder	3	Enoplea	Enoplia	Triplonchida	Ambiguous_taxa
U2_SV_198	Triplonchida	Trichodoridae	Paratrichodorus	Paratrichodorus porosus	0	99.76	758/758	MG938571 etc	Plant feeder	4	Enoplea	Enoplia	Triplonchida	Paratrichodorus porosus
U2_SV_209	Rhabditida	Cosmocercidae, Thelastomatidae	Cosmocerca, Cephalobellus	Cosmocerca simile*, Cephalobellus brevicaudatus*	0	99.27	745/756	MN839758 etc	Parasite	(−)	Chromadorea	NA	NA	NA
U2_SV_214	Triplonchida	Trichodoridae	Paratrichodorus	Paratrichodorus porosus	0	99.76	758/758	MG938571 etc	Plant feeder	4	Enoplea	Enoplia	Triplonchida	Paratrichodorus porosus
U2_SV_216	Triplonchida	Prismatolaimidae	Prismatolaimus	Prismatolaimus sp.	0	99.76	758/758	LC186686 etc	Bacteria feeder	3	Enoplea	Enoplia	Triplonchida	Ambiguous_taxa
U2_SV_227	Rhabditida	Cosmocercidae, Thelastomatidae	Cosmocerca, Cephalobellus	Cosmocerca simile*, Cephalobellus brevicaudatus*	0	99.27	745/756	MN839758 etc	Parasite	(−)	Chromadorea	NA	Rhabditida	Ambiguous_taxa
U2_SV_233	Dorylaimida	Belondiridae	Dorylaimellus	Dorylaimellus parvulus	0	99.76	758/758	AY911968	Plant feeder	5	Enoplea	Dorylaimia	Dorylaimida	NA

U2_SV_234	Triplonchida	Diphtherophoridae	Diphtherophora	Diphtherophora sp. Shahrood	1e-167	93	601/601	KY115102	Fungus feeder	3	NA	NA	NA	NA
U2_SV_246	Triplonchida	Diphtherophoridae	Diphtherophora	Diphtherophora sp. Shahrood*	3e-169	93.24	606/608	KY115102	Fungus feeder	3	NA	NA	NA	NA
U2_SV_253	Triplonchida	Trichodoridae	Paratrichodorus	Paratrichodorus porosus	0	99.76	758/758	MG938571 etc	Plant feeder	4	Enoplea	Enoplia	Triplonchida	Paratrichodorus porosus
U2_SV_260	Dorylaimida	Belondiridae	Dorylaimellus	Dorylaimellus parvulus	0	99.76	758/758	AY911968	Plant feeder	5	Enoplea	Dorylaimia	Dorylaimida	NA
U2_SV_276	Rhabditida	Cosmocercidae, Thelastomatidae	Cosmocerca, Cephalobellus	Cosmocerca simile*, Cephalobellus brevicaudatus*	0	99.27	745/756	MN839758 etc	Parasite	(−)	Chromadorea	NA	Rhabditida	Ambiguous_taxa
U2_SV_277	Triplonchida	Prismatolaimidae	Prismatolaimus	Prismatolaimus sp.	0	99.76	758/758	LC186686 etc	Bacteria feeder	3	Enoplea	Enoplia	Triplonchida	Ambiguous_taxa
U2_SV_279	Rhabditida, (Triplonchida)	Cephalobidae, (Diphtherophoridae)	Acrobeloides, Cephalobus, (Tylolaimophorus)	Acrobeloides sp., Cephalobus sp., (Tylolaimophorus sp.)	0	99.76	758/758	MK636581 etc	Bacteria feeder/Bacteria feeder	2/2	Chromadorea	NA	Rhabditida	NA
U2_SV_280	Rhabditida	Cosmocercidae, Thelastomatidae	Cosmocerca, Cephalobellus	Cosmocerca simile*, Cephalobellus brevicaudatus*	0	99.27	745/756	MN839758 etc	Parasite	(−)	Chromadorea	NA	Rhabditida	Ambiguous_taxa
U2_SV_285	Rhabditida	Tylenchidae	Discopersicus	Discopersicus iranicus	0	99.76	756/756	KM502981	*Plant feeder*	*2*	Chromadorea	NA	Rhabditida	Basiria duplexa
U2_SV_286	Triplonchida	Trichodoridae	Paratrichodorus	Paratrichodorus porosus	0	99.76	758/758	MG938571 etc	Plant feeder	4	Enoplea	Enoplia	Triplonchida	Paratrichodorus porosus
U2_SV_287	Rhabditida	Cosmocercidae, Thelastomatidae	Cosmocerca, Cephalobellus	Cosmocerca simile*, Cephalobellus brevicaudatus*	0	99.27	745/756	MN839758 etc	Parasite	(−)	Chromadorea	NA	Rhabditida	Ambiguous_taxa
U2_SV_293	Dorylaimida	Tylencholaimidae	Tylencholaimus	Tylencholaimus sp.	0	98.8	739/739	LC186596 etc	Fungus feeder	4	Enoplea	Dorylaimia	Dorylaimida	NA
U2_SV_298	Triplonchida	Prismatolaimidae	Prismatolaimus	Prismatolaimus sp.	0	99.76	758/758	LC186851 etc	Bacteria feeder	3	Enoplea	Enoplia	Triplonchida	Ambiguous_taxa
U2_SV_301	Rhabditida	Tylenchidae	Discopersicus	Discopersicus iranicus	0	99.76	756/756	KM502981	*Plant feeder*	*2*	Chromadorea	NA	Rhabditida	Basiria duplexa
U2_SV_312	Monhysterida?	Monhysteridae	Eumonhystera	Eumonhystera filiformis*	0	99.52	730/741	AY593937 etc	Bacteria feeder	3	Chromadorea	NA	Monhysterida	NA
U2_SV_323	Triplonchida	Diphtherophoridae	Diphtherophora	Diphtherophora sp. Shahrood*	1e-167	92.98	601/603	KY115102	Fungus feeder	3	NA	NA	NA	NA
U2_SV_332	Triplonchida	Prismatolaimidae	Prismatolaimus	Prismatolaimus sp.	0	99.76	758/758	LC186851 etc	Bacteria feeder	3	Enoplea	Enoplia	Triplonchida	Ambiguous_taxa
U2_SV_334	Plectida, (Rhabditida)	Plectidae, (Cephalobidae)	Plectus, Wilsonema, (Acrobeloides)	Plectus sp., Wilsonema sp., (Acrobeloides sp.)	0	99.76	758/758	LC186814 etc	Bacteria feeder/Bacteria feeder	2/2	Chromadorea	NA	Araeolaimida	Acrobeloides buetschlii
U2_SV_336	Mononchida	Mylonchulidae	Mylonchulus	Mylonchulus sigmaturus	0	99.76	760/760	AB361447 etc	Predator	4	Enoplea	Dorylaimia	Mononchida	Mylonchulus sp. JH-2004
U2_SV_361	Dorylaimida	Mydonomidae, Dorylaimidae	Dorylaimoides, Thornenema	Dorylaimoides sp., Thornenema cf. laevicapitatum	0	99.52	752/752	KU662325 etc	Fungus feeder/Omnivore	4/5	Enoplea	Dorylaimia	Dorylaimida	Ambiguous_taxa
U2_SV_379	Rhabditida	Cosmocercidae, Thelastomatidae	Cosmocerca, Cephalobellus	Cosmocerca simile*, Cephalobellus brevicaudatus*	0	99.27	745/756	MN839758 etc	Parasite	(−)	Chromadorea	NA	Rhabditida	Ambiguous_taxa
U2_SV_420	Rhabditida	Cosmocercidae, Thelastomatidae	Cosmocerca, Cephalobellus	Cosmocerca simile*, Cephalobellus brevicaudatus*	0	99.54	798/809	MN839758 etc	Parasite	(−)	Chromadorea	NA	Rhabditida	Ambiguous_taxa
U2_SV_432	Rhabditida	Ecphyadophoridae	Lelenchus	Lelenchus sp. MB-2019*	5e-157	91.71	566/699	MN542204	Plant feeder	2	Chromadorea	NA	Rhabditida	NA
U2_SV_449	Triplonchida	Prismatolaimidae	Prismatolaimus	Prismatolaimus sp.	0	100	763/763	LC186858 etc	Bacteria feeder	3	Enoplea	Enoplia	Triplonchida	Ambiguous_taxa
U2_SV_453	Monhysterida?	Monhysteridae	Eumonhystera	Eumonhystera filiformis*	0	99.52	752/763	AY593937 etc	Bacteria feeder	3	Chromadorea	NA	Monhysterida	Paralamyctes environmental sample
U2_SV_454	Dorylaimida	Aporcelaimidae	Aporcella	Aporcella vitrinus*	0	97.34	702/708	MG921235 etc	Omnivore	5	Enoplea	Dorylaimia	Dorylaimida	NA
U2_SV_492	Rhabditida	Cephalobidae	Eucephalobus, Cephalobus	Eucephalobus laevis, Cephalobus sp.	0	100	763/763	AY911991 etc	Bacteria feeder/Bacteria feeder	2/2	Chromadorea	NA	Rhabditida	NA
U2_SV_548	Rhabditida	Aphelenchoididae	Aphelenchoides	Aphelenchoides sp.*	0	95.19	649/651	KY769066 etc	Fungus feeder	2	Chromadorea	NA	Rhabditida	NA
U2_SV_597	Rhabditida	Tylenchidae	Boleodorus	Boleodorus cf. thylactus TSH-2005	0	96.96	712/712	AY911926	Plant feeder	2	Chromadorea	NA	Rhabditida	Neopsilenchus magnidens

Note: See notes in S3, S5, and S6 Tables.
