# Supplementary material for: Use of universal primers for the 18S ribosomal RNA gene and whole soil DNAs to reveal the taxonomic structures of soil nematodes by high-throughput amplicon sequencing
Source: PLoS One. 2021 Nov 15;16(11):e0259842. doi: 10.1371/journal.pone.0259842 (PMC8592498; doi:10.1371/journal.pone.0259842)
Supplement: S9 Table — (PDF) [file pone.0259842.s009.pdf]

**S9 Table. Numbers of regional nematode SVs by nematode family and colonizer–persister (cp) value.**

| cp-value | Family | | | | | | | | | | | | |
|---|---|---|---|---|---|---|---|---|---|---|---|---|---|
| Template DNA for PCR | | Nematode genomic DNA | | | | | | Soil DNA | | | | | |
| Regions | | R1 | R2 | R3 | R4 | U1 | U2 | R1 | R2 | R3 | R4 | U1 | U2 |
| 1 | Rhabditidae | 0 | 0 | 0 | 1 | 0 | 0 | 1 | 1 | 1 | 1 | 1 | 0 |
| 2 | Anguinidae | 0 | 0 | 0 | 0 | 0 | 0 | 0 | 1 | 0 | 0 | 0 | 0 |
| 2 | Aphelenchoididae | 0 | 0 | 0 | 0 | 1 | 0 | 0 | 1 | 1 | 2 | 0 | 1 |
| 2 | Cephalobidae | 2 | 1 | 3 | 3 | 0 | 3 | 1 | 0 | 1 | 2 | 0 | 2 |
| 2 | Ecphyadophoridae | 0 | 0 | 0 | 0 | 0 | 0 | 0 | 0 | 0 | 0 | 0 | 1 |
| 2 | Monhysteridae | 0 | 0 | 0 | 0 | 0 | 0 | 3 | 2 | 6 | 4 | 0 | 3 |
| 2 | Plectidae | 1 | 1 | 1 | 1 | 1 | 2 | 1 | 2 | 1 | 1 | 1 | 1 |
| 2 | Tylenchidae | 6 | 8 | 6 | 8 | 5 | 10 | 6 | 9 | 7 | 6 | 3 | 4 |
| 2 | Tylenchulidae | 1 | 1 | 1 | 1 | 2 | 1 | 1 | 1 | 1 | 1 | 1 | 1 |
| 3 | Cyatholaimidae | 1 | 1 | 1 | 1 | 0 | 1 | 1 | 1 | 4 | 1 | 1 | 1 |
| 3 | Diphtherophoridae | 3 | 6 | 1 | 5 | 4 | 9 | 1 | 2 | 1 | 3 | 3 | 2 |
| 3 | Meloidogynidae | 0 | 0 | 0 | 0 | 0 | 0 | 0 | 0 | 0 | 1 | 0 | 0 |
| 3 | Odontolaimidae | 1 | 0 | 1 | 0 | 0 | 0 | 3 | 1 | 1 | 0 | 0 | 0 |
| 3 | Prismatolaimidae | 4 | 7 | 2 | 5 | 5 | 9 | 3 | 4 | 3 | 2 | 5 | 6 |
| 3 | Trischistomatidae | 0 | 1 | 0 | 1 | 1 | 0 | 0 | 0 | 0 | 0 | 0 | 0 |
| 4 | Alaimidae | 0 | 0 | 0 | 0 | 0 | 0 | 1 | 0 | 1 | 1 | 0 | 0 |
| 4 | Leptonchidae | 0 | 0 | 0 | 1 | 0 | 1 | 0 | 1 | 0 | 1 | 1 | 1 |
| 4 | Mydonomidae | 1 | 4 | 0 | 1 | 4 | 1 | 0 | 0 | 0 | 0 | 1 | 0 |
| 4 | Mylonchulidae | 1 | 1 | 1 | 2 | 1 | 4 | 1 | 1 | 1 | 1 | 1 | 1 |
| 4 | Qudsianematidae | 0 | 0 | 0 | 0 | 0 | 0 | 0 | 0 | 1 | 0 | 1 | 0 |
| 4 | Trichodoridae | 2 | 3 | 2 | 3 | 2 | 11 | 1 | 1 | 2 | 1 | 1 | 2 |
| 4 | Tylencholaimidae | 0 | 1 | 0 | 0 | 0 | 0 | 1 | 2 | 1 | 2 | 2 | 1 |
| 5 | Aporcelaimidae | 0 | 0 | 0 | 0 | 0 | 0 | 0 | 0 | 0 | 1 | 0 | 1 |
| 5 | Belondiridae | 2 | 2 | 2 | 3 | 5 | 10 | 1 | 1 | 1 | 2 | 1 | 2 |
| 5 | Nygolaimidae | 0 | 0 | 0 | 0 | 0 | 0 | 0 | 0 | 1 | 0 | 0 | 0 |
| - | Thelastomatidae | 2 | 0 | 3 | 0 | 0 | 0 | 0 | 0 | 0 | 0 | 0 | 0 |
| - | Travassosinematidae | 0 | 7 | 0 | 0 | 6 | 0 | 0 | 0 | 0 | 0 | 0 | 0 |
| - | NA | 5 | 3 | 2 | 8 | 2 | 17 | 3 | 3 | 6 | 2 | 2 | 1 |
| Total numbers of SVs | | 32 | 47 | 26 | 44 | 39 | 79 | 29 | 34 | 41 | 35 | 25 | 31 |
| Total numbers of families detected | | 13 | 14 | 12 | 14 | 12 | 12 | 15 | 17 | 18 | 18 | 14 | 16 |

Columns with no SVs and one SV detected across six regions from each DNA sample are indicated by yellow and pale green colors. NA: not assigned. This category (NA) includes the SVs assigned to multiple families.
