# Supplementary material for: Use of universal primers for the 18S ribosomal RNA gene and whole soil DNAs to reveal the taxonomic structures of soil nematodes by high-throughput amplicon sequencing
Source: PLoS One. 2021 Nov 15;16(11):e0259842. doi: 10.1371/journal.pone.0259842 (PMC8592498; doi:10.1371/journal.pone.0259842)
Supplement: S10 Table — (PDF) [file pone.0259842.s010.pdf]

**S10 Table. Nematode-derived SVs from the agricultural soils (region U2) and their taxa and feeding types based on the BLASTN search and the SILVA database.**

| U2_SV(H03S) | BLASTN data | | | | | | | | Feeding type | cp group | SILVA taxonomic data | | | |
|---|---|---|---|---|---|---|---|---|---|---|---|---|---|---|
| | Order | Family | Genus | Top hit | E-value | % identity | Total score | Accession no. | | | D7 | D8 | D9 | D10 |
| U2_SV_11 | Triplonchida | Prismatolaimidae | Prismatolaimus | Prismatolaimus sp.* | 0 | 98.55 | 730/765 | LC186858 etc | Bacteria feeder | 3 | Enoplea | Enoplia | Triplonchida | Ambiguous_taxa |
| U2_SV_15 | Triplonchida | Prismatolaimidae | Prismatolaimus | Prismatolaimus sp. | 0 | 100 | 763/763 | LC186851 etc | Bacteria feeder | 3 | Enoplea | Enoplia | Triplonchida | Ambiguous_taxa |
| U2_SV_40 | Chromadorida | Cyatholaimidae | Achromadora | Achromadora sp. | 0 | 100 | 765/765 | LC186707 etc | Eukaryote feeder | 3 | Chromadorea | NA | Chromadorida | NA |
| U2_SV_47 | Rhabditida, (Triplonchida)[c] | Cephalobidae, (Diphtherophoridae)[c] | Acrobeloides, Cephalobus, (Tylolaimophorus)[c] | Acrobeloides sp., Cephalobus sp., (Tylolaimophorus sp.)[c] | 0 | 100 | 763/763 | MK636581 etc | Bacteria feeder/Bacteria feeder | 2/2 | Chromadorea | NA | Rhabditida | NA |
| U2_SV_49 | Dorylaimida | Qudsianematidae, Actinolaimidae, Dorylaimidae, Aporcelaimidae | Amblydorylaimus, Paractinolaimus, Mesodorylaimus, Thornenema, Metaporcelaimus | Amblydorylaimus isokaryon*, Paractinolaimus sahandi*, Mesodorylaimus sp.*, Thornenema sp.*, Metaporcelaimus conoidus* | 0 | 99.27 | 747/752 | KM092519 etc | NA/Omnivore/Omnivore/Omnivore/Omnivore | (−)/5/4/5/5 | Enoplea | Dorylaimia | Dorylaimida | Ambiguous_taxa |
| U2_SV_62 | Dorylaimida | Tylencholaimidae | Tylencholaimus, Capilonchus | Tylencholaimus sp.*, Capilonchus sp.* | 0 | 97.83 | 715/747 | AJ966510 etc | Fungus feeder/NA | 4/(−) | Enoplea | Dorylaimia | Dorylaimida | NA |
| U2_SV_80 | Chromadorida | Cyatholaimidae | Achromadora | Achromadora sp.* | 0 | 99.76 | 760/765 | LC186707 etc | Eukaryote feeder | 3 | Chromadorea | NA | Chromadorida | Prodesmodora circulata |
| U2_SV_101 | Plectida, (Rhabditida) | Plectidae, (Cephalobidae) | Plectus, (Acrobeloides) | Plectus sp., (Acrobeloides buetschlii) | 0 | 100 | 763/763 | LC186814 etc | Bacteria feeder | 2 | Chromadorea | NA | Araeolaimida | Acrobeloides buetschlii |
| U2_SV_102 | Rhabditida | Cephalobidae | Cephalobus | Cephalobus cubaensis | 0 | 99.28 | 758/758 | AF202161 | Bacteria feeder | 2 | Chromadorea | NA | Rhabditida | Cephalobus cubaensis |
| U2_SV_111 | Dorylaimida | Nygolaimidae | Clavicaudoides, Aquatides | Clavicaudoides sp., Aquatides christei | 0 | 100 | 769/769 | AY552967 etc | Predator/Predator | 5/5 | Enoplea | Dorylaimia | Dorylaimida | Aquatides aquaticus |
| U2_SV_116 | Triplonchida | Prismatolaimidae | Prismatolaimus | Prismatolaimus sp.* | 0 | 98.08 | 721/739 | LC186858 etc | Bacteria feeder | 3 | Enoplea | Enoplia | Triplonchida | Ambiguous_taxa |
| U2_SV_139 | Monhysterida | Monhysteridae | Eumonhystera | Eumonhystera filiformis* | 0 | 98.55 | 730/741 | AY593937 etc | Bacteria feeder | 2 | Chromadorea | NA | Monhysterida | NA |
| U2_SV_142 | Monhysterida | Monhysteridae | Eumonhystera | Eumonhystera cf. hungarica 1 JH-2014* | 3e-174 | 93.78 | 623/756 | KJ636237 | Bacteria feeder | 2 | Chromadorea | NA | Monhysterida | NA |
| U2_SV_155 | Rhabditida | Tylenchidae | Filenchus | Filenchus sp. | 0 | 99.76 | 761/761 | KJ869311 etc | Fungus feeder | 2 | Chromadorea | NA | Rhabditida | Filenchus discrepans |
| U2_SV_159 | Monhysterida | Monhysteridae | Eumonhystera | Eumonhystera filiformis* | 0 | 97.58 | 708/725 | AY593937 etc | Bacteria feeder | 2 | Chromadorea | NA | Monhysterida | NA |
| U2_SV_160 | Rhabditida | Aphelenchidae | Aphelenchus | Aphelenchus sp. | 0 | 100 | 760/760 | KX356730 etc | Fungus feeder | 2 | Chromadorea | NA | Rhabditida | Aphelenchus avenae |
| U2_SV_163 | Mononchida | Mononchidae | Mononchus | Mononchus truncatus | 0 | 100 | 761/761 | KJ636355 etc | Predator | 4 | Enoplea | Dorylaimia | Mononchida | Ambiguous_taxa |
| U2_SV_179 | Chromadorida | Cyatholaimidae | Achromadora | Achromadora sp.* | 0 | 99.76 | 760/765 | AY284717 etc | Eukaryote feeder | 3 | Chromadorea | NA | Chromadorida | Achromadora sp. JH-2004 |
| U2_SV_180 | Monhysterida | Monhysteridae | Eumonhystera | Eumonhystera cf. hungarica 1 JH-2014* | 1e-172 | 93.54 | 617/750 | KJ636237 | Bacteria feeder | 2 | Chromadorea | NA | Monhysterida | NA |
| U2_SV_197 | Rhabditida | Cephalobidae | Eucephalobus, Cephalobus | Eucephalobus laevis, Cephalobus sp. | 0 | 100 | 673/673 | AY911991 etc | Bacteria feeder/Bacteria feeder | 2/2 | Chromadorea | NA | Rhabditida | NA |
| U2_SV_211 | Plectida | Plectidae | Plectus | Plectus sp.* | 0 | 99.51 | 750/754 | DQ275358 | Bacteria feeder | 2 | Chromadorea | NA | Araeolaimida | Anaplectus grandepapillatus |
| U2_SV_226 | Monhysterida | Monhysteridae | Eumonhystera | Eumonhystera filiformis* | 0 | 99.27 | 747/763 | AY593937 etc | Bacteria feeder | 2 | Chromadorea | NA | Monhysterida | Paralamyctes environmental sample |
| U2_SV_237 | Enoplida | Trischistomatidae | Trischistoma | Trischistoma sp. | 0 | 100 | 761/761 | JN673802 etc | Predator | 3 | Enoplea | Enoplia | Triplonchida | Trischistoma triregium |
| U2_SV_266 | Triplonchida | Odontolaimidae | Odontolaimus | Odontolaimus sp. | 0 | 99.52 | | LC186624 | Bacteria feeder | 3 | Enoplea | Enoplia | Triplonchida | Odontolaimus sp. OdLaSp1 |
| U2_SV_270 | Monhysterida | Monhysteridae | Eumonhystera | Eumonhystera cf. simplex JH-2004* | 0 | 98.8 | 747/756 | AY284692 | Bacteria feeder | 2 | Chromadorea | NA | Monhysterida | NA |
| U2_SV_279 | Dorylaimida | Nordiidae | Longidorella | Longidorella parva* | 0 | 98.8 | 739/741 | AY146525 | Plant feeder | 4 | Enoplea | Dorylaimia | Dorylaimida | Enchodelus veletensis |
| U2_SV_282 | Mononchida | Mylonchulidae | Mylonchulus | Mylonchulus sigmaturus | 0 | 100 | 765/765 | AB361447 etc | Predator | 4 | Enoplea | Dorylaimia | Mononchida | Mylonchulus sp. JH-2004 |
| U2_SV_302 | Mononchida | Mononchidae, Mylonchulidae | Prionchulus, Mylonchulus | Prionchulus oleksandri, Mylonchulus cf. incurvus TSH-2005 | 0 | 100 | 765/765 | MG969498 etc | Predator/Predator | 4/4 | Enoplea | Dorylaimia | Mononchida | Mylonchulus hawaiiensis |

| | | | | | | | | | | | | | | |
|---|---|---|---|---|---|---|---|---|---|---|---|---|---|---|
| U2_SV_303 | Rhabditida, (Triplonchida) | Cephalobidae, (Diphtherophoridae) | Acrobeloides, Cephalobus, (Tylolaimophorus) | Acrobeloides sp., Cephalobus sp., (Tylolaimophorus sp.) | 0 | 99.76 | 758/758 | MK636581 etc | Bacteria feeder/Bacteria feeder | 2/2 | Chromadorea | NA | Rhabditida | NA |
| U2_SV_317 | Dorylaimida | Dorylaimidae | Mesodorylaimus | Mesodorylaimus cf. recurvus Konza IAD-37 | 0 | 100 | 765/765 | AY146489 | Omnivore | 4 | Enoplea | Dorylaimia | Dorylaimida | NA |
| U2_SV_321 | Triplonchida | Trichodoridae | Paratrichodorus | Paratrichodorus minor | 0 | 100 | 763/763 | MN911168 etc | Plant feeder | 4 | Enoplea | Enoplia | Triplonchida | Paratrichodorus porosus |
| U2_SV_327 | Triplonchida | Prismatolaimidae | Prismatolaimus | Prismatolaimus sp.* | 0 | 98.31 | 725/760 | LC186858 etc | Bacteria feeder | 3 | Enoplea | Enoplia | Triplonchida | Ambiguous_taxa |
| U2_SV_341 | Triplonchida | Prismatolaimidae | Prismatolaimus | Prismatolaimus sp.* | 0 | 100 | 756/763 | AB619105 etc | Bacteria feeder | 3 | Enoplea | Enoplia | Triplonchida | Ambiguous_taxa |
| U2_SV_357 | Rhabditida | Rhabditidae | Distolabrellus | Distolabrellus veechi | 0 | 100 | 756/756 | AF082999 | Bacteria feeder | 1 | Chromadorea | NA | Rhabditida | NA |
| U2_SV_359 | Monhysterida | Monhysteridae | Eumonhystera | Eumonhystera sp. 1 JH-2014* | 0 | 99.52 | 756/767 | KJ636251 | Bacteria feeder | 2 | Chromadorea | NA | Monhysterida | NA |
| U2_SV_360 | Monhysterida | Monhysteridae | Eumonhystera | Eumonhystera filiformis* | 0 | 99.52 | 752/763 | AY593937 etc | Bacteria feeder | 2 | Chromadorea | NA | Monhysterida | Paralamyctes environmental sample |
| U2_SV_364 | Dorylaimida | Aporcelaimidae | Akrotonus | Akrotonus vigor* | 0 | 97.11 | 699/710 | AY146476 | *Omnivore* [f] | *5* [f] | Enoplea | Dorylaimia | Dorylaimida | NA |
| U2_SV_411 | Rhabditida | Ecphyadophoridae | Lelenchus | Lelenchus sp. MB-2019* | 1e-158 | 91.95 | 571/693 | MN542204 | Plant feeder | 2 | Chromadorea | NA | Rhabditida | NA |
| U2_SV_413 | Rhabditida | Tylenchidae | Basiria | Basiria sp. | 0 | 100 | 761/761 | MK639391 etc | Plant feeder | 2 | Chromadorea | NA | Rhabditida | NA |
| U2_SV_423 | Plectida | Plectidae | Plectus | Plectus sp. | 0 | 100 | 763/763 | LC382088 etc | Bacteria feeder | 2 | Chromadorea | NA | Araeolaimida | NA |
| U2_SV_436 | Dorylaimida | Qudsianematidae | Discolaimus | Discolaimus texanus | 0 | 100 | 767/767 | AY146485 | Omnivore | 4 | Enoplea | Dorylaimia | Dorylaimida | NA |
| U2_SV_444 | Rhabditida | Ecphyadophoridae | Lelenchus | Lelenchus sp. MB-2019* | 5e-157 | 91.71 | 566/699 | MN542204 | Plant feeder | 2 | Chromadorea | NA | Rhabditida | NA |
| U2_SV_470 | Triplonchida | Prismatolaimidae | Prismatolaimus | Prismatolaimus sp. | 0 | 100 | 763/763 | LC186686 etc | Bacteria feeder | 3 | Enoplea | Enoplia | Triplonchida | Ambiguous_taxa |
| U2_SV_479 | Dorylaimida | Qudsianematidae | Microdorylaimus, Eudorylaimus | Microdorylaimus angleus*, cf. Eudorylaimus carteri MtR GWN-02* | 0 | 98.55 | 732/739 | AY146526 etc | Omnivore/Omnivore | 4/4 | Enoplea | Dorylaimia | Dorylaimida | Enchodelus veletensis |
| U2_SV_489 | Araeolaimida | Comesomatidae | Sabatieria | Sabatieria sp.* | 1e-172 | 93.56 | 617/706 | MN250140 etc | NA | (−) | Chromadorea | NA | Monhysterida | NA |
| U2_SV_494 | Monhysterida | Monhysteridae | Eumonhystera | Eumonhystera cf. simplex JH-2004* | 0 | 98.8 | 747/756 | AY284692 etc | Bacteria feeder | 2 | Chromadorea | NA | Monhysterida | NA |
| U2_SV_506 | Dorylaimida | Belondiridae | Axonchoides, Oxydirus | Axonchoides smokyensis*, Oxydirus nethus* | 0 | 97.34 | 701/739 | JX885740 etc | Plant feeder/Predator | 5/5 | Enoplea | Dorylaimia | Dorylaimida | NA |
| U2_SV_539 | Dorylaimida | Qudsianematidae | Microdorylaimus | Microdorylaimus angleus* | 0 | 100 | 743/765 | AY146526 | Omnivore | 4 | Enoplea | Dorylaimia | Dorylaimida | NA |
| U2_SV_556 | Chromadorida | Cyatholaimidae | Achromadora | Achromadora ruricola | 0 | 98.55 | 732/732 | AY593941 | Eukaryote feeder | 3 | Chromadorea | NA | Chromadorida | Achromadora ruricola |
| U2_SV_581 | Monhysterida | Monhysteridae | Eumonhystera | Eumonhystera sp. | 0 | 99.04 | 745/745 | KJ636251 etc | Bacteria feeder | 2 | Chromadorea | NA | Monhysterida | NA |
| U2_SV_586 | Dorylaimida | Dorylaimidae | Laimydorus | Laimydorus sp. TX1-07* | 0 | 99.03 | 741/756 | AY146502 | Omnivore | 4 | Enoplea | Dorylaimia | Dorylaimida | NA |
| U2_SV_592 | Chromadorida | Cyatholaimidae | Achromadora | Achromadora ruricola | 0 | 99.28 | 749/749 | AY593941 | Eukaryote feeder | 3 | Chromadorea | NA | Chromadorida | Achromadora ruricola |
| U2_SV_630 | Chromadorida | Cyatholaimidae | Achromadora | Achromadora sp. | 1e-66 | 100 | 415/415 | LC186660 etc | Eukaryote feeder | 3 | Chromadorea | NA | Chromadorida | NA |
| U2_SV_631 | Monhysterida | Monhysteridae | Eumonhystera | Eumonhystera cf. vulgaris 1 JH-2014* | 0 | 99.04 | 745/756 | KJ636250 | Bacteria feeder | 2 | Chromadorea | NA | Monhysterida | NA |
| U2_SV_641 | Chromadorida | Cyatholaimidae | Achromadora | Achromadora ruricola | 0 | 97.83 | 715/715 | AY593941 | Eukaryote feeder | 3 | Chromadorea | NA | Chromadorida | NA |
| U2_SV_651 | Triplonchida | Prismatolaimidae | Prismatolaimus | Prismatolaimus sp.* | 0 | 98.08 | 721/739 | LC186686 etc | Bacteria feeder | 3 | Enoplea | Enoplia | Triplonchida | Ambiguous_taxa |
| U2_SV_657 | Chromadorida | Cyatholaimidae | Achromadora | Achromadora ruricola | 0 | 98.79 | 737/737 | AY593941 | Eukaryote feeder | 3 | Chromadorea | NA | Chromadorida | Achromadora ruricola |
| U2_SV_659 | Monhysterida | Monhysteridae | Eumonhystera | Eumonhystera filiformis* | 0 | 97.34 | 702/713 | AY593937 etc | Bacteria feeder | 2 | Chromadorea | NA | Monhysterida | NA |
| U2_SV_667 | Rhabditida | Cephalobidae | Pseudacrobeles | Pseudacrobeles variabilis | 0 | 99.27 | 747/747 | AF202150 | Bacteria feeder | 2 | Chromadorea | NA | Rhabditida | NA |
| U2_SV_679 | Enoplida | Alaimidae | Alaimus | Alaimus sp. | 0 | 98.55 | 730/730 | LC186877 etc | Bacteria feeder | 4 | Enoplea | Enoplia | Enoplida | NA |
| U2_SV_682 | Monhysterida | Monhysteridae | Eumonhystera | Eumonhystera cf. vulgaris 1 JH-2014* | 0 | 98.80 | 739/750 | KJ636250 | Bacteria feeder | 2 | Chromadorea | NA | Monhysterida | NA |
| U2_SV_730 | Monhysterida | Monhysteridae | Eumonhystera | Eumonhystera cf. hungarica 1 JH-2014 | 0 | 95.98 | 678/678 | KJ636237 | Bacteria feeder | 2 | Chromadorea | NA | Monhysterida | NA |
| U2_SV_758 | Rhabditida | Rhabditidae | Mesorhabditis | Mesorhabditis sp. 339Meso2 | 0 | 100 | 756/756 | DQ275369 | Bacteria feeder | 1 | Chromadorea | NA | Rhabditida | NA |
| U2_SV_767 | Triplonchida | Prismatolaimidae | Prismatolaimus | Prismatolaimus sp. | 0 | 99.76 | 758/758 | LC186851 etc | Bacteria feeder | 3 | Enoplea | Enoplia | Triplonchida | Ambiguous_taxa |
| U2_SV_791 | Rhabditida | Cephalobidae | Cephalobus | Cephalobus cubaensis | 0 | 98.32 | 736/736 | AF202161 | Bacteria feeder | 2 | Chromadorea | NA | Rhabditida | Cephalobus cubaensis |
| U2_SV_822 | Monhysterida | Monhysteridae | Eumonhystera | Eumonhystera sp.* | 0 | 99.04 | 745/750 | KJ636251 etc | Bacteria feeder | 2 | Chromadorea | NA | Monhysterida | NA |
| U2_SV_843 | Monhysterida | Monhysteridae | Eumonhystera | Eumonhystera cf. vulgaris 1 JH-2014* | 6e-171 | 93.30 | 612/739 | KJ636237 etc | Bacteria feeder | 2 | Chromadorea | NA | Monhysterida | NA |
| U2_SV_873 | Mononchida | Mylonchulidae | Mylonchulus | Mylonchulus sp. | 0 | 100 | 765/765 | LC186773 etc | Predator | 4 | Enoplea | Dorylaimia | Mononchida | Ambiguous_taxa |
| U2_SV_896 | Chromadorida | Cyatholaimidae | Achromadora | Achromadora ruricola | 0 | 98.07 | 721/721 | AY593941 | Eukaryote feeder | 3 | Chromadorea | NA | Chromadorida | NA |

| | | | | | | | | | | | | | | |
|---|---|---|---|---|---|---|---|---|---|---|---|---|---|---|
| U2_SV_901 | Rhabditida | Tylenchidae | Basiria | Basiria sp. 2 JH-2014 | 0 | 99.76 | 756/756 | KJ869354 | Plant feeder | 2 | Chromadorea | NA | Rhabditida | Basiria sp. 2 JH-2014 |
| U2_SV_915 | Dorylaimida | Qudsianematidae | Microdorylaimus | Microdorylaimus angleus | 0 | 99.52 | 754/854 | AY146526 | Omnivore | 4 | Enoplea | Dorylaimia | Dorylaimida | NA |
| U2_SV_931 | Rhabditida | Rhabditidae | Mesorhabditis | Mesorhabditis sp. | 0 | 100 | 756/756 | U73452 | Bacteria feeder | 1 | Chromadorea | NA | Rhabditida | NA |
| U2_SV_935 | Enoplida | Alaimidae | Alaimus | Alaimus cf. acutus Konza VAA-142 | 0 | 100 | 763/763 | AY146468 | Bacteria feeder | 4 | Enoplea | Enoplia | Enoplida | Alaimus sp. PDL-2005 |
| U2_SV_960 | Rhabditida | Hoplolaimidae | Helicotylenchus | Helicotylenchus sp. | 0 | 100 | 765/765 | KM014493 etc | Plant feeder | 3 | Chromadorea | NA | Rhabditida | NA |
| U2_SV_1114 | Rhabditida | Rhabditidae | Mesorhabditis | Mesorhabditis sp. | 0 | 100 | 750/750 | DQ080581 | Bacteria feeder | 1 | Chromadorea | NA | Rhabditida | NA |
| U2_SV_1142 | Monhysterida | Monhysteridae | Eumonhystera | Eumonhystera filiformis* | 0 | 96.62 | 688/743 | KJ636219 | Bacteria feeder | 2 | Chromadorea | NA | Monhysterida | NA |
| U2_SV_1161 | Triplonchida | Diphtherophoridae | Diphtherophora | Diphtherophora sp.* | 2e-180 | 94.69 | 643/686 | KY115102 etc | Fungus feeder | 3 | Enoplea | Enoplia | Triplonchida | NA |
| U2_SV_1168 | Monhysterida | Monhysteridae | Eumonhystera | Eumonhystera filiformis* | 0 | 98.79 | 736/752 | AY593937 etc | Bacteria feeder | 2 | Chromadorea | NA | Monhysterida | Paralamyctes environmental sample |
| U2_SV_1180 | Rhabditida | Tylenchidae | Labrys | Labrys khuzestanensis | 0 | 98.35 | 745/745 | MK818501 | Plant feeder | 2 | Chromadorea | NA | Rhabditida | NA |
| U2_SV_1233 | Dorylaimida | Aporcelaimidae | Aporcelaimellus | Aporcelaimellus sp. | 0 | 100 | 763/763 | MG993563 etc | Omnivore | 5 | Enoplea | Dorylaimia | Dorylaimida | Ambiguous_taxa |
| U2_SV_1313 | Mermithida | Mermithidae | Strelkovimermis | Strelkovimermis spiculatus* | 1e-168 | 93.24 | 604/647 | KP270704 etc | NA | (−) | Enoplea | Dorylaimia | Mermithida | NA |
| U2_SV_1366 | Monhysterida | Monhysteridae | Eumonhystera | Eumonhystera cf. vulgaris 1 JH-2014* | 0 | 98.80 | 739/745 | KJ636250 | Bacteria feeder | 2 | Chromadorea | NA | Monhysterida | NA |
| U2_SV_1461 | Monhysterida | Monhysteridae | Geomonhystera | Geomonhystera sp.* | 0 | 99.03 | 743/752 | GQ503077 etc | Bacteria feeder | 2 | Chromadorea | NA | Monhysterida | NA |
| U2_SV_1483 | Rhabditida | Ecphyadophoridae | Ecphyadophora | Ecphyadophora quadralata* | 0 | 96.21 | 684/701 | EU707187 | Plant feeder | 2 | Chromadorea | NA | Rhabditida | NA |
| U2_SV_1503 | Rhabditida | Tylenchidae | Discopersicus | Discopersicus iranicus | 0 | 100 | 761/761 | KM502981 | *Plant feeder* | *2* | Chromadorea | NA | Rhabditida | Basiria duplexa |
| U2_SV_1504 | Chromadorida | Cyatholaimidae | Achromadora | Achromadora ruricola | 0 | 98.55 | 732/732 | AY593941 | Eukaryote feeder | 3 | Chromadorea | NA | Chromadorida | Achromadora ruricola |
| U2_SV_1538 | Rhabditida | Tylenchidae | Labrys | Labrys sp. 1 YP-2018 | 0 | 97.40 | 719/719 | MG686086 | Plant feeder | 2 | Chromadorea | NA | Rhabditida | NA |
| U2_SV_1544 | Rhabditida | Pratylenchidae | Pratylenchus | Pratylenchus sp. | 0 | 100 | 761/761 | MN756509 etc | Plant feeder | 2 | Chromadorea | NA | Rhabditida | Pratylenchus penetrans |
| U2_SV_1589 | Enoplida | Rhabdolaimidae | Rhabdolaimus | Rhabdolaimus aquaticus | 0 | 100 | 761/761 | FJ969139 | Bacteria feeder | 3 | Enoplea | Enoplia | Enoplida | Rhabdolaimus cf. terrestris JH-2004 |
| U2_SV_1598 | Monhysterida | Monhysteridae | Eumonhystera | Eumonhystera filiformis* | 0 | 98.06 | 719/730 | AY593937 etc | Bacteria feeder | 2 | Chromadorea | NA | Monhysterida | NA |
| U2_SV_1677 | Enoplida | Ironidae | Ironus | Ironus sp. | 0 | 100 | 763/763 | AJ966487 etc | Predator | 4 | Enoplea | Enoplia | Enoplida | NA |
| U2_SV_1874 | Rhabditida | Anguinidae | Ditylenchus | Ditylenchus dipsaci | 0 | 100 | 761/761 | KJ636296 | Fungus feeder | 2 | Chromadorea | NA | Rhabditida | Ditylenchus dipsaci |
| U2_SV_1942 | Rhabditida | Anguinidae | Ditylenchus | Ditylenchus sp. 1 JH-2003 | 0 | 100 | 763/763 | AY284637 | Fungus feeder | 2 | Chromadorea | NA | Rhabditida | NA |
| U2_SV_2030 | Rhabditida | Ecphyadophoridae | Lelenchus | Lelenchus sp. MB-2019* | 2e-156 | 91.71 | 564/728 | MN542204 | Plant feeder | 2 | Chromadorea | NA | Rhabditida | NA |
| U2_SV_2035 | Enoplida | Alaimidae | Alaimus | Alaimus parvus | 0 | 97.09 | 695/695 | AY284738 | Bacteria feeder | 4 | Enoplea | Enoplia | Enoplida | Alaimus parvus |
| U2_SV_2040 | Chromadorida | Cyatholaimidae | Achromadora | Achromadora sp.* | 4e-168 | 99.40 | 769/775 | LC186707 etc | Eukaryote feeder | 3 | Chromadorea | NA | Chromadorida | Prodesmodora circulata |
| U2_SV_2153 | Plectida | Plectidae | Plectus | Plectus sp. 295Ple3 | 7e-145 | 100 | 700/700 | DQ275358 | Bacteria feeder | 2 | Chromadorea | NA | Araeolaimida | Anaplectus grandepapillatus |
| U2_SV_2222 | Rhabditida | Tylenchidae | Miculenchus | Miculenchus brevisalvus | 3e-119 | 85.61 | 440/440 | MF599081 | Plant feeder | 2 | Chromadorea | NA | Rhabditida | NA |
| U2_SV_2484 | Monhysterida | Monhysteridae | Geomonhystera | Geomonhystera cf. aenariensis Konza IVDD-147 | 0 | 99.52 | 754/754 | DQ080576 | Bacteria feeder | 2 | Chromadorea | NA | Monhysterida | Geomonhystera villosa |
| U2_SV_2566 | Rhabditida | Hoplolaimidae | Helicotylenchus | Helicotylenchus sp. | 4e-137 | 100 | 595/595 | KM014493 | Plant feeder | 3 | Chromadorea | NA | Rhabditida | NA |
| U2_SV_2761 | Rhabditida | Neodiplogasteridae | Pristionchus | Pristionchus japonicus | 0 | 100 | 767/767 | KT188850 | Predator | 1 | Chromadorea | NA | Diplogasterida | Pristionchus quartusdecimus |

[a]Nematode-derived SVs from region U2 were identified from eight data sets (i.e., amplicons generated from the soil DNAs from the agricultural field).

[b]The top hit of species, genus, family, order, e-value, percent identity, and total score (top hit score/highest score) from BLASTN search are indicated. The top hit sequences without taxonomic data, such as environmental samples, were omitted. The second-closest species to the query SV sequences are shown with asterisks to use the taxonomic assignment of the SVs.

[c] The feeding types and the cp groups of the SVs were predicted based on those of the closest genus based on the functional guildes at the Nemaplex home page (http://nemaplex.ucdavis.edu/Uppermnus/topmnu.htm). The feeding types and the cp groups of the SVs with multiple closest genus separately indicate the corresponding types and values by slash. The parasist was predicted by previous publications, as described in the Materials and methods section. (−): unknown cp group. NA: not assigned to the defined feeding type.

[d]The taxonomic ranks of D7–D10 corresponding to the SVs are indicated. NA: not assigned.

[e]Sole hit species derived from a different phylum shared the same e-value with multiple hit species and is enclosed in parentheses.

[f]The feeding type and the cp value of the SV in italic were predicted by the closest family based on the functional guildes at the Nemaplex home page.
