# Supplementary figures and images for: Use of universal primers for the 18S ribosomal RNA gene and whole soil DNAs to reveal the taxonomic structures of soil nematodes by high-throughput amplicon sequencing
Source: PLoS One. 2021 Nov 15;16(11):e0259842. doi: 10.1371/journal.pone.0259842 (PMC8592498; doi:10.1371/journal.pone.0259842)

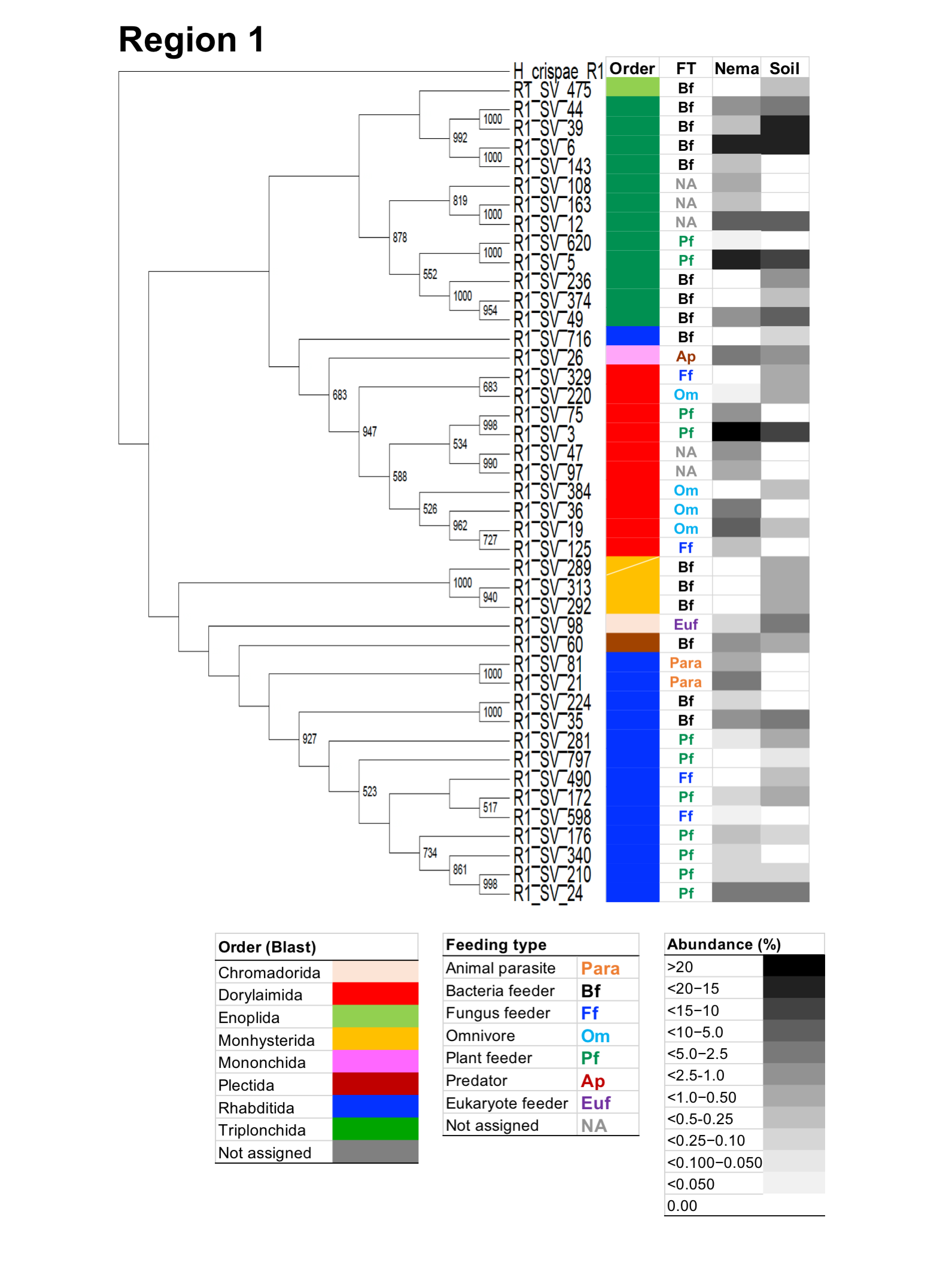

Supplement: S1 Fig — A cladogram was prepared using nematode-derived SVs from region 1 and the corresponding SSU gene sequence of H. crispae as the outgroup as described in the Materials and methods section. Bootstrap numbers of over 500 per 1000 are indicated at the nodes of the cladogram. The name of the nematode-derived SV in region 1 was indicated as R1_SV_number at the branch end of the cladogram. Orders and feeding types of the SVs are indicated in the corresponding columns at the right of the cladogram by colored boxes and abbreviations, as shown in the legend boxes. Relative read abundances of nematode-derived SVs in the nematode genomic DNA (Nema) and soil DNA (Soil) from the copse soils are indicated by density boxes. A colored box for order with a slashed line (R1_SV_289) indicates probable nematode order due to cross-hits to the species in other phyla by BLASTN search (see S3 Table legend). (TIFF) [file pone.0259842.s011.tiff]

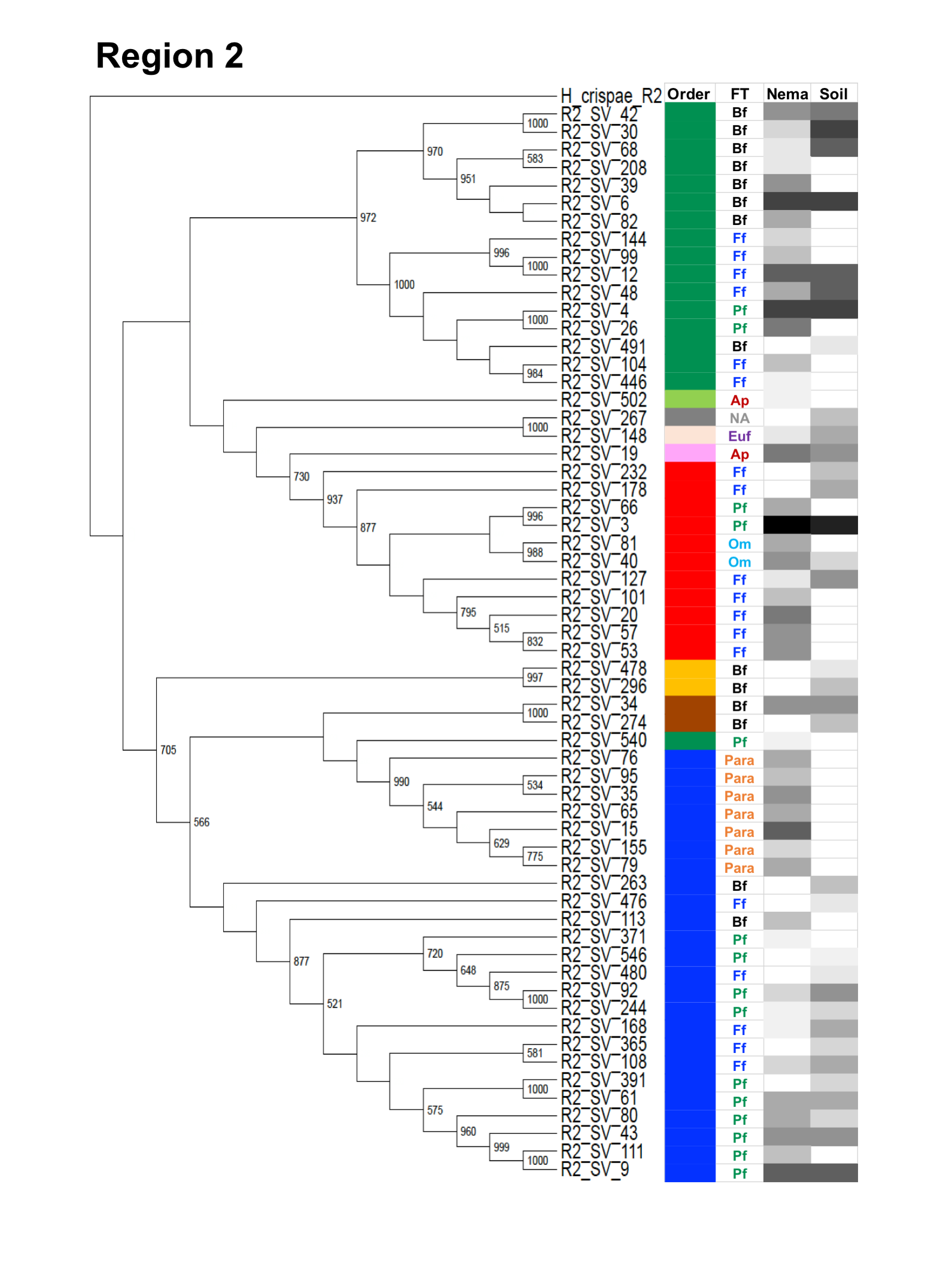

Supplement: S2 Fig — A cladogram was prepared using nematode-derived SVs of region 2 as described in the Materials and methods section. Orders, feeding types, and relative read abundances of the nematode-derived SVs are indicated in the corresponding columns at the right of the cladogram by colored boxes, colored abbreviations, and density boxes, respectively, as detailed in the caption of S1 Fig. (TIFF) [file pone.0259842.s012.tiff]

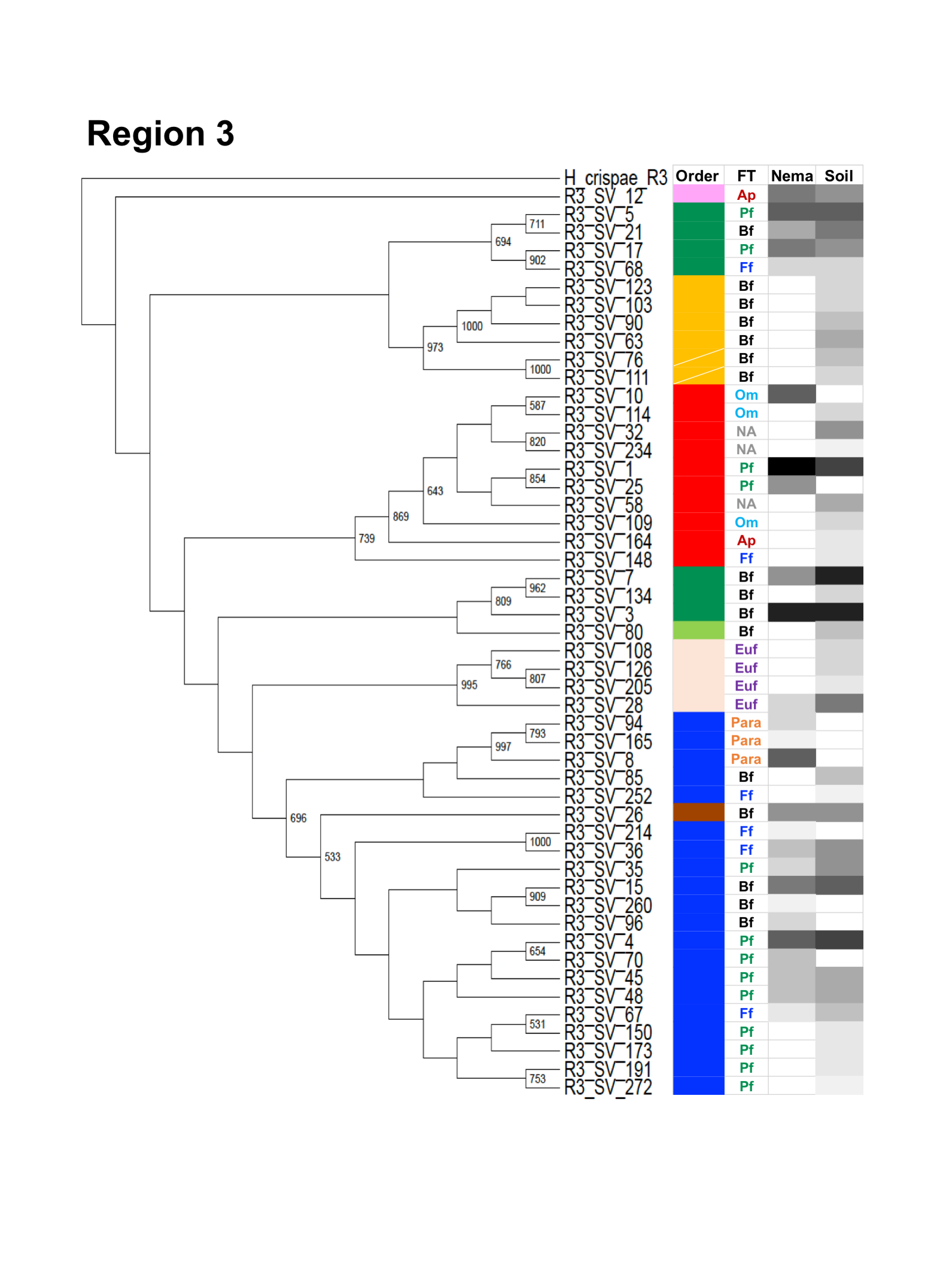

Supplement: S3 Fig — A cladogram was prepared using nematode-derived SVs of region 3 as described in the Materials and methods section. Orders, feeding types, and relative read abundances of the nematode-derived SVs are indicated in the corresponding columns at the right of the cladogram by colored boxes, colored abbreviations, and density boxes, respectively, as detailed in the caption of S1 Fig. (TIFF) [file pone.0259842.s013.tiff]

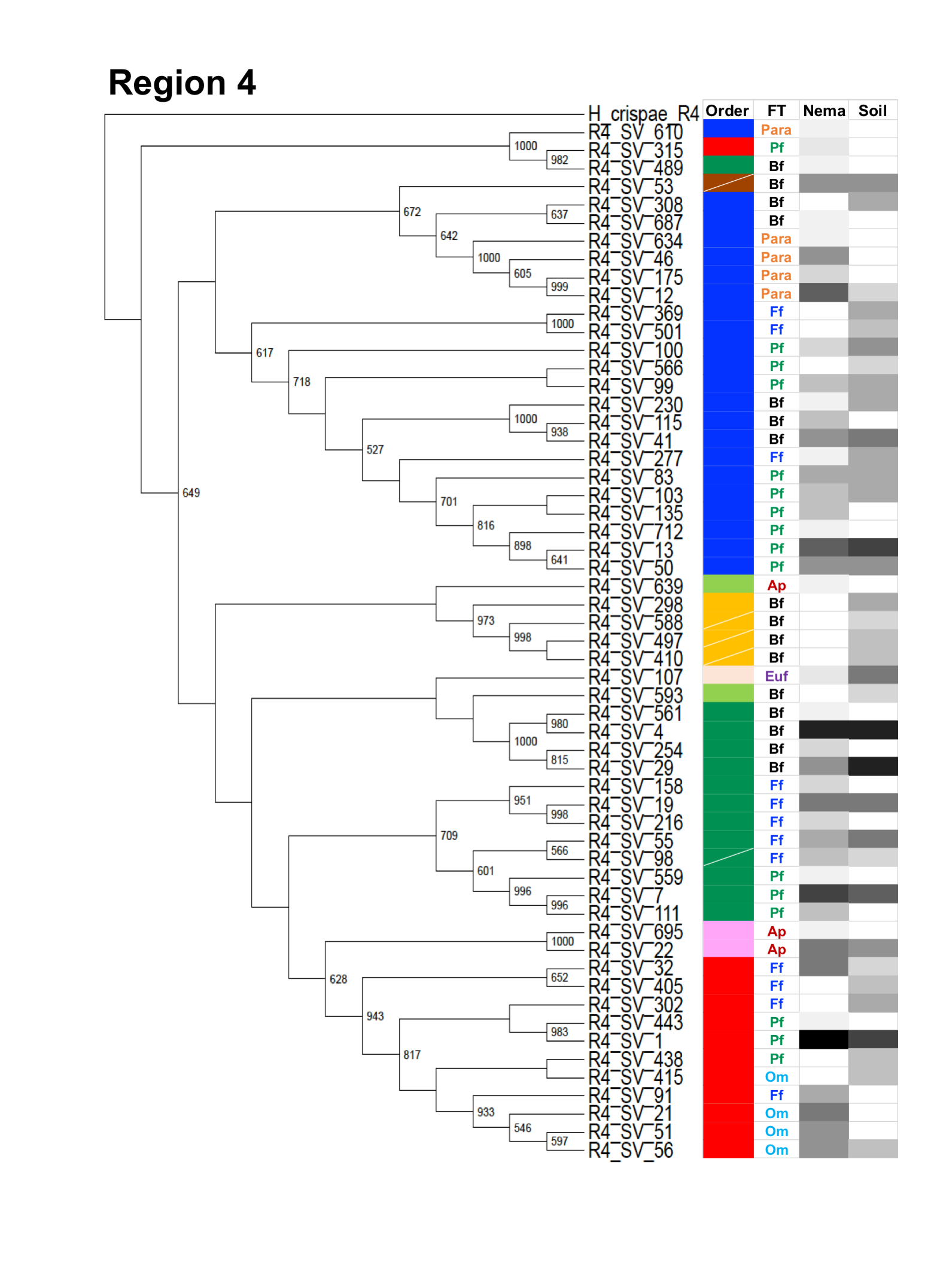

Supplement: S4 Fig — A cladogram was prepared using nematode-derived SVs of region 4 as described in the Materials and methods section. Orders, feeding types, and relative read abundances of the nematode-derived SVs are indicated in the corresponding columns at the right of the cladogram by colored boxes, colored abbreviations, and density boxes, respectively, as detailed in the caption of S1 Fig. (TIFF) [file pone.0259842.s014.tiff]

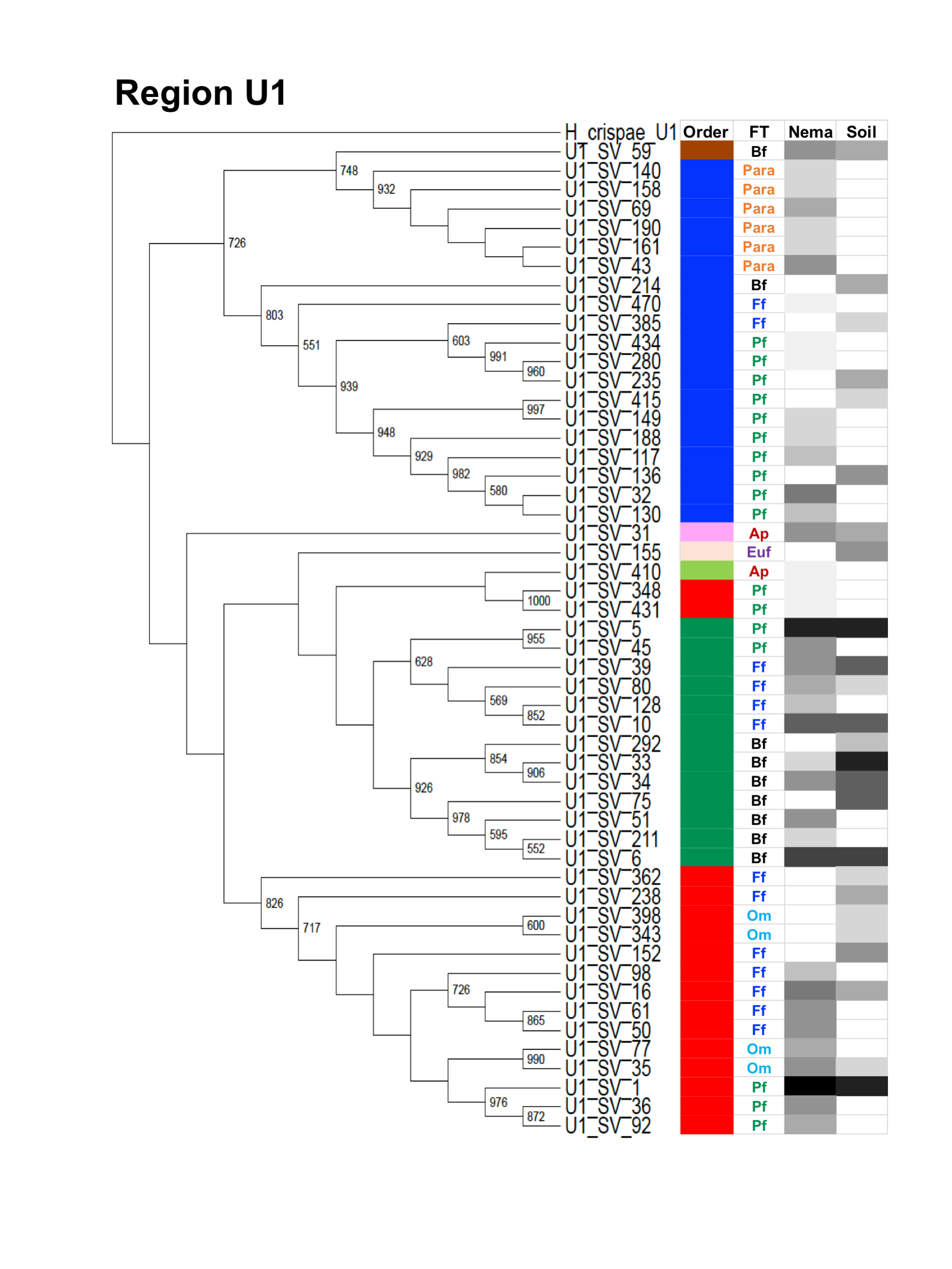

Supplement: S5 Fig — A cladogram was prepared using nematode-derived SVs of region U1 as described in the Materials and methods section. Orders, feeding types, and relative read abundances of the nematode-derived SVs are indicated in the corresponding columns at the right of the cladogram by colored boxes, colored abbreviations, and density boxes, respectively, as detailed in the caption of S1 Fig. (TIFF) [file pone.0259842.s015.tiff]

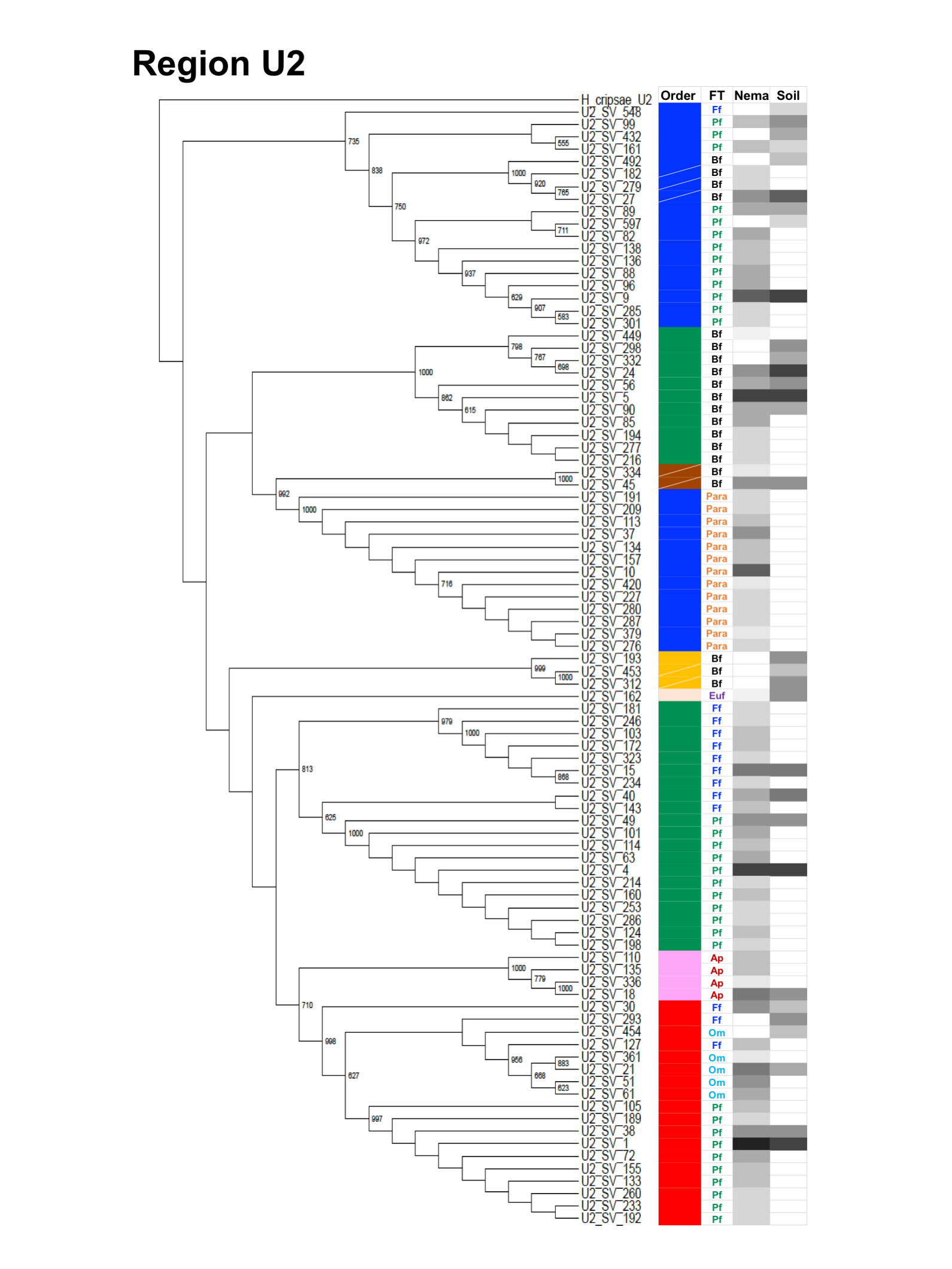

Supplement: S6 Fig — A cladogram was prepared using nematode-derived SVs of region U2 as described in the Materials and methods section. Orders, feeding types, and relative read abundances of the nematode-derived SVs are indicated in the corresponding columns at the right of the cladogram by colored boxes, colored abbreviations, and density boxes, respectively, as detailed in the caption of S1 Fig. (TIFF) [file pone.0259842.s016.tiff]

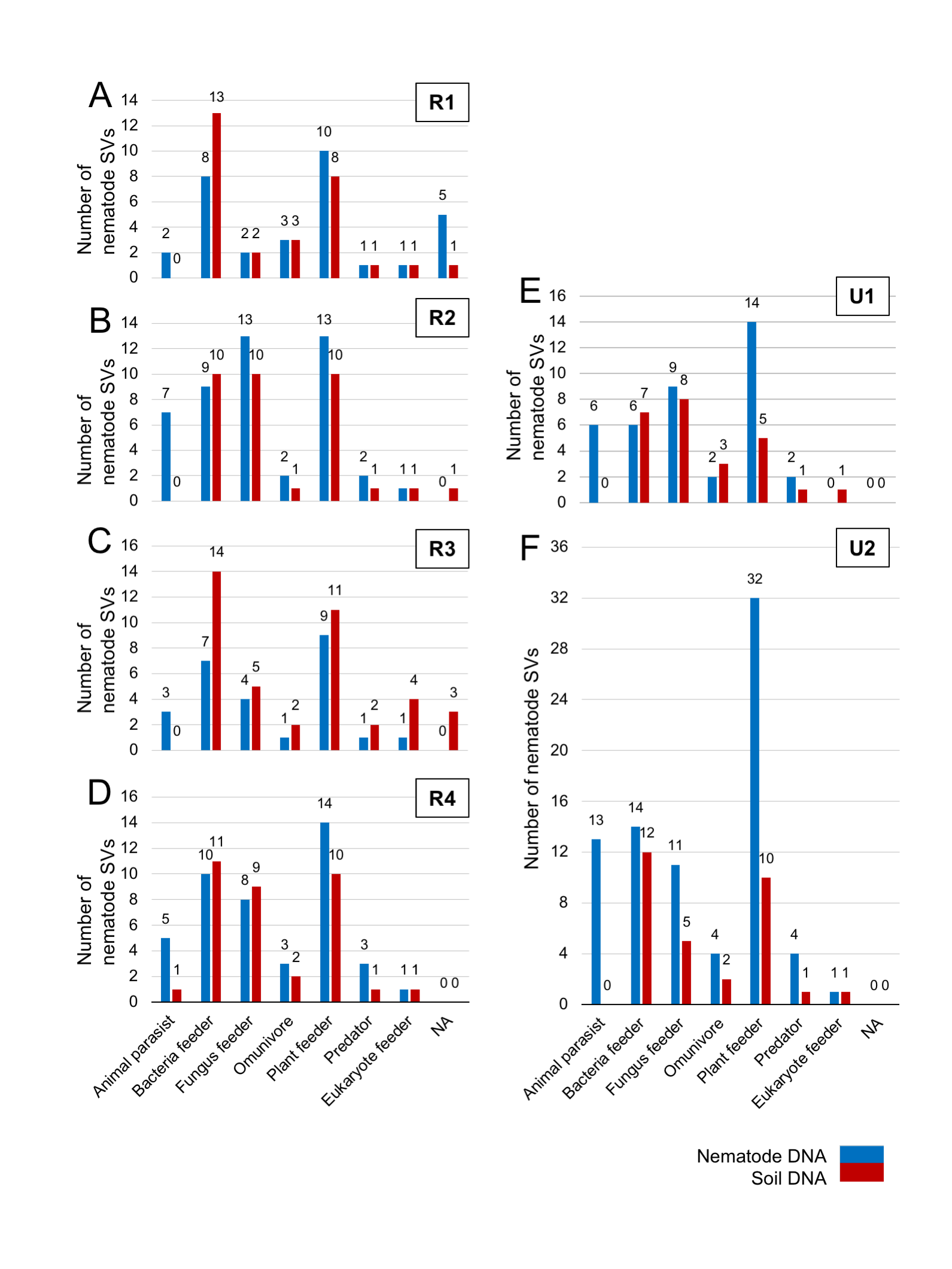

Supplement: S7 Fig — Regional nematode SVs derived from the copse-derived nematode genomic DNA (blue bars) and soil DNA (red bars) were assigned to one of seven feeding types (bacteria feeder, fungus feeder, plant feeder, omnivore, predator, eukaryote feeder, and animal parasite) as described in the Materials and methods section. Numbers of regional nematode SVs in feeding type are shown by colored bars in regions 1 (A), 2 (B), 3 (C), 4 (D), U1 (E), and U2 (F), respectively. SVs with multiple feeding types are classified as “not assigned (NA).” Target SSU regions are indicated at the top right side of the corresponding bar charts. (TIFF) [file pone.0259842.s017.tiff]

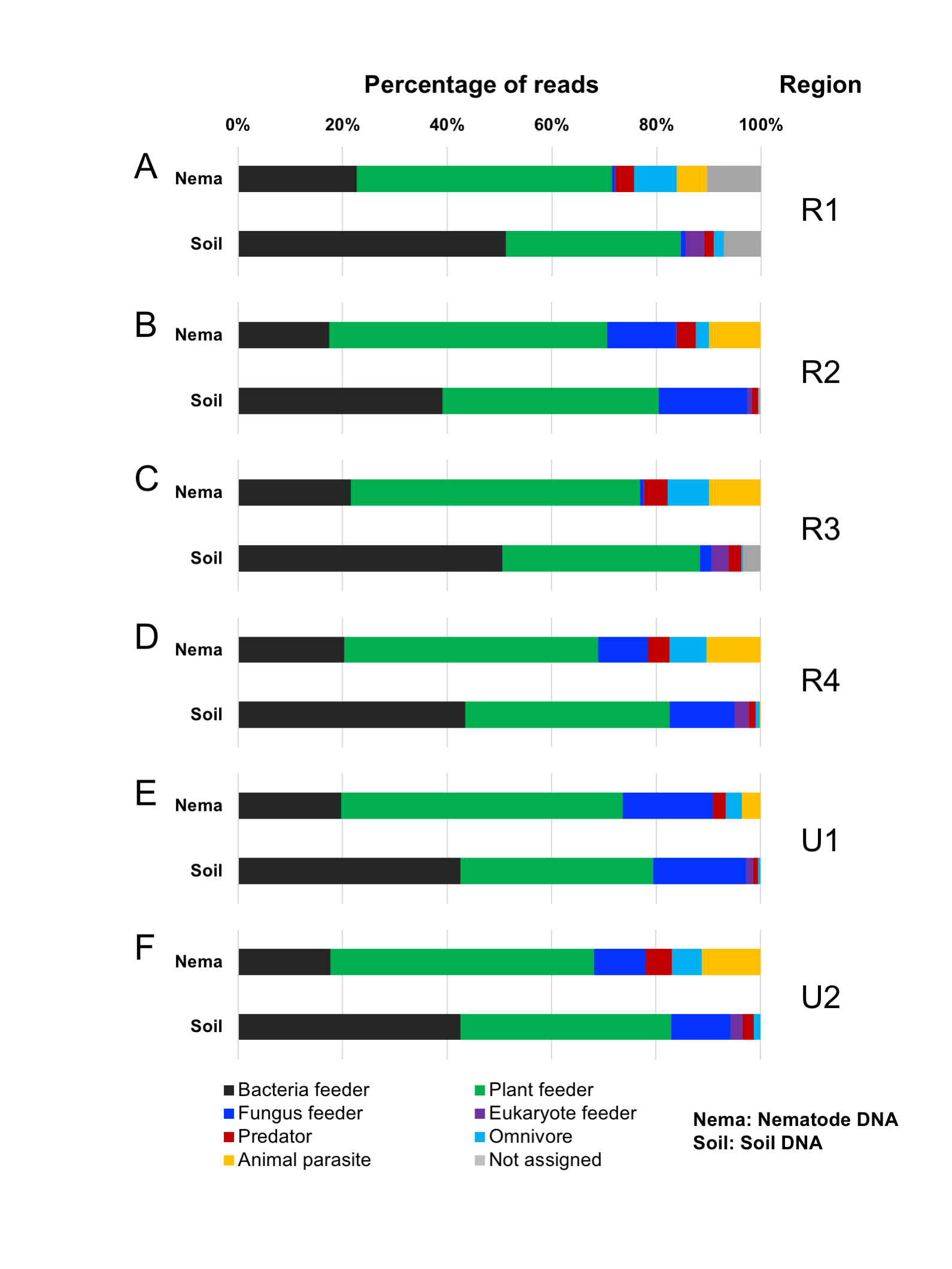

Supplement: S8 Fig — Feeding types of the regional nematode SVs were assigned as described in the legend for S7 Fig. The percentages of the sequence reads of nematode SVs identified from the copse-derived nematode DNA (Nema) and soil DNA (Soil) in feeding type are shown by colored fractions of horizontal bars in regions 1 (A), 2 (B), 3 (C), 4 (D), U1 (E), and U2 (F), respectively. Each feeding type is indicated by color as shown at the bottom of (F). The fractions of SVs with multiple feeding types are classified as “not assigned” and indicated in light gray. Target SSU regions are indicated at the right side of the corresponding horizontal bar charts. (TIFF) [file pone.0259842.s018.tiff]

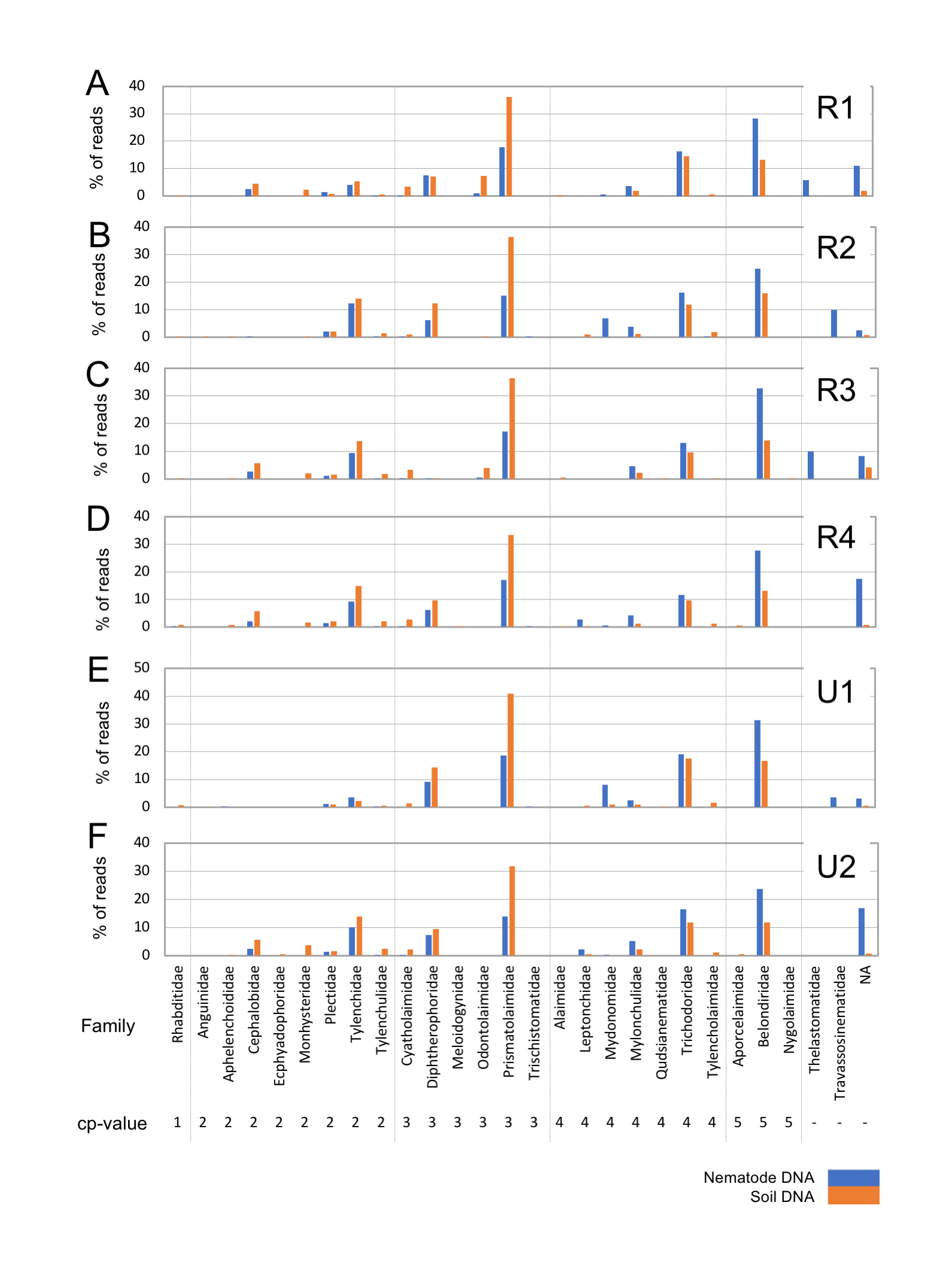

Supplement: S9 Fig — Percentages of sequence reads of regional nematode SVs identified from the copse-derived nematode genomic DNA (blue bars) and soil DNA (orange bars) are indicated in each family by histograms for regions 1–4, U1 and U2 (A–F), respectively. The cp-values, shown at the bottom of figure, indicate the nematode’s life strategy characteristics as described in the Materials and methods section. Nematode families are aligned by their cp values (1–5); undefined cp values are indicated by a hyphen (-). NA: not assigned to a single family. Target SSU regions are indicated at the top right side of the corresponding histograms. (TIFF) [file pone.0259842.s019.tiff]

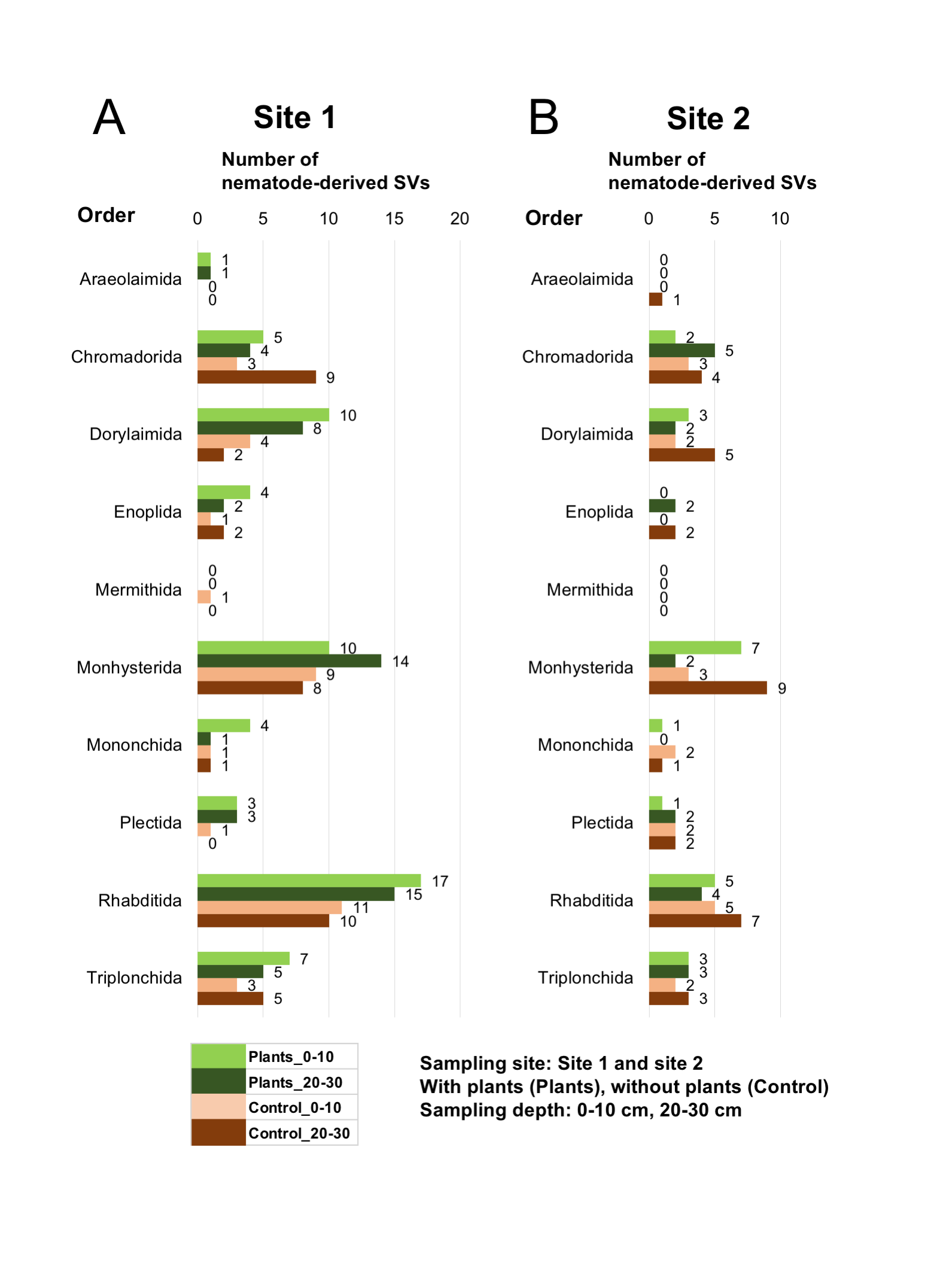

Supplement: S10 Fig — Nematode-derived SVs were obtained from four soil samples isolated from the surface (0–10) and deep (20–30) layers at the sampling points without (control) and with (plants) growing sweet potato at sites 1 (A) and 2 (B), respectively. The number of nematode-derived SVs in each sample is indicated by colored horizontal bars; the samples and colors are indicated in the legend box. (TIFF) [file pone.0259842.s020.tiff]

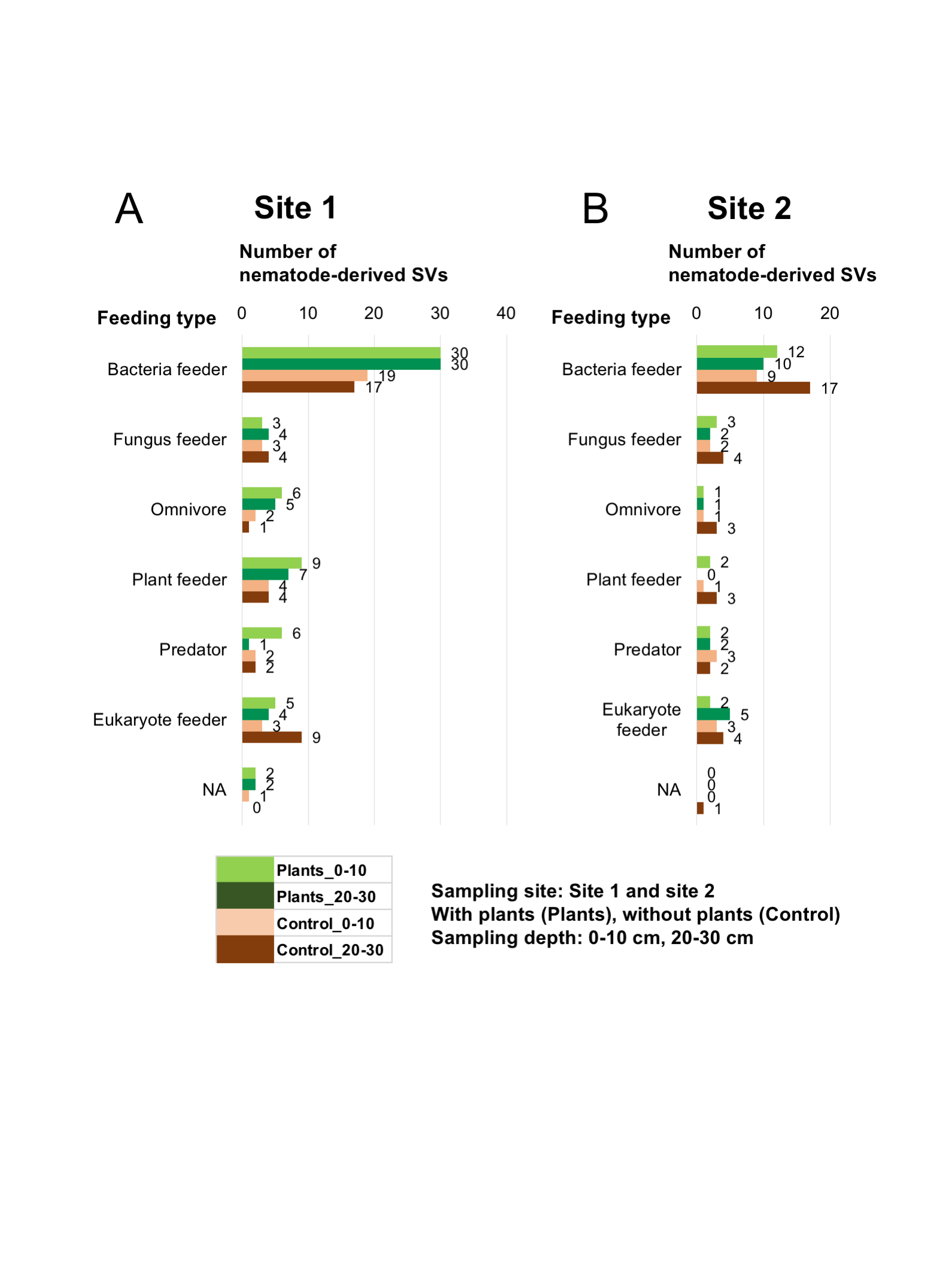

Supplement: S11 Fig — Nematode-derived SVs identified from four soil samples at sites 1 (A) and 2 (B) of the agricultural field were assigned to one of six feeding types as described in the Materials and methods section. Four soil samples were isolated from the surface (0–10) and deep (20–30) layers at the sampling point with (plants) and without (control) growing sweet potato at each site, as shown by colors in the legend box at the bottom of the figure. Number of nematode-derived SVs in feeding type is shown in each sample by the corresponding color bar. The SVs with multiple feeding types are classified as “not assigned (NA)”. (TIFF) [file pone.0259842.s021.tiff]

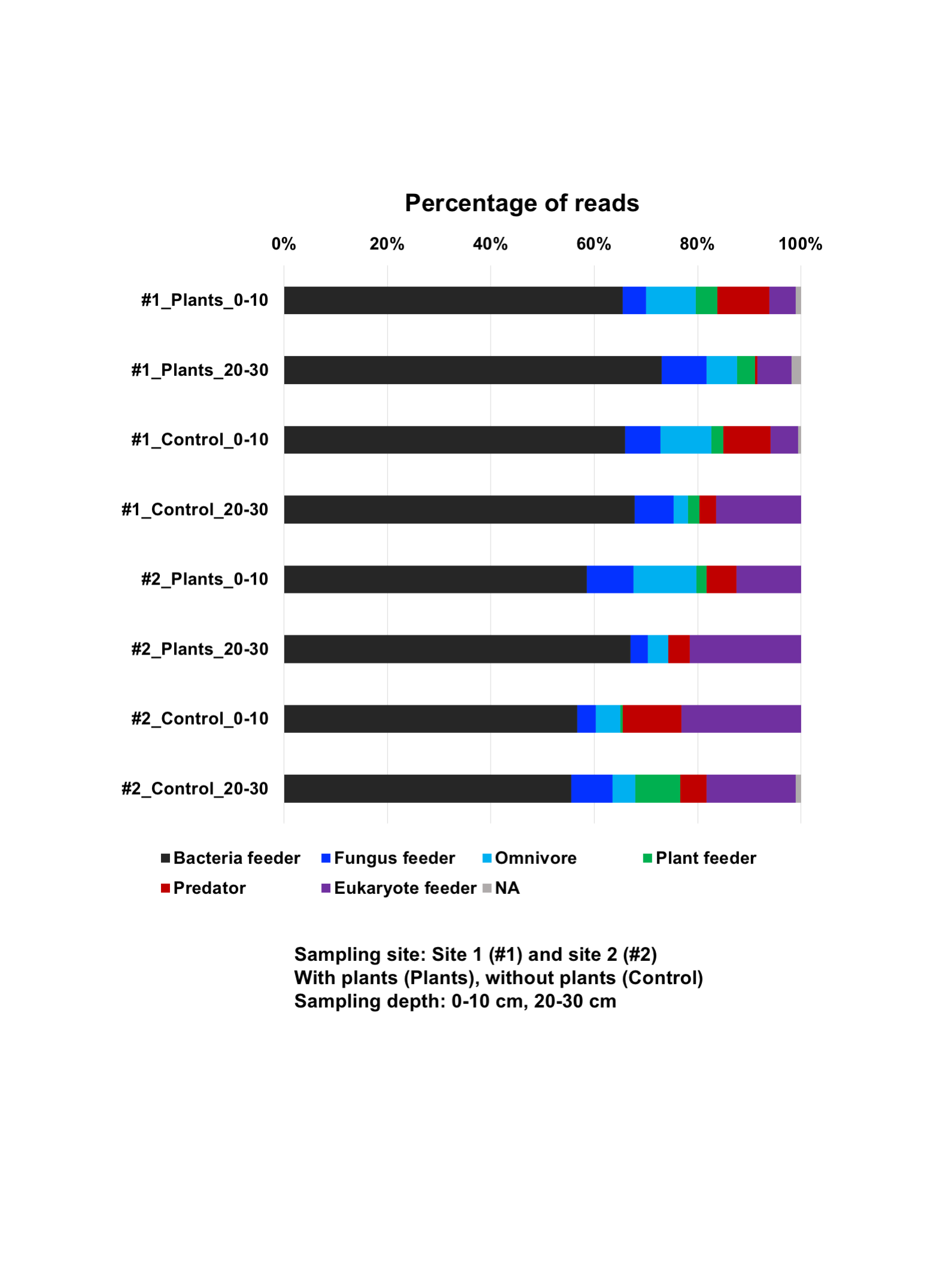

Supplement: S12 Fig — Feeding types of the nematode-derived SVs identified from eight soil samples were assigned as described in the legend for S11 Fig. The percentages of the sequence reads of nematode-derived SVs in feeding type are shown by colored fractions of horizontal bars in eight soil samples isolated from the surface (0–10) and deep (20–30) layers at the sampling point with (plants) and without (control) the growing sweet potato at site 1 (#1) and 2 (#2), respectively, as shown in the left of the corresponding histogram. Each feeding type is indicated by color as shown at the bottom of figure. The fractions of SVs with multiple feeding types are classified as “not assigned (NA)” and indicated in light gray. (TIFF) [file pone.0259842.s022.tiff]

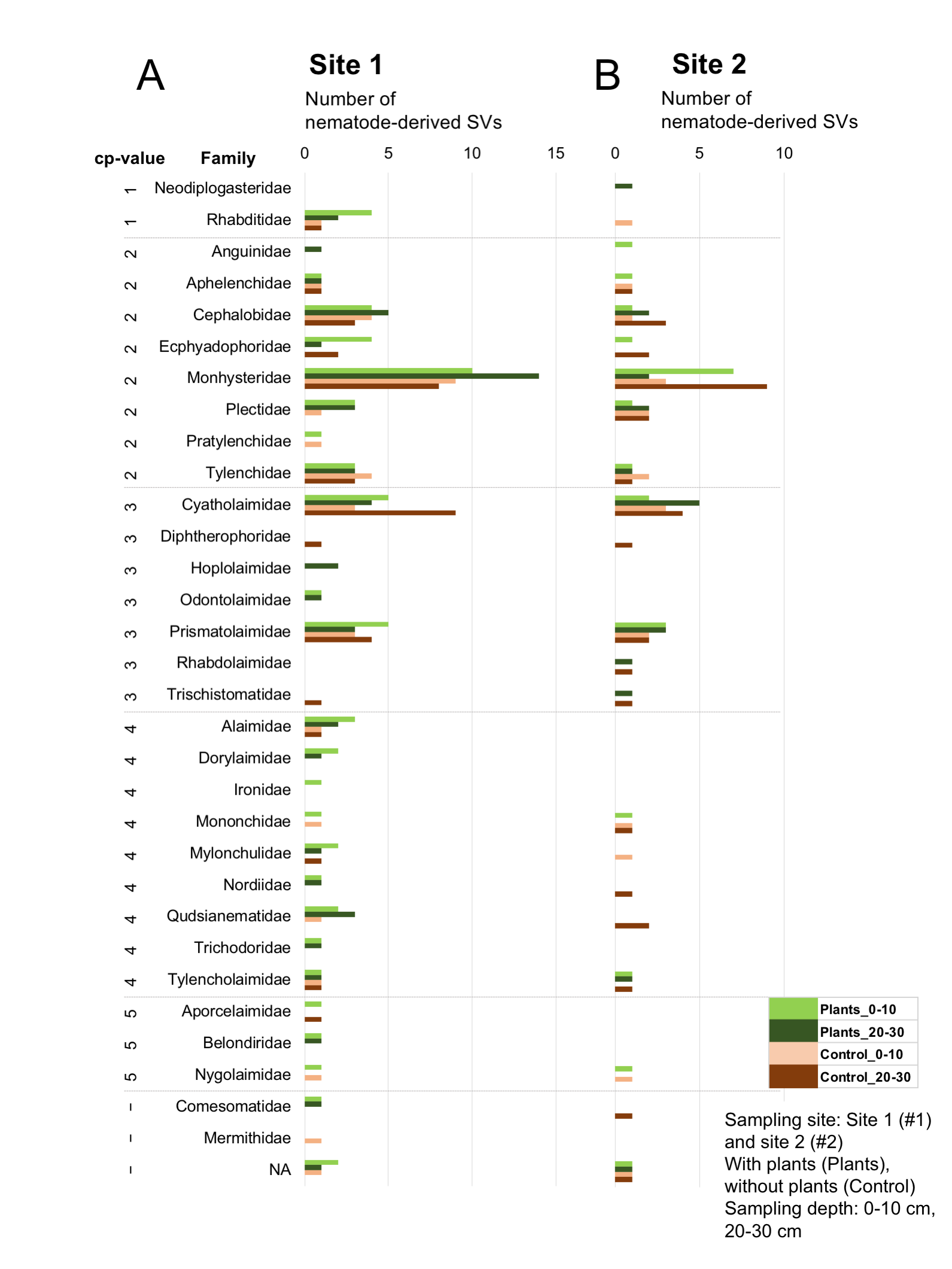

Supplement: S13 Fig — Nematode-derived SVs identified from soil DNAs derived from sites 1 (A) and 2 (B) of the agricultural field were assigned to nematode families based on the closest species identified by the BLASTN search. Nematode-derived SVs identified from four soil samples: The surface (0–10) and deep (20–30) layers at the sampling point with (plants) and without (control) the growing sweet potato, respectively, as shown by colors in the legend box. Number of nematode-derived SVs in family is shown by the corresponding color bar in each sample. Families are aligned by their cp values (1–5); undefined cp values are indicated by a hyphen (-). NA: not assigned to a single family. (TIFF) [file pone.0259842.s023.tiff]

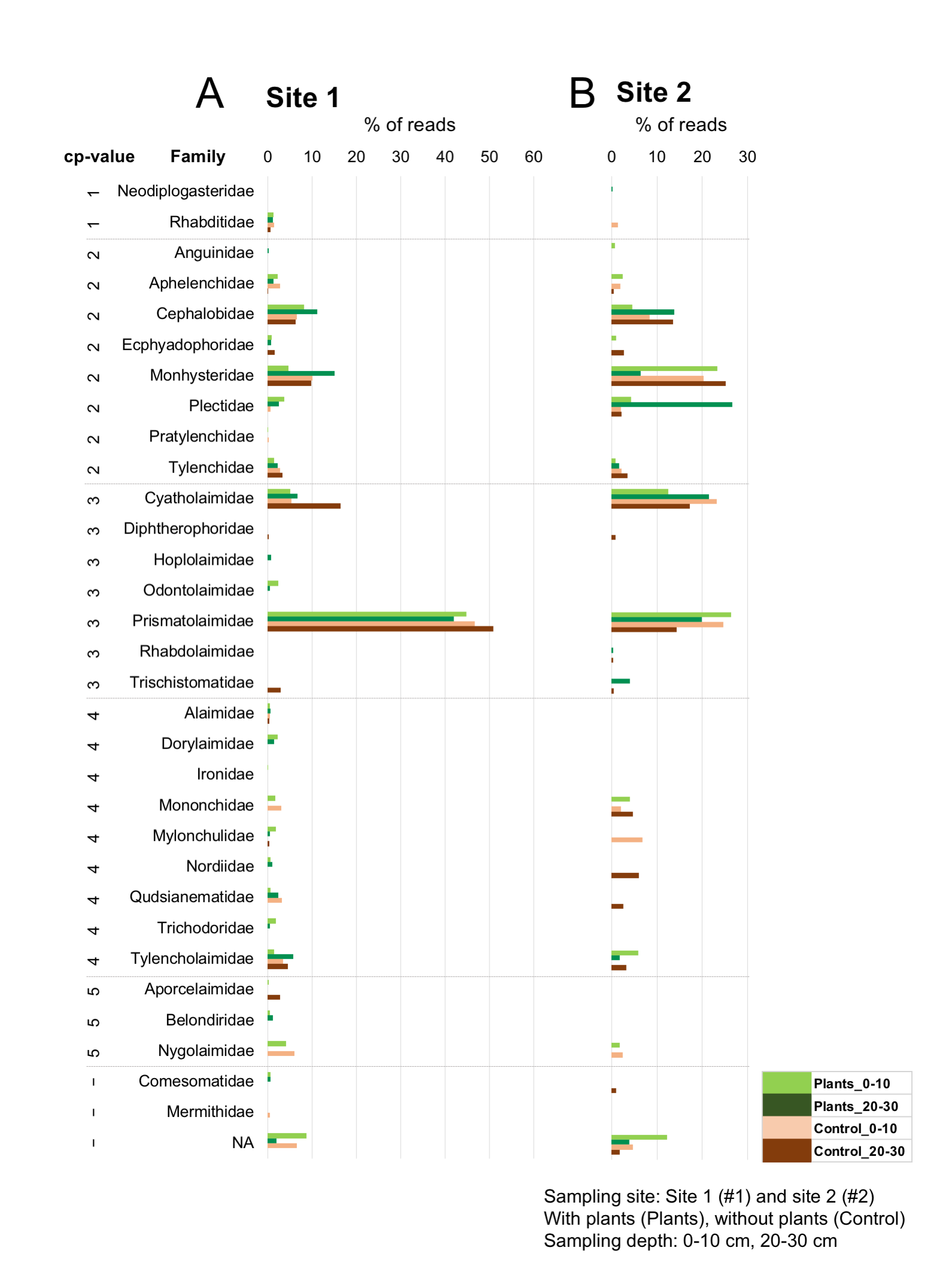

Supplement: S14 Fig — The relative abundance (%) of sequence reads of SVs obtained from four soil samples at sites 1 (A) and 2 (B) in each family are shown by colored horizontal bars. Soil samples were isolated from the surface (0–10) and deep (20–30) layers at the proximal (plants) and distal (control) to the sweet potato at each site, as shown by colors in the legend box. Families are aligned by their cp values (1–5); undefined cp values are indicated by a hyphen (-). NA: not assigned to a single family. (TIFF) [file pone.0259842.s024.tiff]
